# Study on the Mechanism of Compound Kidney-Invigorating Granule for Osteoporosis based on Network Pharmacology and Experimental Verification

**DOI:** 10.1155/2022/6453501

**Published:** 2022-01-04

**Authors:** Hao Lv, Jiuxiang Wang, Yujun Zhu, Ting Jiang

**Affiliations:** ^1^The First Affiliated Hospital of Anhui University of Chinese Medicine, Hefei 230031, China; ^2^Anhui University of Chinese Medicine, Hefei 230031, China

## Abstract

**Background:**

This study used a combination of network pharmacology and experimental confirmation to clarify the mechanism of the compound kidney-invigorating granule (CKG) in treating osteoporosis (OP).

**Methods:**

The main bioactive compounds and corresponding targets of CKG were collected and screened via the Traditional Chinese Medicine Systems Pharmacology Database and Analysis Platform (TCMSP), Yet another Traditional Chinese Medicine (YaTCM), and UniProt databases. Disease targets of OP were summarized in GeneCards and the Comparative Toxicogenomics Database (CTD). Targets of CKG for OP were obtained by Venn diagram. The protein-protein interaction (PPI) network was constructed by the STRING database and then screened for hub genes through Cytoscape 3.7.2 software. The Gene Ontology (GO) and the Kyoto Encyclopedia of Genes and Genomes (KEGG) enrichment were analyzed and visualized by R software. Then, CB-Dock was used for molecular docking verification. Finally, we confirmed the antiosteoporosis effect of CKG through animal and cell experiments.

**Results:**

A total of 250 putative targets were obtained from 65 bioactive compounds in CKG. Among them, 140 targets were related to OP. Topological analysis of the PPI network yielded 23 hub genes. Enrichment analysis showed the targets of CKG in treating OP might concentrate on the MAPK signaling pathway, the TNF signaling pathway, the PI3K-Akt signaling pathway, etc. The results of molecular docking showed the bioactive components in CKG had good binding ability with the key targets. The experimental results showed that CKG-medicated serum had a promoting effect on proliferating hBMSCs, increasing the expression of AKT, PI3K, ERK1, and IkB in cells and decreasing the expression of IKK in cells.

**Conclusion:**

CKG has a complex of multicomponent, multitarget, and multipathway. This study lays the theoretical foundation for further *in vitro* and *in vivo* experimental studies and further expands the clinical applications of CKG.

## 1. Introduction

Osteoporosis (OP) is a systemic metabolic bone disease characterized by low bone mass, low bone density, and bone microstructure destruction, which leads to increased bone fragility and susceptibility to bone fracture. OP is considered a “silent disease” since it progresses without symptoms until a fracture occurs [1]. In the 2010 U.S. census, around 10.2 million adults aged 50 and older were living with OP [2]. An estimated 10.9 million males are suffering from OP in China, while the number of females suffering from OP is 49.3 million [3]. The incidence of OP increases yearly with the aging of the general population. Current treatments for osteoporosis include bisphosphonates, raloxifene, calcium, and vitamin D [4]. Nevertheless, these approved drugs have also shown significant side effects, such as the increased risk of hypercalcemia and hypercalciuria due to long-term vitamin D supplementation [5]. Since bisphosphonates are incorporated within the bone matrix with high affinity, long-term treatment with bisphosphonates may lead to atypical femur fractures [6]. Raloxifene is associated with adverse effects such as thromboembolism, pulmonary embolism, and cerebrovascular death [7].

Compared with Western medicine, traditional Chinese medicine (TCM) has unique advantages in treating OP, such as low cost, multilevel, and prominent curative effect. Therefore, TCM is gradually attracting considerable attention from the international medical community and is accepted by non-Chinese as an additional and alternative medical treatment for OP due to these characteristics. Clinically, OP is regarded as “bone atrophy” and “lack of bone marrow” in TCM. According to TCM syndrome differentiation, OP is divided into kidney Yang deficiency syndrome, blood stasis syndrome and kidney deficiency syndrome, blood stasis and Qi stagnation syndrome, liver and kidney Yin deficiency syndrome, spleen and kidney Yang deficiency, and asthenia of the spleen and stomach syndrome.

Compound kidney-invigorating granule (CKG) is a TCM formula commonly used at the First Affiliated Hospital of Anhui University of Chinese Medicine. CKG is an effective cure for the OP of blood stasis and kidney deficiency syndrome. It is composed of six kinds of traditional Chinese drugs, namely, Radix Astragali (RA, Huang-qi in Chinese), Radix Paeoniae Alba (RPA, Bai-shao in Chinese), Radix Cyathulae (RC, Chuan-niu-xi in Chinese), Herba Epimedii (HE, Yin-yang-huo in Chinese), Caulis Polygoni Multiflori (CPM, Ye-jiao-teng in Chinese), and Concha Ostreae (CO, Mu-li in Chinese). Preliminary clinical studies have shown that CKG can significantly improve the clinical symptoms, bone mineral density (BMD), and visual analog scale (VAS) in postmenopausal nonelderly patients with OP. There were no adverse reactions that occurred during the study, and there were no significant abnormalities in the safety observation index. Therefore, CKG is safe and effective in treating postmenopausal nonelderly osteoporosis [8]. However, the pharmacological mechanism of CKG in treating OP is unknown.

TCM has become a significant source that provides potential lead compounds for drug development, especially those used to treat complex diseases, owing to its multicomponent and multitarget characteristics [9]. Guided by holistic thinking, TCM exerts unexpected curative effects in difficult and complicated diseases that Western medicine finds difficult to tackle [10]. However, due to the complex components of TCM prescriptions, traditional pharmacological methods to find out their unique actions through experiments may not be suitable for TCM research [11]. Western drug pharmacology molecular research may be stuck in a problem when applied to TCM prescriptions since it clarifies single or limited biological molecules from the perspective of reductionism instead of considering the overall theory of the organism [12]. On the contrary, network pharmacology uses the “target network, multi-components” research model to replace the traditional “one target, one drug” research model. Therefore, network pharmacology conforms to the core of the overall philosophy of TCM [13]. This combination of pharmacokinetic evaluation and bioinformatics has made a significant contribution to the study of the molecular of TCM [14]. People apply the strategy to the pharmacological research of TCM compounds and explain the complex relationship between biological and herbal prescriptions [12]. Accordingly, regarding the unknown mechanism of CKG, this study adopted the method of network pharmacology to explore the molecular biology mechanism of CKG in treating OP.

**Figure 1:**
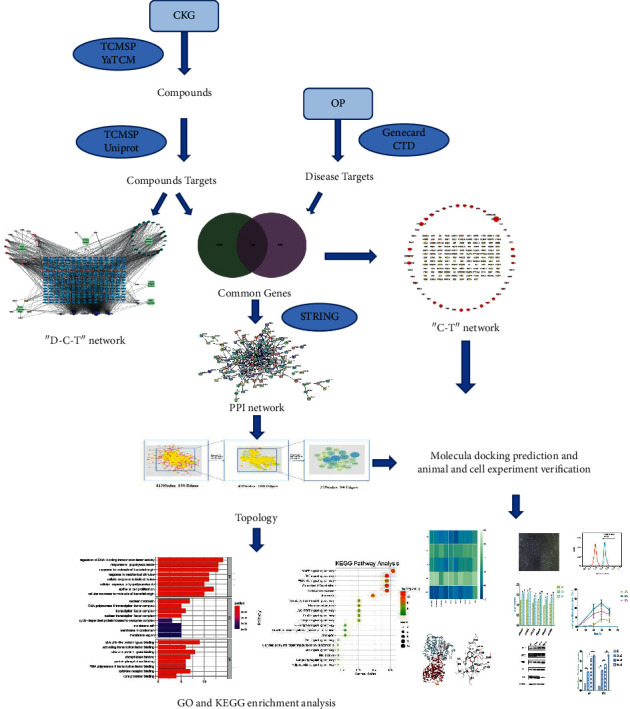
The technical strategy of the current study.

The present study uses various platforms, databases, and tools to predict the bioactive compounds, potential targets, and pathways of CKG, aiming to explore the pharmacological network of CKG on OP and verify it through related experiments.  Figure 1 shows the detailed technical strategy of the research.

## 2. Materials and Methods

### 2.1. Collection and Screening of Bioactive Ingredients

The bioactive ingredients of CKG were identified by the Traditional Chinese Medicine Systems Pharmacology Database and Analysis Platform (TCMSP) (https://tcmspw.com/tcmsp.php) [15]. It compensates the insufficient information in the Yet another Traditional Chinese Medicine (YaTCM) database (https://cadd.pharmacy.nankai.edu.cn/yatcm/home) [16]. Oral herbal medicines must overcome obstacles in absorption, distribution, metabolism, and excretion (ADME) to be effective. In this process, oral bioavailability (OB) is one of the most important pharmacokinetic parameters [17]. As a qualitative concept, drug-like quality (DL) is used in drug design for the evaluation of the drug ability of a molecule and is utilized to optimize the pharmacokinetics and properties of a drug [18]. This study chose OB ≥ 30% and DL ≥ 0.18 as the criteria for screening bioactive ingredients. The OB and DL indicators of each ingredient in CKG were retrieved from the TCMSP database.

### 2.2. Prediction of Drug Targets for CKG

The protein targets of the screened bioactive ingredients of CKG were obtained from the TCMSP database. The obtained protein targets were converted into gene targets in the Universal Protein Resource (UniProt) database (https://www.uniprot.org/) [19], a comprehensive database of protein sequence and annotation data.

### 2.3. Construction of Drug-Compound-Target Network

The obtained bioactive ingredients, gene targets, and herbs were entered into Cytoscape 3.7.2 software (https://www.cytoscape.org/) for data visualization and construction of the “D-C-T” network [20]. The network nodes represent the herbs, gene targets, or bioactive components, and the edges indicate their interaction.

### 2.4. Collection of Gene Targets for OP

OP-related human gene targets were obtained from two databases: GeneCards (https://www.genecards.org/) and the comparative toxicogenomics (CTD) database (https://ctdbase.org/). The term “Osteoporosis” was used as the keyword for the search [21, 22].

### 2.5. Collection of Relevant Targets for Formula Treatment of Diseases

The results of gene targets of CKG were matched with the disease-associated genes of OP. The jvenn (https://www.bioinformatics.com.cn/static/others/jvenn/) was used to realize the visualization of the drug-disease target and to draw Venn diagrams [23]. Then, the intersecting gene targets of CKG and OP were collected as the relevant targets of CKG for treating OP, which might be the potential target set of CKG in treating OP.

### 2.6. Construction of a Compound-Target Network That Bioactive Ingredients of Formula with Intersecting Gene Targets

The obtained bioactive ingredients and intersecting gene targets were imported into Cytoscape 3.7.2 software for data visualization and construction of the “C-T” network. The nodes of the network represented the intersecting gene targets and bioactive components, and the edges indicated their interaction.

### 2.7. Protein-Protein Interaction (PPI) Network Construction and Core Gene Screening

The common gene targets of CKG and OP were entered into the STRING database (https://string-db.org/) [24]. The selection parameter was set to “Homo sapiens” for species, the confidence level was set at 0.950 for the minimum required interaction score, and hid disconnected nodes in the network. The PPI network analysis results were downloaded in TSV format. The file was imported into Cytoscape software for topological property analysis. Then, this study evaluated the topological properties of the nodes in the interaction network by calculating three parameters, namely, “degree centrality (DC),” “betweenness centrality (BC),” and “closeness centrality (CC)” via the App CytoNCA [25]. These three parameters measure the importance of nodes in the network and indicate the nature of the nodes in the network. Nodes with high DC, BC, and CC implied that they were playing an essential role in the network. We obtained the core genes in the network by screening above the median value twice based on the results of the topological property analysis of PPI.

### 2.8. Gene Ontology (GO) and the Kyoto Encyclopedia of Genes and Genomes (KEGG) Enrichment Analysis

Various bioinformatics analyses and visualization of the results can be achieved through the software R project (https://www.r-project.org/). Firstly, the ENSMBL gene ID of each core gene was obtained from RStudio, AnnotationHub, and org. Hs.eg.db, and then the GO function enrichment (molecular function, MF; biological process, BP; and cellular component, CC) and KEGG enrichment were enriched by RStudio, DOSE, and Cluster Profiler. In the programming language, pvalueCutoff = 0.05 and qvalueCutoff = 0.05 were set. The results of the analysis selected the top 10 items of BP, MF, and CC in GO enrichment and the top 20 filtered pathways most closely related to OP, then displayed the results of the GO enrichment analysis as a bar graph and the results of the KEGG enrichment analysis as a bubble graph.

### 2.9. Molecular Docking Verification

Since there is no uniform standard for target screening of bioactive molecules, the 5 proteins with the highest degree of PPI network were selected for molecular docking with the 10 molecules with the highest degree of the “C-T” network. The bioactive compounds were downloaded as 2D structure files using the PubChem database (https://pubchem.ncbi.nlm.nih.gov/), converted into a 3D structure with ChemBio3D Ultra 14.0, and saved as a mol2 format file [26], whereas the RCSB PDB database (https://www.rcsb.org/) was used to retrieve and download the files in “PDB” format of the core target proteins [27]. Then, the proteins and molecules files were imported into the CB-Dock website (https://clab.labshare.cn/cb-dock/php/index.php) for molecular docking. Finally, the docking model with the lowest binding energy was selected and visualized.

### 2.10. Experimental Verification

#### 2.10.1. Materials

*(1) Medicinal Materials*. All Chinese medicines of CKG were purchased from the First Affiliated Hospital of Anhui University of Traditional Chinese Medicine. The prescription medicinal materials of each dose of CKG are HE (20 g), RC (15 g), RA (18 g), CO (10 g), RPA (20 g), and CPM (25 g), 30 doses in total.

*(2) Antibodies and Reagents*. The following antibodies and reagents were used for this study: high-glucose Dulbecco minimum essential medium (HG-DMEM) and fetal bovine serum (FBS) (Gibco, Rockville, MD, USA); 0.25% trypsin-EDTA solution, penicillin-streptomycin, and phosphate-buffered saline (PBS) (Hyclone, Logan, UT, USA); protein extraction kit (KEYGEN, Nanjing, China); AKT, PI3K, ERK1, IkB, IKK antibody, and GAPDH antibody (Wanleibio, Shenyang, China); CD29 antibody, CD34 antibody, and CD44 antibody (Abcam, Cambridge, MA, USA); and cell counting kit-8 (CCK-8) (Beyotime Biotechnology, Shanghai, China).

*(3) Instrument*. The following instruments were used for this study: CO_2_ incubator (NuAire, MN, USA), Multiskan spectrum (BioTek Instruments, VT, USA), flow cytometer (Beckman Coulter, CA, USA), high-performance centrifuge (Eppendorf, Hamburg, German), western blot system (Tanon Science & Technology, Shanghai, China), ice machine (XUEKE ELECTRIC, Changshu, China), inverted microscope (Olympus Corporation, Tokyo, Japan), cell counter (Countstar, Shanghai, China), clean bench (AIRTECH, Suzhou, China), cell culture plates, and cell culture flasks (Corning, NY, USA).

#### 2.10.2. Isolation, Culture, and Identification of Cells

This investigation was approved by the Ethics Committee of the Anhui University of Chinese Medicine. Written informed consent was obtained from all participants before the study. Bone marrow was obtained from OP patients who underwent surgery in the orthopedics department of the First Affiliated Hospital of Anhui University of Traditional Chinese Medicine and volunteered to participate in this study. Separating and culturing hBMSCs (human bone marrow mesenchymal stem cells) via the method of whole bone marrow adherent. Bone marrow samples applied in cell culture plates were cultured in HG-DMEM containing 10% FBS, 100 U/mL penicillin, and 100 *μ*g/mL streptomycin, then cultured in a 37°C incubator with a 5% CO_2_ atmosphere. The medium was changed every 2 days until the cells became confluent, about 80%–90%. The cells were then passaged. The surface marker phenotypes (CD29+, CD44+, and CD34−) were characterized by using flow cytometry. After the cells were confirmed to be hBMSCs, cells from passages 3 to 5 were used in subsequent experiments.

#### 2.10.3. Preparation and Preservation of CKG-Medicated Serum

The experiment was approved by the Animal Ethics Committee of the Anhui University of Chinese Medicine. Twenty-four 4-week-old SPF Sprague–Dawley rats weighing 200–250 g, half male and half female, were purchased from the Experimental Animal Center of Anhui Medical University (Experimental Animal Production License Number: SCXK (Wan) 2017-001). Animals were placed in plastic cages at 22 ± 1°C with a 12 h light-dark cycle. Food and water were available ad libitum. They adapted for a week in this environment. The decoction method of the CKG prescription is as follows: after 60 minutes of soaking in sufficient water, the original drugs with the water were decocted in a Chinese herbal medicine decoction machine, then brought to a boil over high heat, and then simmered for 1 hour. The process was repeated three times. The decocting solution was pooled together, added into an electrothermal constant temperature water tank, and concentrated to 2.9 g of the original drugs per mL. The samples were sealed and stored in a refrigerator at 4°C. Animals were randomly divided into medicated serum (MS) (*n* = 12) and nonmedicated serum (NS) (*n* = 12) groups. MS group animals were administered intragastrically with CKG concentrated liquor (20 mL/kg) once a day for 7 days. NS group animals were administered intragastrically saline on the same schedule. 12 h before the final treatment, fasting but not watering. 1 h after the final treatment, rats were weighed and intraperitoneally anesthetized using 1% pentobarbital sodium (60 mg/kg). Blood was collected from the abdominal aorta. The collected blood was allowed to stand at room temperature for one hour, then centrifuged at 3000 r/min for 15 min, and the upper serum was taken from the ultraclean workbench. Serum samples from all individual animals of each group were pooled. Then, it was filtered through a 0.22 *μ*m filter membrane, heat-inactivated at 56°C for 30 min, and stored at −80°C until used.

#### 2.10.4. Detect the Promoting Effect of CKG-Medicated Serum on Cell Proliferation

Cells were collected in the logarithmic growth phase and a single cell suspension was prepared. Cells were seeded into 96-well microplates at 1 × 10^4^ cells/well in 200 *μ*L medium after counting with a cell counter. NS and MS (at concentrations of 2, 5, and 8%) were added to the culture medium for 24 h. The culture medium was then removed and 100 *μ*L of CKK-8 solution was added to the well and incubated for 24 h, 36 h, and 48 h. Subsequently, the CKK-8 solution was removed, and 100 *μ*L of DMSO was added to dissolve the formazan crystals. The optical density (OD) value was read at 450 nm by using a microplate reader. Cell proliferation rate = (OD_MS_ − OD_NS_)/OD_NS_.

#### 2.10.5. Western Blot

hBMSCs were plated into 6-well plates. After 12 h, they were treated with MS (at concentrations of 0, 2, 5, and 8%). The treating time is decided by the results of 2.9.3. Total proteins were extracted from the protein extraction kit, separated using SDS-PAGE, and then transferred to polyvinylidene difluoride (PVDF) membranes, which were blocked with 5% nonfat dry milk in TBST buffer. The primary antibodies were added at proper dilution and incubated at 4°C overnight, and the secondary antibody was added and incubated for 60 min, PVDF membranes were washed with precooled PBS three times. Finally, the signals for the immunoreactive proteins were visualized by enhanced chemiluminescence reagents and then analyzed with Quantity One software. The experiment was repeated 3 times independently.

## 3. Results

### 3.1. Identification of Bioactive Components in CKG

The screening criteria were OB ≥ 30% and DL ≥ 0.18. We got 76 bioactive ingredients via the TCMSP database, including 23 of RA, 16 of RPA, 5 of RC, 26 of HE, 3 of CPM, and 3 of CO. 65 bioactive ingredients were obtained after the removal of duplicate components. Results are detailed in Table 1. Sitosterol is the common bioactive ingredient of RA, RPA, and HE. Kaempferol is the common bioactive ingredient of RA, RPA, HE, and CPM. Mairin is the common bioactive component of RA and RPA. Quercetin is the common bioactive component of RA, HE, CPM, and RC. 22, 23-Dihydrostigmasterol is the common bioactive ingredient of RPA, HE, and RC.

### 3.2. Acquisition of Drug Targets and Construction of the “D-C-T” Network

**Figure 2:**
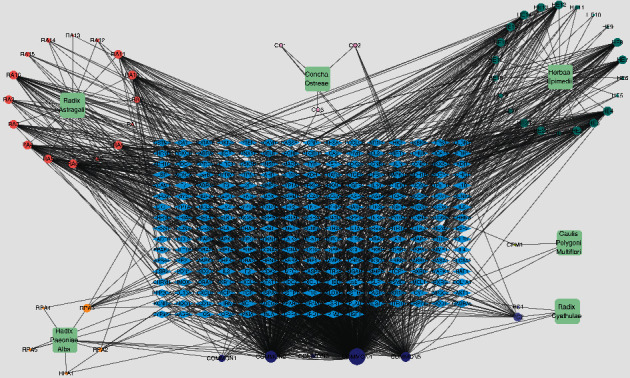
drug-compound-target network. Blue diamond nodes indicate targets, cyan square nodes indicate herbs, coral red round nodes indicate bioactive ingredients of RA, chrome yellow round nodes indicate bioactive ingredients of RPA, dark green round nodes indicate bioactive ingredients of HE, lilac round nodes indicate bioactive ingredients of CO, bright yellow round nodes indicate bioactive ingredients of CPM, lavender round nodes indicate bioactive ingredients of RC, and dark purple round nodes indicate bioactive ingredients of herbs together. The size of the compound-related nodes indicates the size of the degree value.

By searching targets of the bioactive ingredients in the TCMSP database and performing protein-gene named transformations in the UniProt database, we got 1641 targets in this study. After removing duplicate data, 250 targets were obtained. After removing the bioactive ingredients without targets, they successfully constructed the “D-C-T” network by inputting the files containing drugs, bioactive ingredients with targets, and gene targets into the Cytoscape software. The squares represent drugs, the rounds represent bioactive ingredients, and the diamonds represent various targets. There are 310 nodes and 1705 edges in the “D-C-T” network.  Figure 2 shows the network.

### 3.3. Acquisition of Disease Targets, Intersection Targets, and Construction of the “C-T” Network

**Figure 3:**
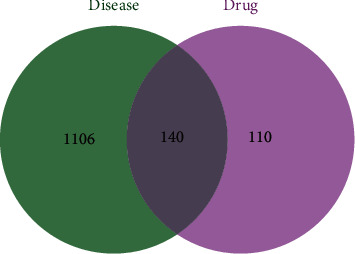
Drug-disease targets Venn diagram.

**Figure 4:**
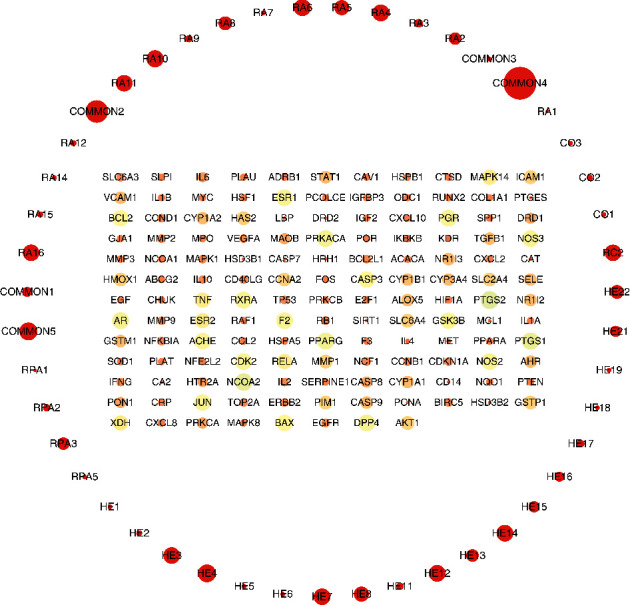
The compound-target network. The colored circular nodes indicate the intersecting gene targets, and the red circular nodes indicate the bioactive components of CKG. The size of the nodes indicates the size of the degree value.

By searching and integrating them in the GeneCards and CTD databases, we got 1246 OP gene targets. By taking the intersection of targets of OP with targets of CKG, we got 140 intersecting genes.  Figure 3 shows the drug-disease targets Venn diagram. The “C-T” network was constructed by entering 140 intersecting genes and bioactive ingredients into Cytoscape software. There are 187 nodes and 1028 edges in the “C-T” network.  Table 2 shows the degree values of the compounds in the “C-T” network. Results are as detailed in Supplementary Table 1.  Figure 4 shows the network.

### 3.4. Construction, Topology of PPI Network, and Acquisition of Core Genes

**Figure 5:**
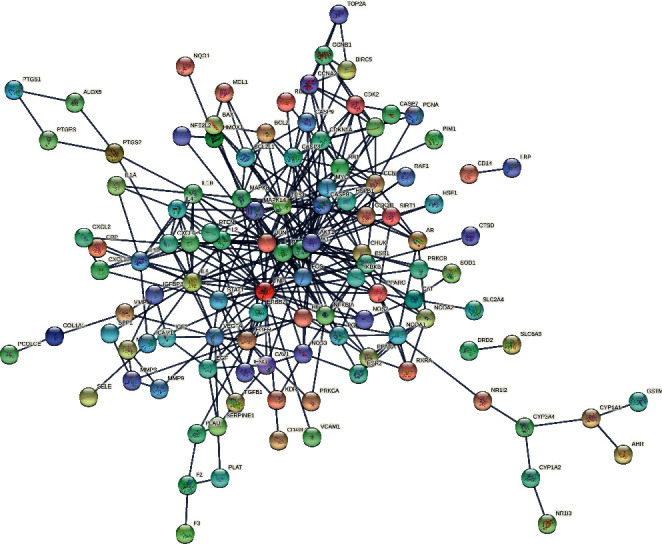
The protein-protein interaction network of targets of CKG against OP.

**Figure 6:**
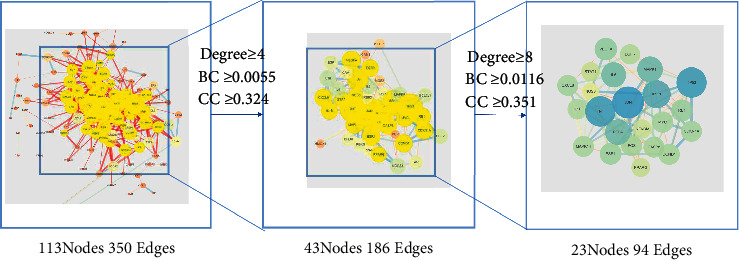
The process of topological screening for the PPI network.

**Figure 7:**
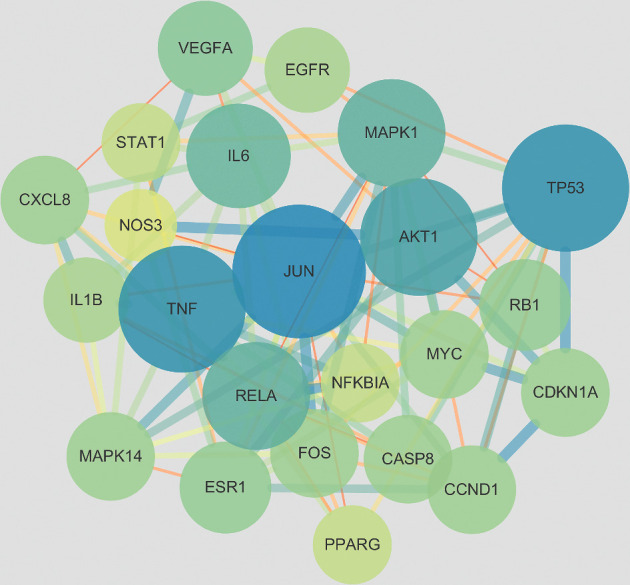
The network of the top 23 hub genes.

We imported the obtained intersecting genes into the STRING database to acquire the interaction relationships between them and saved the data as a file in TSV format.  Figure 5 shows the PPI network diagram. We imported the TSV data into Cytoscape software. The network has 113 nodes and 350 edges. Then they used CytoNCA to analyze the PPI network based on DC, BC, and CC parameters. The criteria for the first screening were Degree ≥4, BC ≥ 0.0055, and CC ≥ 0.324. The results showed 43 nodes and 186 edges. The second screening threshold was Degree ≥8, BC ≥ 0.0116, and CC ≥ 0.35. The second screening result was 23 nodes and 94 edges.  Figure 6 shows the process of topology. The core genes were JUN, TP53, TNF, AKT1, MAPK1, RELA, IL6, VEGFA, RB1, ESR1, CDKN1A, MYC, CCND1, CASP8, MAPK14, FOS, CXCL8, IL1B, EGFR, NFKBIA, STAT1, PPARG, and NOS3.  Figure 7 shows the hub genes.  Table 3 shows the top 5 degree values of hub genes.

### 3.5. Enrichment Analysis of GO and KEGG

**Figure 8:**
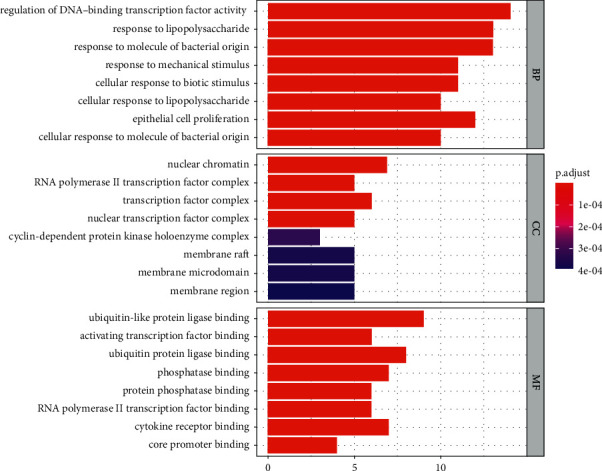
GO enrichment analysis.

**Figure 9:**
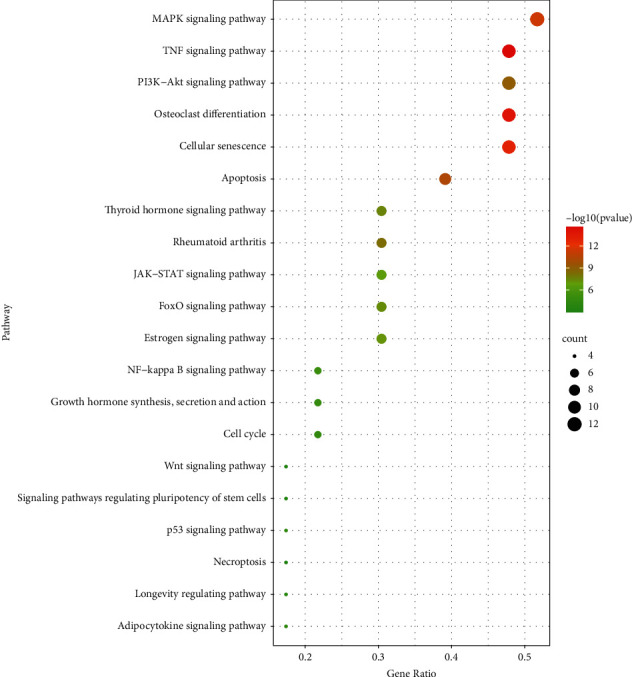
KEGG enrichment analysis.

GO enrichment analysis got 1723 entries, of which 1621 were BP entries, 20 were CC entries, and 82 were MF entries. BP analysis showed the targets principally relate to regulating DNA-binding transcription factor activity, response to lipopolysaccharide, response to molecules of bacterial origin, response to mechanical stimulus, cellular response to biotic stimulus, cellular response to lipopolysaccharide, epithelial cell proliferation, cellular response to molecules of bacterial origin, response to reactive oxygen species, and muscle cell proliferation. Analysis of the CC category showed the targets mostly within nuclear chromatin, the RNA polymerase II transcription factor complex, the transcription factor complex, the nuclear transcription factor complex, the cyclin-dependent protein kinase holoenzyme complex, membrane raft, membrane microdomain, membrane region, the serine/threonine-protein kinase complex, the transferase complex, and the transferring phosphorus-containing groups. The MF of these proteins includes ubiquitin-like protein ligase binding, activating transcription factor binding, ubiquitin protein ligase binding, phosphatase binding, protein phosphatase binding, RNA polymerase II transcription factor binding, cytokine receptor binding, core promoter binding, DNA-binding transcription activator activity, RNA polymerase II-specific, and repressing transcription factor binding. The results of the GO analysis are as shown in Figure 8. The KEGG enrichment analysis yielded 180 entries, including the MAPK signaling pathway, the TNF signaling pathway, the PI3K-Akt signaling pathway, osteoclast differentiation, the NF-kappa B signaling pathway, and the Wnt signaling pathway.  Figure 9 shows the top 20 filtered pathways most closely related to OP for the KEGG enrichment analysis. According to the KEGG analysis of the hub genes, we obtained the signal pathway diagrams of the MAPK signaling pathway, the PI3K-Akt signaling pathway, and the TNF signaling pathway. Comprehensively analyzing the three pathways, we can conclude that these pathways are all regulated by Akt, which can negatively regulate Raf, thereby affecting the Ras/MAPK signaling pathway. Akt regulates NF-kB by phosphorylating IKK and is directly involved in the regulation of the PI3K-Akt signaling pathway. The diagrams of the three signal pathways and their interconnection are stored in Supplementary Table 5.

### 3.6. Molecular Docking Analysis

**Figure 10:**
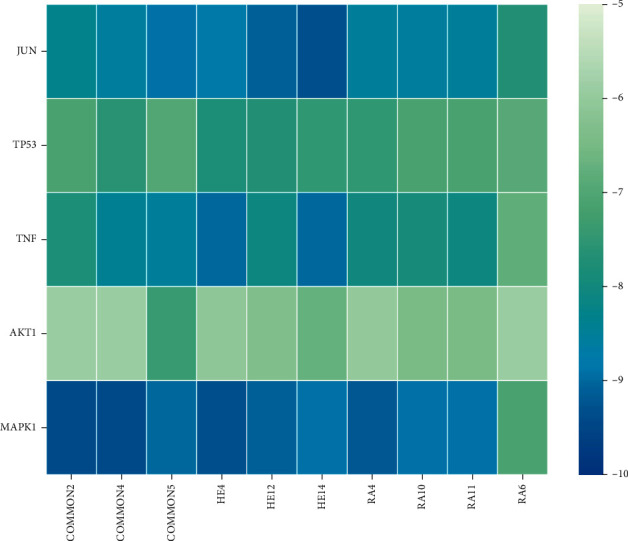
Heat maps of molecular docking between bioactive ingredients of CKG and core targets.

**Figure 11:**
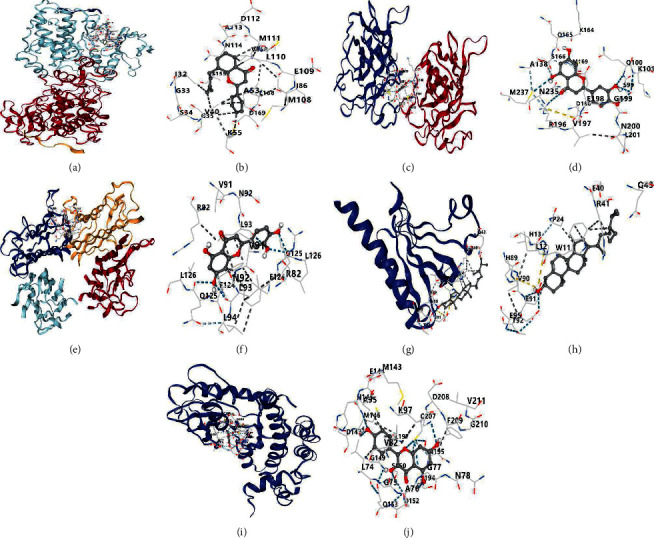
Binding mode and a 2D interaction map of compounds with core targets. (a) The binding mode of 8-(3-methylbut-2-enyl)-2-phenyl-chromone with JUN; (b) the 2D interaction map of 8-(3-methylbut-2-enyl)-2-phenyl-chromone with JUN; (c) the docking process of luteolin with TP53; (d) the 2D interaction map of luteolin with TP53; (e) the binding mode of luteolin with TNF; (f) the 2D interaction map of luteolin with TNF; (g) the binding mode of 22,23-dihydrostigmasterol with AKT1; (h) the 2D interaction map of 22,23-dihydrostigmasterol with AKT1; (i) the binding mode of kaempferol with MAPK1; and (j) the 2D interaction map of kaempferol with MAPK1.

Five core targets JUN, TNF, TP53, Akt1, and MAPK1 were selected based on the degree values, and then molecularly docked with the core bioactive ingredients kaempferol, quercetin, 22,23-dihydrostigmasterol, luteolin, anhydroicaritin, 8-(3-methylbut-2-enyl)-2-phenyl-chromone, isorhamnetin, formononetin, calycosin, and 7-O-methylisomucronulatol obtained by swiping the degree values. It is generally accepted that for a protein-ligand complex, the lower the binding energy, the higher the binding affinity. We set binding energy ≤ −5.0 kJ/mol as the standard.  Figure 10 shows the results of molecular docking. The results in Figure 10 show that the minimum binding energy of all selected bioactive ingredients to the receptor is much less than −5.0 kJ/mol.  Table 4 shows the specific information of the lowest binding energy among the 10 active ingredients docked with each core target.  Figure 11 shows the visualization of the docking results. The molecular docking results show the vital bioactive ingredients have excellent binding to the core targets for OP treatment.

### 3.7. Experimental Verification

#### 3.7.1. Characteristics and Identification of hBMSC Phenotypes

**Figure 12:**
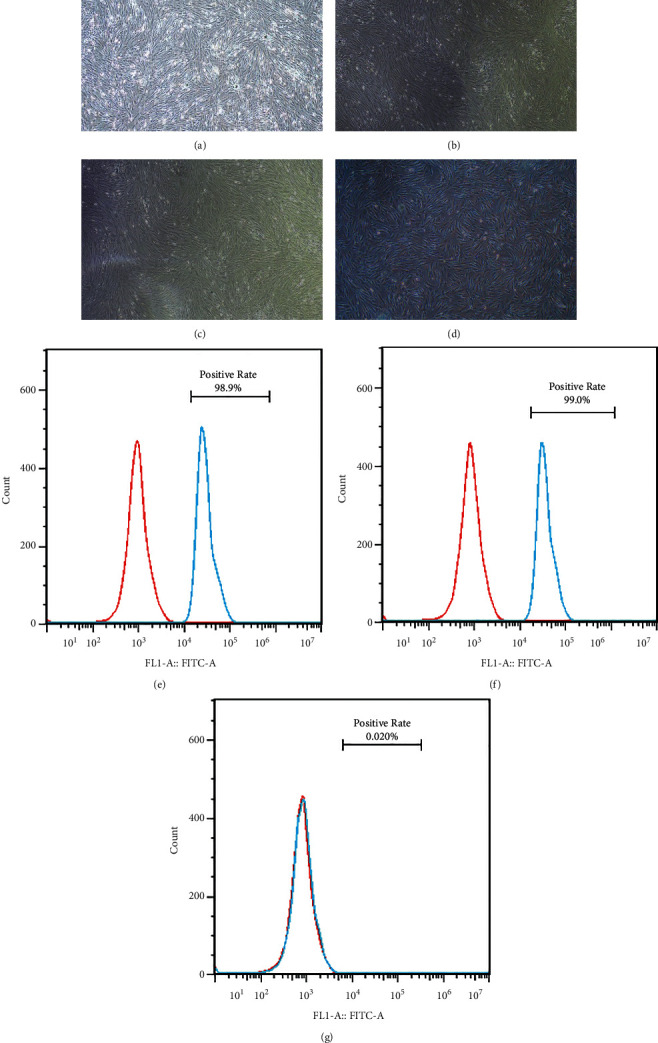
Identification of hBMSCs. hBMSCs at passage 4 were identified with flow cytometry. (a–d) The cell morphology of passage 1 (day 6), 2 (day 5), 3 (day 5), and 4 (day 4), respectively. Flow cytometry was used to analyze the levels of the biomarkers in hBMSCs (e–g). The hBMSCs were positive for CD29 (e), CD44 (f), and negative for CD34 (g).

All primary cell cultures were used at early passages (3–5 passage), as shown in Figures 12(a)–12(d)). After 6 days of primary culture, hBMSCs had attached, exhibited spindle and flat shapes, and reached a confluence of around 70%. Passage 2 and 3 hBMSCs reached 85% confluence on day 5 after passaging. Passage 4 hBMSCs reached 85% confluence on day 4 after passaging and resembled a shoal of fish. As shown in Figures 12(e)–12(g), P4 cells were detected with flow cytometry. Notably, the cells were positive for the typical hBMSCs markers CD29 (98.9%) and CD44 (99%), with concomitant absence of CD34 (0.02%). Therefore, we concluded that most of the isolated and purified cells expressed standard markers of hBMSCs.

#### 3.7.2. The Promoting Effect of CKG-Medicated Serum on Cell Proliferation

**Figure 13:**
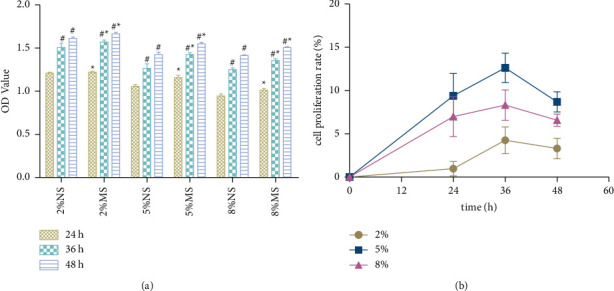
CKG-medicated serum treatment increased the proliferation of hBMSCs. (a) The CKG-medicated serum treatment for 24, 36, and 48 h significantly increased the proliferation of hBMSCs (*n* = 6). ^*∗*^*P* < 0.05, compared to the NS group of the same concentration; ^#^*P* < 0.05, compared to the last treatment time. (b) CKG-medicated serum increase hBMSCs proliferation rate, especially when treated with 5% MS for 36 h.

To detect the effect of CKG-medicated serum on hBMSCs' growth, we incubated cells in basic medium with different concentrations of NS and MS (2, 5, or 8%) for 24, 36, and 48 h. According to the results of the CCK-8 assay (Figure 13), the CKG-medicated serum treatment significantly improved hBMSCs growth at different time points. The cell proliferation rate was the highest when cells were treated with 5% MS for 36 h (*P* < 0.05).

#### 3.7.3. Western Blot

Comprehensively analyzing the screening results of the core targets and the KEGG enrichment analysis results, we can infer that CKG exerts its antiosteoporosis influence through multiple pathways. The top 3 pathways most closely related to OP in KEGG enrichment analysis are the MAPK signaling pathway, the PI3K-Akt signaling pathway, and the TNF signaling pathway. The top 5 core targets of CKG for treating OP are JUN, TNF, TP53, AKT1, and MAPK1. AKT plays an important role in the MAPK signaling pathway, the PI3K-Akt signaling pathway, and the TNF signaling pathway. AKT can negatively regulate B-Raf through phosphorylation of the B-Raf amino-terminal regulatory domain, which in turn affects the Ras/Raf pathway [28]. The inhibition of AKT leads to the enhancement of Ras/MAPK signaling [29]. TNF-*α* up-regulates several molecules such as the P2X7 receptor through the PI3K-Akt signaling pathway, the intracellular calcium concentration and secondary messengers, the entry of transcription factors into the nucleus, and the differentiation of bone marrow mesenchymal stem cells into osteoclasts [30]. Therefore, this study chose to observe the effect of CKG-medicated serum on the MAPK, PI3K-Akt, and TNF pathway-associated proteins in hBMSCs to verify that CKG participates in the MAPK signaling pathway, the PI3K-Akt signaling pathway, and the TNF signaling pathway by regulating AKT to play its part in treating OP.

**Figure 14:**
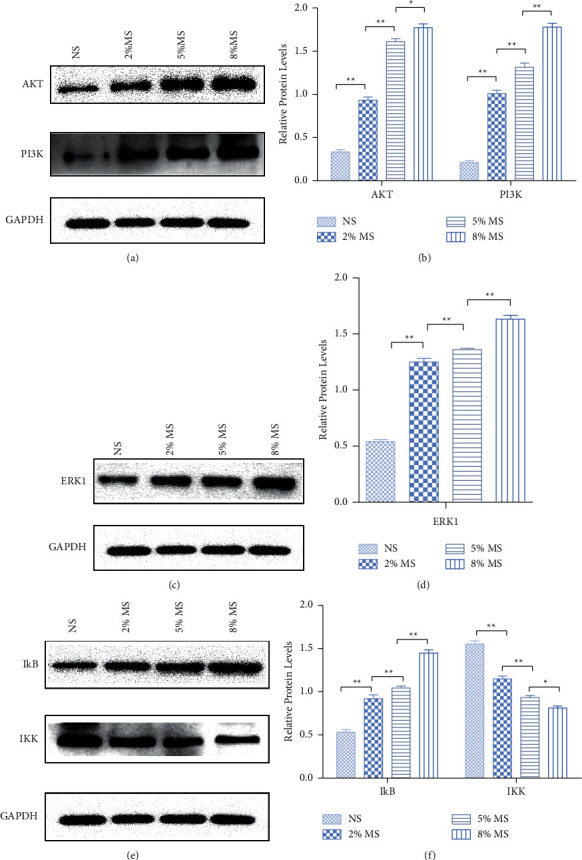
Effects of CKG-medicated serum on the expression of MAPK, PI3K-Akt, and TNF pathway-associated proteins. (a, c, e) Western blot was performed to detect AKT, PI3K, ERK1, IkB, and IKK expression levels in CKG-medicated serum-treated hBMSCs. (b, d, f) Quantification of the pathway-related proteins expression. (b) Quantification of the AKT and PI3K expression shown in (a) by normalizing to GAPDH. (d) Quantification of the ERK1 expression shown in C by normalizing to GAPDH. (f) Quantification of the IkB and IKK expression shown in (e) by normalizing to GAPDH. ^*∗*^*P* < 0.05 compared to the previous level group; ^*∗∗*^*P* < 0.01, compared to the previous level group.

Western blot results are shown in Figure 14. The histograms show quantitation of MAPK, PI3K-Akt, and TNF pathway-associated proteins in hBMSCs. Compared with NS, after 36 h of treatment, the AKT, PI3K, ERK1, and IkB protein expression levels in hBMSCs treated with 2, 5, and 8% MS increased. The gradual increase of AKT, PI3K, ERK1, and IkB protein expression levels was detected prominently by western blot as the CKG-MS concentration gradually increased. Compared with NS, after 36 h of treatment, the IKK protein expression levels in hBMSCs treated with 2, 5, and 8% MS decreased. The gradual decrease of IKK protein expression levels was detected prominently by western blot as the CKG-MS concentration gradually increased.

## 4. Discussion

OP is a metabolic disease caused by various factors, such as age, endocrine, viral infection, and various cytokines [31–33]. Given the multitarget and multipathway pharmacological properties of TCM, we think it is more effective in the treatment of OP. This characteristic of TCM may make it difficult to further study its intrinsic mechanisms. A network pharmacology approach combining systems biology and computer technology may provide a direction for the study of the complex mechanism of TCM [11]. In the present study, we used this approach to clarify the pharmacological mechanisms of OP alleviation by CKG.

In summary, this study analyzed the application of CKG in OP treatment by network pharmacology. We got 69 chemical ingredients, 250 targets of CKG, 1246 disease targets of OP, and 140 intersecting targets of CKG and OP. Topological analysis of the PPI network yielded 23 hub genes. GO enrichment analysis got 1723 entries, of which 1621 were BP entries, 20 were CC entries, and 82 were MF entries. The KEGG enrichment analysis yielded 180 entries. According to enrichment analysis and molecular docking technique, we inferred that the compounds of CKG (kaempferol, quercetin, 22,23-dihydrostigmasterol, luteolin, anhydroicaritin, 8-(3-methylbut-2-enyl)-2-phenyl-chromone, isorhamnetin, formononetin, calycosin, and 7-O-methylisomucronulatol) could exert antiosteoporosis effects by targeting multiple proteins (JUN, TNF, TP53, AKT1, and MAPK1) and signaling pathways (TNF signaling pathway, osteoclast differentiation, MAPK signaling pathway, PI3K-Akt signaling pathway, NF-kappa B signaling pathway, and Wnt signaling pathway).

The three bioactive compounds in CKG with the highest degree values were 22,23-dihydrostigmasterol, kaempferol, and quercetin, which were obtained from collections of multidatabase information and stringent screening conditions. According to relevant studies, 22,23-dihydrostigmasterol (beta-sitosterol) can play an important role in antiosteoporosis treatment by affecting osteoblasts' activity [34]. Kaempferol promotes bone formation in part via the mTOR signaling pathway [35]. Quercetin inhibits osteoblast apoptosis and inflammatory response, promotes osteogenesis, angiogenesis, and osteoclast apoptosis [36]. The top five core targets in order of degree value are JUN, TNF, TP53, AKT1, and MAPK1. Based on existing relevant literature and studies, JUN accelerates the growth and healing of bone in a drilled defect model [37]. TNF-*α* directly improves RANKL expression in osteoblasts and promotes osteoclast formation [38]. Related *in vivo* and *in vitro* experiments indicated that TP53 gene expression and serum P53 levels were upregulated in OP patients and OP mouse models; high p53 levels were associated with reduced bone mass, which could be partially reversed by knocking down p53 [39]. Restraint of AKTl can inhibit BMSC proliferation driven by HB-EGF [40]. MiR-186-5p can reduce IL-1*β*-induced inflammatory damage of chondrocytes by increasing MAPK1 [41]. Enrichment analysis results showed the primary pathways in the therapeutic process include viral-related signaling pathways, bacteria-related signaling pathways, IL-17 signaling pathway, TNF signaling pathway, osteoclast differentiation, MAPK signaling pathway, PI3K-Akt signaling pathway, NF-kappa B signaling pathway, and Wnt signaling pathway. The top 3 pathways most closely related to OP in KEGG enrichment analysis are the MAPK signaling pathway, the PI3K-Akt signaling pathway, and the TNF signaling pathway. PTH induces bone loss through the microbe-dependent expansion of intestinal TNF^+^ T cells and Th17 cells [42]. Inhibition of NF-kappa B and MAPK signaling can reduce RANKL-induced osteoclastogenesis [43]. The PI3K-Akt signaling pathway involves OP inhibition by promoting osteoblast proliferation, differentiation, and bone formation [44].

The results of molecular docking showed the core bioactive ingredients in CKG had good binding activity to the core targets for treating OP. Comprehensively analyzing the results of PPI, KEGG enrichment analysis, and molecular docking, we speculated that CKG contributes to OP treatment by engaging in multiple pathways. Analyzing the results of KEGG, we concluded that the three most important pathways are the MAPK signaling pathway, the PI3K-Akt signaling pathway, and the TNF signaling pathway. Through comprehensive analysis of the three pathways, we noticed that these pathways are all regulated by AKT, which can negatively regulate Raf, thereby affecting the MAPK signal pathway. AKT regulates the TNF pathway by phosphorylating IKK and is directly involved in the formation of the PI3K-Akt signal pathway. AKT is not only the core target screened by PPI but also the bridging protein between the top 3 pathways in the KEGG enrichment analysis. And related research studies have verified that regulating the AKT protein can affect these three pathways. Therefore, we speculate that CKG exerts an antiosteoporotic effect by affecting the expression of AKT. Then, we conducted related experiments. We used the CCK-8 method to observe the changes in cell proliferation rates after treating them with CKG-containing serum at different concentrations. Western blot was used to observe the changes of MAPK, PI3K-Akt, and TNF pathway-associated protein expression in cells treated with different concentrations of CKG-containing serum. The experimental results showed that CKG-containing serum can promote the proliferation of hBMSCs and that different concentrations of CKG-containing serum can increase the expression of AKT protein in hBMSCs. Therefore, we conclude that the antiosteoporosis effect of CKG may be achieved by regulating AKT and then engaging in the MAPK signaling pathway, the PI3K-Akt signaling pathway, and the TNF signaling pathway.

This study also has some limits, such as the accuracy and timeliness of database data, algorithms, and software characteristics of various analysis platforms. However, based on the completed research, the platforms and algorithms used in this study can meet the research needs. Besides, this study verified the mechanism of CKG in the OP treatment only through molecular docking and animal, cell, and protein experiments. But the results of this study can already indicate that CKG is involved in the main pathway of antiosteoporosis. Thus, we need to improve related experiments later to support the theory.

## 5. Conclusion

In this study, the method of combining network pharmacology and experimental confirmation was used to investigate the complexity of multicomponent, multitarget, and multipathway of CKG, which provides essential information for further understanding of the drug-target interaction, a basis for extracting practical drug ingredients from CKG for OP treatment, and a reference for future demonstration of the scientific truth of TCM.

## Figures and Tables

**Table 1:** The information of the bioactive compounds of CKG.

Abbreviation	Mol. ID	Molecule name	OB (%)	DL	Herb
COMMON1	MOL000359	Sitosterol	36.91	0.75	RA,RPA,HE
COMMON2	MOL000422	Kaempferol	41.88	0.24	RA,RPA,HE,CPM
COMMON3	MOL000211	Mairin	55.38	0.78	RA,RPA
COMMON4	MOL000098	Quercetin	46.43	0.28	RA,HE,RC,CPM
COMMON5	MOL000358	22,23-Dihydrostigmasterol	36.91	0.75	RPA,HE,RC
CPM1	MOL002259	Physcion diglucoside	41.65	0.63	CPM
RA1	MOL000033	(3S,8S,9S,10R,13R,14S,17R)-10,13-dimethyl-17-[(2R,5S)-5-propan-2-yloctan-2-yl]-2,3,4,7,8,9,11,12,14,15,16,17-dodecahydro-1H-cyclopenta[a]phenanthren-3-ol	36.23	0.78	RA
RA2	MOL000239	Jaranol	50.83	0.29	RA
RA3	MOL000296	Hederagenin	36.91	0.75	RA
RA4	MOL000354	Isorhamnetin	49.6	0.31	RA
RA5	MOL000371	3,9-di-O-methylnissolin	53.74	0.48	RA
RA6	MOL000378	7-O-methylisomucronulatol	74.69	0.3	RA
RA7	MOL000379	9,10-dimethoxypterocarpan-3-O-*β*-D-glucoside	36.74	0.92	RA
RA8	MOL000380	(6aR,11aR)-9,10-dimethoxy-6a,11a-dihydro-6H-benzofurano[3,2-c]chromen-3-ol	64.26	0.42	RA
RA9	MOL000387	Bifendate	31.1	0.67	RA
RA10	MOL000392	Formononetin	69.67	0.21	RA
RA11	MOL000417	Calycosin	47.75	0.24	RA
RA12	MOL000433	FA	68.96	0.71	RA
RA13	MOL000439	Isomucronulatol-7,2′-di-O-glucosiole	49.28	0.62	RA
RA14	MOL000442	1,7-dihydroxy-3,9-dimethoxy pterocarpene	39.05	0.48	RA
RA15	MOL002565	Calycosin-7-O-beta-D-glucopyranoside	41.6	0.81	RA
RA16	MOL009289	(−)-Medicarpin	49.22	0.34	RA
RA17	MOL000374	5′-hydroxyiso-muronulatol-2′,5′-di-O-glucoside	41.72	0.69	RA
RA18	MOL000398	Isoflavanone	109.99	0.3	RA
RA19	MOL000438	(3R)-3-(2-hydroxy-3,4-dimethoxyphenyl)chroman-7-ol	67.67	0.26	RA
RPA1	MOL001919	(3S,5R,8R,9R,10S,14S)-3,17-dihydroxy-4,4,8,10,14-pentamethyl-2,3,5,6,7,9-hexahydro-1H-cyclopenta[a]phenanthrene-15,16-dione	43.56	0.53	RPA
RPA2	MOL001924	Paeoniflorin	53.87	0.79	RPA
RPA3	MOL000492	(+)-Catechin	54.83	0.24	RPA
RPA4	MOL001918	Paeoniflorgenone	87.59	0.37	RPA
RPA5	MOL002710	Pyrethrin II	48.36	0.35	RPA
RPA6	MOL001930	Benzoyl paeoniflorin	31.27	0.75	RPA
RPA7	MOL001921	Lactiflorin	49.12	0.8	RPA
RPA8	MOL001910	11alpha,12alpha-epoxy-3beta-23-dihydroxy-30-norolean-20-en-28,12beta-olide	64.77	0.38	RPA
RPA9	MOL001928	Albiflorin_qt	66.64	0.33	RPA
RPA10	MOL001925	Paeoniflorin_qt	68.18	0.4	RPA
RPA11	MOL007014	8-debenzoylpaeonidanin	31.74	0.45	RPA
RPA12	MOL007025	Isobenzoylpaeoniflorin	31.14	0.54	RPA
HE1	MOL004427	Icariside A7	31.91	0.86	HE
HE2	MOL001792	DFV	32.76	0.18	HE
HE3	MOL003044	Chryseriol	35.85	0.27	HE
HE4	MOL000006	Luteolin	36.16	0.25	HE
HE5	MOL001771	Poriferast-5-en-3beta-ol	36.91	0.75	HE
HE6	MOL001510	24-epicampesterol	37.58	0.71	HE
HE7	MOL003542	8-Isopentenyl-kaempferol	38.04	0.39	HE
HE8	MOL004380	C-homoerythrinan, 1,6-didehydro-3,15,16-trimethoxy-, (3.beta.)-	39.14	0.49	HE
HE9	MOL004394	Anhydroicaritin-3-O-alpha-L-rhamnoside	41.58	0.61	HE
HE10	MOL004425	Icariin	41.58	0.61	HE
HE11	MOL001645	Linoleyl acetate	42.1	0.2	HE
HE12	MOL004373	Anhydroicaritin	45.41	0.44	HE
HE13	MOL004384	Yinyanghuo C	45.67	0.5	HE
HE14	MOL004391	8-(3-methylbut-2-enyl)-2-phenyl-chromone	48.54	0.25	HE
HE15	MOL004386	Yinyanghuo E	51.63	0.55	HE
HE16	MOL004396	1,2-bis(4-hydroxy-3-methoxyphenyl)propan-1,3-diol	52.31	0.22	HE
HE17	MOL004382	Yinyanghuo A	56.96	0.77	HE
HE18	MOL004388	6-hydroxy-11,12-dimethoxy-2,2-dimethyl-1,8-dioxo-2,3,4,8-tetrahydro-1H-isochromeno[3,4-h]isoquinolin-2-ium	60.64	0.66	HE
HE19	MOL004367	Olivil	62.23	0.41	HE
HE20	MOL000622	Magnograndiolide	63.71	0.19	HE
HE21	MOL002556	7-methoxy-8-(2′-hydroxy-3′-ethoxy-3′-methylbutyl)coumarin	40.36	0.21	HE
HE22	MOL008046	6-demethoxycapillarisin	52.33	0.25	HE
RC1	MOL012298	Rubrosterone	32.69	0.47	RC
RC2	MOL012286	Betavulgarin	68.75	0.39	RC
RC3	MOL012542	*β*-ecdysterone	44.23	0.82	RC
CO1	MOL010617	5,8,11,14,17-eicosapentaenoic acid	45.66	0.21	CO
CO2	MOL005320	Eicosanetetraenoic acid	45.57	0.2	CO
CO3	MOL010861	Vitamin D3	45.66	0.48	CO

**Table 2:** The abbreviations and degree values of the top 10 bioactive ingredients of the “C-T” network.

Abbreviation	Degree
COMMON4	431
COMMON2	170
COMMON5	51
HE4	46
RA10	27
RA6	23
RA4	22
HE12	20
HE14	20
RA11	16

**Table 3:** Top 5 hub genes of treating OP of CKG and topological values.

Name	Betweenness centrality	Closeness centrality	Degree
JUN	0.14728269	0.46551724	27
TP53	0.1435125	0.43724696	25
TNF	0.11585625	0.42687747	25
AKT1	0.10719742	0.41698842	22
MAPK1	0.07457652	0.421875	19

**Table 4:** Docking scores of the top 10 bioactive ingredients of CKG with 5 core targets.

Target	PDB ID	Compound	Affinity (kcal/mol)
JUN	2g01	8-(3-methylbut-2-enyl)-2-phenyl-chromone	−9.3
TP53	2j21	Luteolin	−7.8
TNF	2az5	Luteolin	−9
AKT1	1unq	22,23-Dihydrostigmasterol	−7.4
MAPK1	3w8q	Kaempferol	−9.4

## Data Availability

The data used to support the findings of this study are included within the article.

## Supplementary Materials

Supplementary Materials Supplementary Table 1: the abbreviations and degree values of bioactive ingredients of the “C-T” network. Supplementary Table 2: hub genes of treating OP of CKG. Supplementary Table 3: the results of GO enrichment analysis. Supplementary Table 4: the KEGG enrichment analysis results of the top 20 pathways with high correlation with OP. Supplementary File 5: the diagrams of the MAPK signaling pathway, PI3K-Akt signaling pathway, TNF signaling pathway, and the relationship diagram between them. Supplementary Table 6: docking scores of the top 10 bioactive ingredients of CKG with 5 core targets. Supplementary Table 7: the result of CCK-8. Supplementary Table 8: the results of KEGG enrichment analysis.
