# Supplementary material for: Study on the Mechanism of Compound Kidney-Invigorating Granule for Osteoporosis based on Network Pharmacology and Experimental Verification
Source: Evid Based Complement Alternat Med. 2022 Jan 4;2022:6453501. doi: 10.1155/2022/6453501 (PMC8752261; doi:10.1155/2022/6453501)
Supplement: Supplementary Materials — Supplementary Table 1: the abbreviations and degree values of bioactive ingredients of the “C-T” network. Supplementary Table 2: hub genes of treating OP of CKG. Supplementary Table 3: the results of GO enrichment analysis. Supplementary Table 4: the KEGG enrichment analysis results of the top 20 pathways with high correlation with OP. Supplementary File 5: the diagrams of the MAPK signaling pathway, PI3K-Akt signaling pathway, TNF signaling pathway, and the relationship diagram between them. Supplementary Table 6: docking scores of the top 10 bioactive ingredients of CKG with 5 core targets. Supplementary Table 7: the result of CCK-8. Supplementary Table 8: the results of KEGG enrichment analysis. [file 6453501.f1.zip › 6453501.f1/Supplementary File 5.docx]

The signal pathway diagrams of the MAPK signaling pathway, PI3K-Akt signaling pathway, and TNF signaling pathway were obtained via the KEGG analysis of the hub genes.


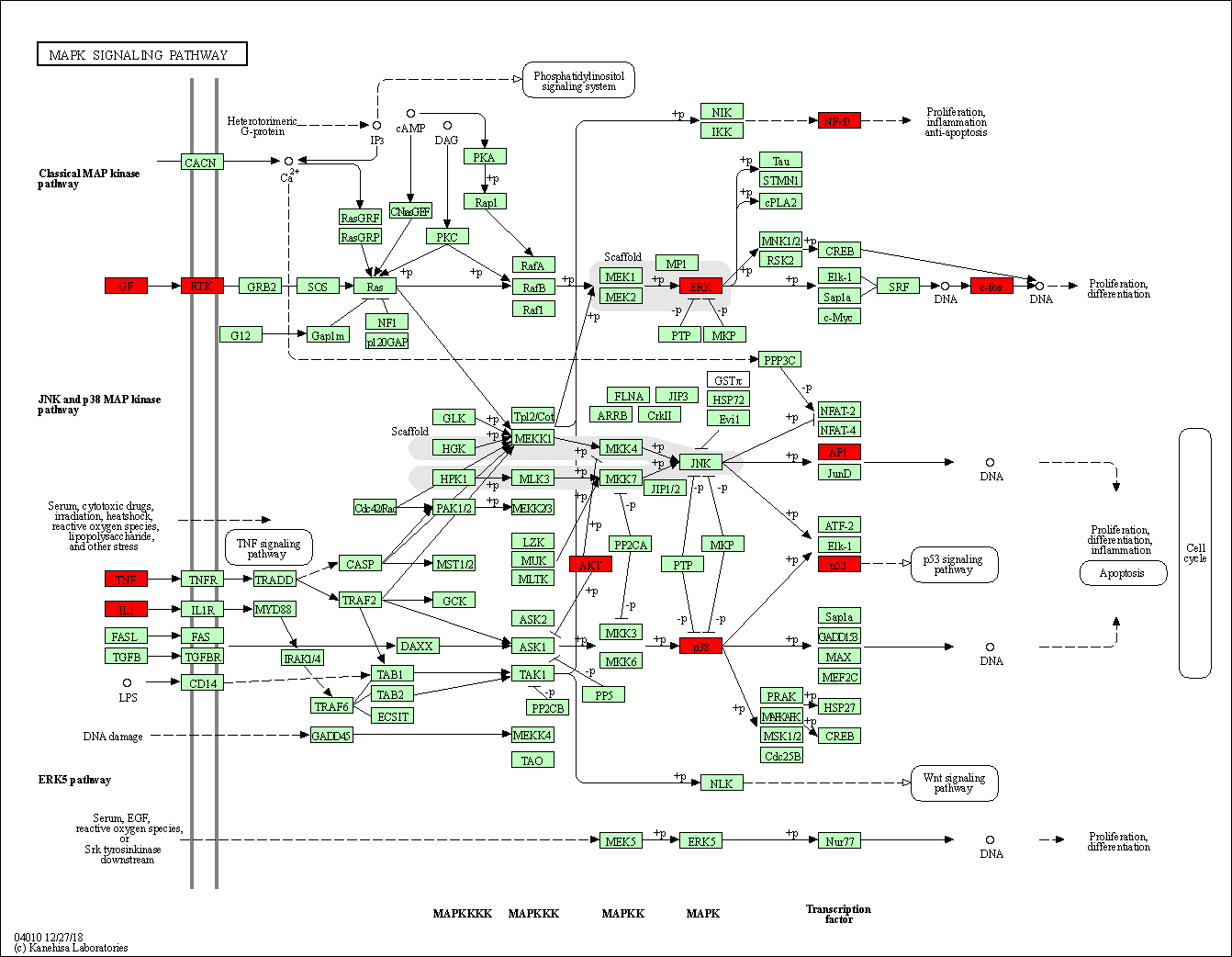


Figure 1. MAPK signaling pathway


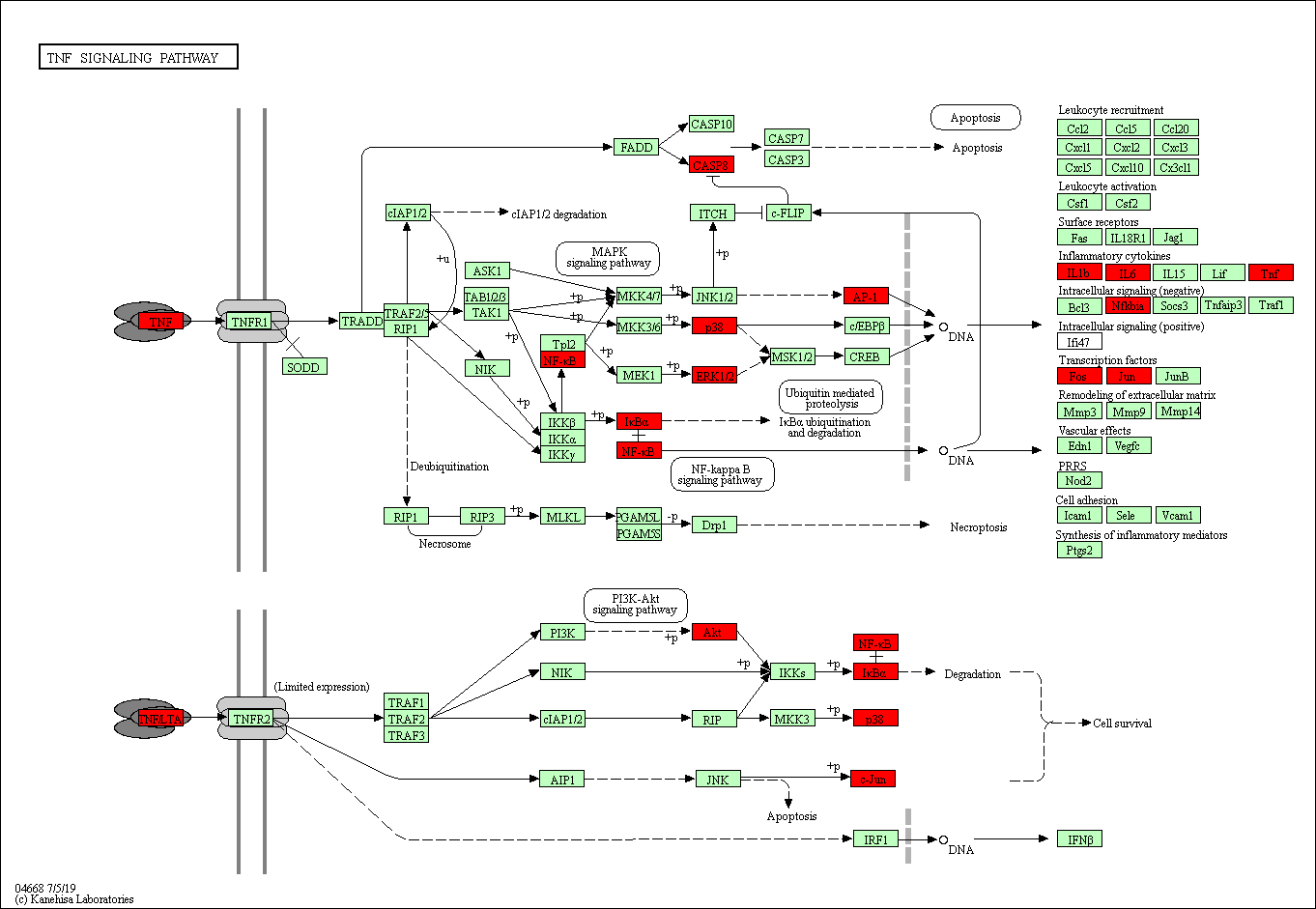


Figure 2 TNF signaling pathway


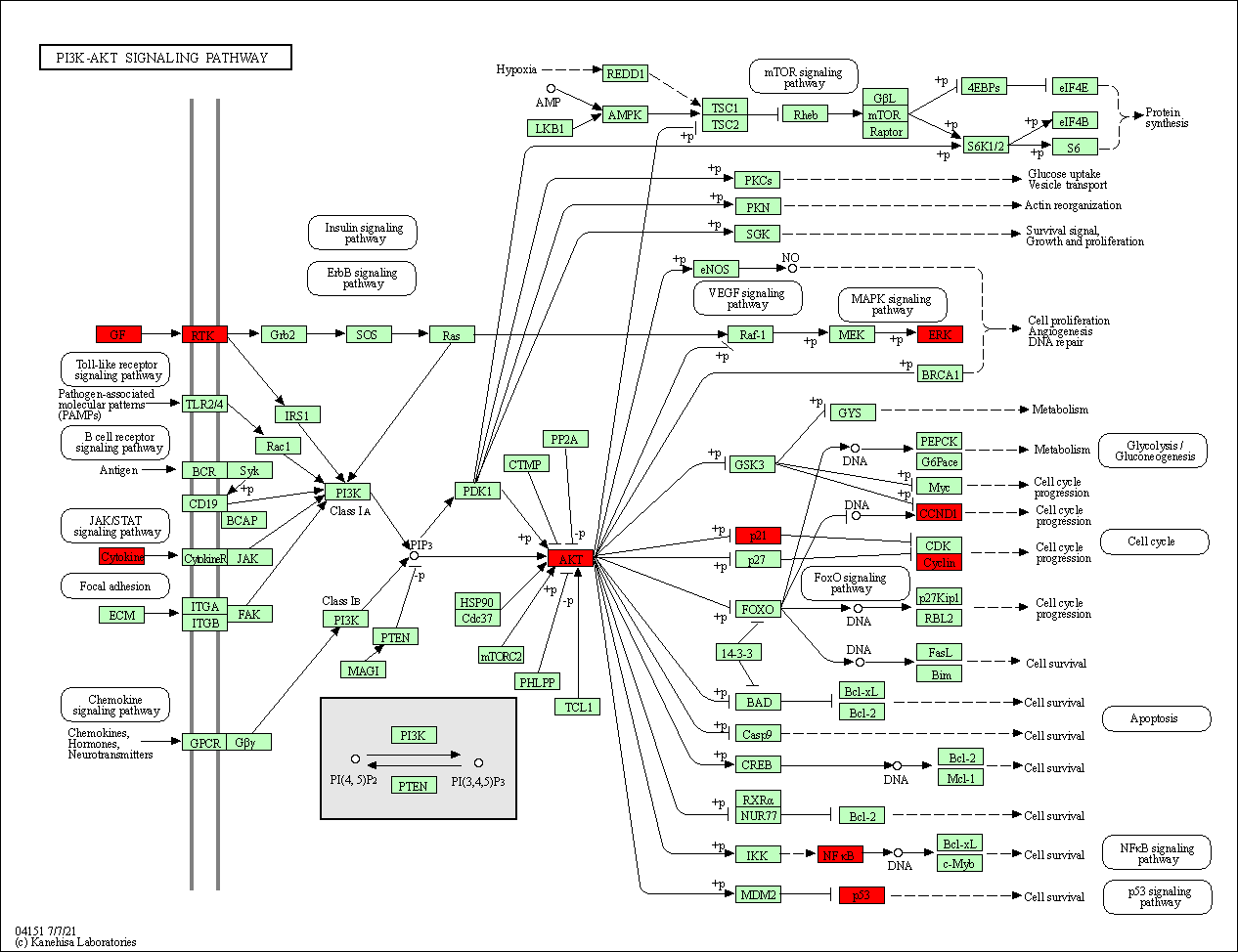


Figure 3. PI3K-Akt signaling pathway

*(All hub genes are marked in red)*

Comprehensive analysis of the three pathways, we can find that these pathways are all regulated by Akt. Akt can negatively regulate Raf, thereby affecting the Ras/Raf signaling pathway, Akt regulates NF-kB by phosphorylating IKK, and Akt is directly involved in the regulation of the PI3K/Akt signaling pathway. The relationship diagram is as follows.


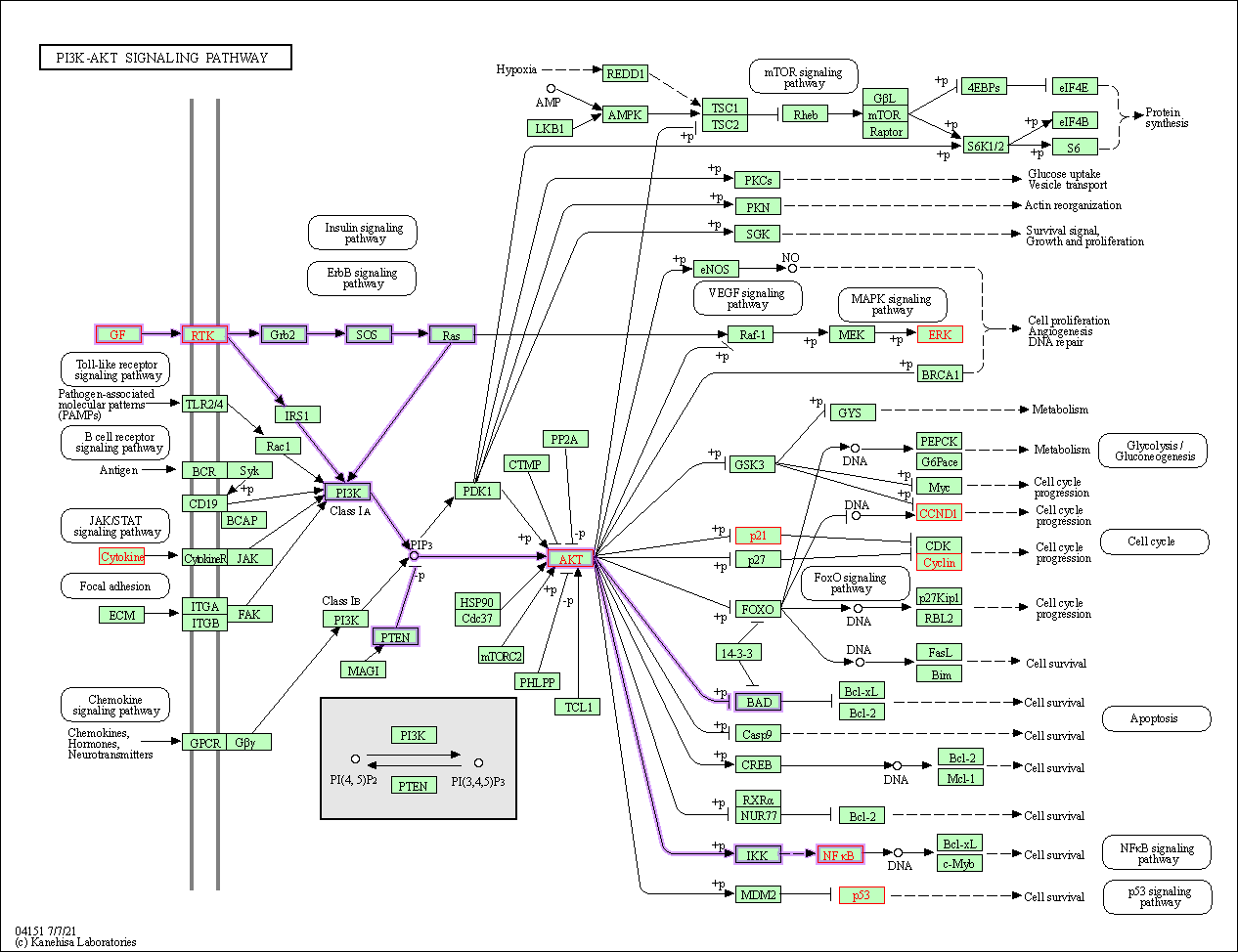


Figure 4

*( The purple marker is used to connect these three pathways, and the red font indicates the hub gene )*
