# Supplementary material for: Study on the Mechanism of Compound Kidney-Invigorating Granule for Osteoporosis based on Network Pharmacology and Experimental Verification
Source: Evid Based Complement Alternat Med. 2022 Jan 4;2022:6453501. doi: 10.1155/2022/6453501 (PMC8752261; doi:10.1155/2022/6453501)
Supplement: Supplementary Materials — Supplementary Table 1: the abbreviations and degree values of bioactive ingredients of the “C-T” network. Supplementary Table 2: hub genes of treating OP of CKG. Supplementary Table 3: the results of GO enrichment analysis. Supplementary Table 4: the KEGG enrichment analysis results of the top 20 pathways with high correlation with OP. Supplementary File 5: the diagrams of the MAPK signaling pathway, PI3K-Akt signaling pathway, TNF signaling pathway, and the relationship diagram between them. Supplementary Table 6: docking scores of the top 10 bioactive ingredients of CKG with 5 core targets. Supplementary Table 7: the result of CCK-8. Supplementary Table 8: the results of KEGG enrichment analysis. [file 6453501.f1.zip › 6453501.f1/Supplementary Table 1 .docx]

| Abbreviation | Degree |
| --- | --- |
| COMMON4 | 431 |
| COMMON2 | 170 |
| COMMON5 | 51 |
| HE4 | 46 |
| RA10 | 27 |
| RA6 | 23 |
| RA4 | 22 |
| HE12 | 20 |
| HE14 | 20 |
| RA11 | 16 |
| HE7 | 15 |
| RA16 | 15 |
| RC2 | 14 |
| HE3 | 13 |
| HE8 | 10 |
| RA5 | 10 |
| HE22 | 9 |
| RA8 | 9 |
| HE13 | 8 |
| RA2 | 8 |
| RPA3 | 8 |
| HE15 | 7 |
| HE21 | 7 |
| COMMON1 | 6 |
| HE16 | 6 |
| RA3 | 5 |
| HE11 | 4 |
| HE17 | 4 |
| RA9 | 4 |
| RPA2 | 4 |
| CO2 | 3 |
| HE2 | 3 |
| RA12 | 3 |
| RA15 | 3 |
| CO1 | 2 |
| CO3 | 2 |
| COMMON3 | 2 |
| HE1 | 2 |
| HE18 | 2 |
| HE19 | 2 |
| HE5 | 2 |
| HE6 | 2 |
| RA14 | 2 |
| RA7 | 2 |
| RPA5 | 2 |
| RA1 | 1 |
| RPA1 | 1 |
