# Supplementary material for: Study on the Mechanism of Compound Kidney-Invigorating Granule for Osteoporosis based on Network Pharmacology and Experimental Verification
Source: Evid Based Complement Alternat Med. 2022 Jan 4;2022:6453501. doi: 10.1155/2022/6453501 (PMC8752261; doi:10.1155/2022/6453501)
Supplement: Supplementary Materials — Supplementary Table 1: the abbreviations and degree values of bioactive ingredients of the “C-T” network. Supplementary Table 2: hub genes of treating OP of CKG. Supplementary Table 3: the results of GO enrichment analysis. Supplementary Table 4: the KEGG enrichment analysis results of the top 20 pathways with high correlation with OP. Supplementary File 5: the diagrams of the MAPK signaling pathway, PI3K-Akt signaling pathway, TNF signaling pathway, and the relationship diagram between them. Supplementary Table 6: docking scores of the top 10 bioactive ingredients of CKG with 5 core targets. Supplementary Table 7: the result of CCK-8. Supplementary Table 8: the results of KEGG enrichment analysis. [file 6453501.f1.zip › 6453501.f1/Supplementary Table 2 .docx]

| Hub gene | Degree |
| --- | --- |
| JUN | 18 |
| TNF | 12 |
| TP53 | 12 |
| MAPK1 | 12 |
| RELA | 12 |
| FOS | 10 |
| ESR1 | 9 |
| IL6 | 9 |
| MAPK14 | 9 |
| MYC | 9 |
| CCND1 | 8 |
| IL1B | 7 |
| CXCL8 | 7 |
| AKT1 | 7 |
| VEGFA | 6 |
| CDKN1A | 6 |
| STAT1 | 6 |
| RB1 | 6 |
| CASP8 | 5 |
| PPARG | 5 |
| NFKBIA | 5 |
| EGFR | 4 |
| NOS3 | 4 |
