# Supplementary material for: Study on the Mechanism of Compound Kidney-Invigorating Granule for Osteoporosis based on Network Pharmacology and Experimental Verification
Source: Evid Based Complement Alternat Med. 2022 Jan 4;2022:6453501. doi: 10.1155/2022/6453501 (PMC8752261; doi:10.1155/2022/6453501)
Supplement: Supplementary Materials — Supplementary Table 1: the abbreviations and degree values of bioactive ingredients of the “C-T” network. Supplementary Table 2: hub genes of treating OP of CKG. Supplementary Table 3: the results of GO enrichment analysis. Supplementary Table 4: the KEGG enrichment analysis results of the top 20 pathways with high correlation with OP. Supplementary File 5: the diagrams of the MAPK signaling pathway, PI3K-Akt signaling pathway, TNF signaling pathway, and the relationship diagram between them. Supplementary Table 6: docking scores of the top 10 bioactive ingredients of CKG with 5 core targets. Supplementary Table 7: the result of CCK-8. Supplementary Table 8: the results of KEGG enrichment analysis. [file 6453501.f1.zip › 6453501.f1/Supplementary Table 3 .docx]

| ONTOLOGY | ID | Description | pvalue | p.adjust | qvalue |
| --- | --- | --- | --- | --- | --- |
| BP | GO:0051090 | regulation of DNA-binding transcription factor activity | 6.92E-18 | 1.57E-14 | 4.42E-15 |
| BP | GO:0032496 | response to lipopolysaccharide | 1.27E-17 | 1.57E-14 | 4.42E-15 |
| BP | GO:0002237 | response to molecule of bacterial origin | 2.10E-17 | 1.73E-14 | 4.88E-15 |
| BP | GO:0009612 | response to mechanical stimulus | 3.37E-16 | 2.08E-13 | 5.87E-14 |
| BP | GO:0071216 | cellular response to biotic stimulus | 1.23E-15 | 6.10E-13 | 1.72E-13 |
| BP | GO:0071222 | cellular response to lipopolysaccharide | 2.06E-14 | 8.11E-12 | 2.29E-12 |
| BP | GO:0050673 | epithelial cell proliferation | 2.30E-14 | 8.11E-12 | 2.29E-12 |
| BP | GO:0071219 | cellular response to molecule of bacterial origin | 2.90E-14 | 8.95E-12 | 2.52E-12 |
| BP | GO:0000302 | response to reactive oxygen species | 7.18E-14 | 1.97E-11 | 5.56E-12 |
| BP | GO:0033002 | muscle cell proliferation | 9.68E-14 | 2.39E-11 | 6.74E-12 |
| BP | GO:0034614 | cellular response to reactive oxygen species | 2.29E-13 | 5.14E-11 | 1.45E-11 |
| BP | GO:0031663 | lipopolysaccharide-mediated signaling pathway | 4.53E-13 | 9.33E-11 | 2.63E-11 |
| BP | GO:0030099 | myeloid cell differentiation | 6.27E-13 | 1.19E-10 | 3.36E-11 |
| BP | GO:0034599 | cellular response to oxidative stress | 1.00E-12 | 1.77E-10 | 5.00E-11 |
| BP | GO:0002573 | myeloid leukocyte differentiation | 1.33E-12 | 2.19E-10 | 6.19E-11 |
| BP | GO:0006979 | response to oxidative stress | 1.51E-12 | 2.33E-10 | 6.57E-11 |
| BP | GO:0071496 | cellular response to external stimulus | 3.17E-12 | 4.61E-10 | 1.30E-10 |
| BP | GO:0031667 | response to nutrient levels | 4.51E-12 | 6.19E-10 | 1.75E-10 |
| BP | GO:0048145 | regulation of fibroblast proliferation | 6.13E-12 | 7.98E-10 | 2.25E-10 |
| BP | GO:0048144 | fibroblast proliferation | 6.69E-12 | 8.27E-10 | 2.33E-10 |
| BP | GO:0045637 | regulation of myeloid cell differentiation | 8.62E-12 | 1.01E-09 | 2.86E-10 |
| BP | GO:0050678 | regulation of epithelial cell proliferation | 9.31E-12 | 1.05E-09 | 2.95E-10 |
| BP | GO:0048545 | response to steroid hormone | 1.12E-11 | 1.20E-09 | 3.38E-10 |
| BP | GO:0045639 | positive regulation of myeloid cell differentiation | 1.19E-11 | 1.22E-09 | 3.45E-10 |
| BP | GO:0035994 | response to muscle stretch | 1.51E-11 | 1.49E-09 | 4.21E-10 |
| BP | GO:0048660 | regulation of smooth muscle cell proliferation | 1.67E-11 | 1.58E-09 | 4.47E-10 |
| BP | GO:0048659 | smooth muscle cell proliferation | 1.83E-11 | 1.68E-09 | 4.73E-10 |
| BP | GO:2000379 | positive regulation of reactive oxygen species metabolic process | 2.69E-11 | 2.37E-09 | 6.69E-10 |
| BP | GO:0071356 | cellular response to tumor necrosis factor | 3.24E-11 | 2.76E-09 | 7.78E-10 |
| BP | GO:0048608 | reproductive structure development | 3.39E-11 | 2.79E-09 | 7.87E-10 |
| BP | GO:0061458 | reproductive system development | 3.63E-11 | 2.89E-09 | 8.16E-10 |
| BP | GO:0009314 | response to radiation | 4.96E-11 | 3.83E-09 | 1.08E-09 |
| BP | GO:0034612 | response to tumor necrosis factor | 6.03E-11 | 4.52E-09 | 1.27E-09 |
| BP | GO:0050727 | regulation of inflammatory response | 1.08E-10 | 7.84E-09 | 2.21E-09 |
| BP | GO:0010001 | glial cell differentiation | 1.28E-10 | 9.05E-09 | 2.55E-09 |
| BP | GO:0009411 | response to UV | 2.67E-10 | 1.84E-08 | 5.17E-09 |
| BP | GO:0009266 | response to temperature stimulus | 3.04E-10 | 2.03E-08 | 5.73E-09 |
| BP | GO:0051091 | positive regulation of DNA-binding transcription factor activity | 5.36E-10 | 3.49E-08 | 9.83E-09 |
| BP | GO:0007249 | I-kappaB kinase/NF-kappaB signaling | 6.81E-10 | 4.31E-08 | 1.22E-08 |
| BP | GO:0071276 | cellular response to cadmium ion | 7.57E-10 | 4.68E-08 | 1.32E-08 |
| BP | GO:0051052 | regulation of DNA metabolic process | 1.01E-09 | 6.09E-08 | 1.72E-08 |
| BP | GO:0072593 | reactive oxygen species metabolic process | 1.05E-09 | 6.15E-08 | 1.73E-08 |
| BP | GO:0051403 | stress-activated MAPK cascade | 1.10E-09 | 6.28E-08 | 1.77E-08 |
| BP | GO:0048732 | gland development | 1.12E-09 | 6.28E-08 | 1.77E-08 |
| BP | GO:0042063 | gliogenesis | 1.23E-09 | 6.77E-08 | 1.91E-08 |
| BP | GO:1903708 | positive regulation of hemopoiesis | 1.79E-09 | 9.65E-08 | 2.72E-08 |
| BP | GO:0048661 | positive regulation of smooth muscle cell proliferation | 2.02E-09 | 1.06E-07 | 3.00E-08 |
| BP | GO:0009416 | response to light stimulus | 2.31E-09 | 1.17E-07 | 3.30E-08 |
| BP | GO:0031098 | stress-activated protein kinase signaling cascade | 2.36E-09 | 1.17E-07 | 3.30E-08 |
| BP | GO:1901654 | response to ketone | 2.41E-09 | 1.17E-07 | 3.30E-08 |
| BP | GO:2001237 | negative regulation of extrinsic apoptotic signaling pathway | 2.41E-09 | 1.17E-07 | 3.30E-08 |
| BP | GO:1903706 | regulation of hemopoiesis | 2.47E-09 | 1.17E-07 | 3.30E-08 |
| BP | GO:2000377 | regulation of reactive oxygen species metabolic process | 2.59E-09 | 1.21E-07 | 3.41E-08 |
| BP | GO:0061614 | pri-miRNA transcription by RNA polymerase II | 2.64E-09 | 1.21E-07 | 3.41E-08 |
| BP | GO:0007568 | aging | 2.74E-09 | 1.23E-07 | 3.47E-08 |
| BP | GO:0032103 | positive regulation of response to external stimulus | 2.88E-09 | 1.27E-07 | 3.58E-08 |
| BP | GO:1903829 | positive regulation of cellular protein localization | 2.95E-09 | 1.28E-07 | 3.61E-08 |
| BP | GO:0071214 | cellular response to abiotic stimulus | 3.49E-09 | 1.46E-07 | 4.12E-08 |
| BP | GO:0104004 | cellular response to environmental stimulus | 3.49E-09 | 1.46E-07 | 4.12E-08 |
| BP | GO:0071902 | positive regulation of protein serine/threonine kinase activity | 3.74E-09 | 1.54E-07 | 4.35E-08 |
| BP | GO:0048146 | positive regulation of fibroblast proliferation | 4.03E-09 | 1.61E-07 | 4.53E-08 |
| BP | GO:0050999 | regulation of nitric-oxide synthase activity | 4.03E-09 | 1.61E-07 | 4.53E-08 |
| BP | GO:0002761 | regulation of myeloid leukocyte differentiation | 4.92E-09 | 1.93E-07 | 5.45E-08 |
| BP | GO:0002763 | positive regulation of myeloid leukocyte differentiation | 5.41E-09 | 2.09E-07 | 5.90E-08 |
| BP | GO:0010888 | negative regulation of lipid storage | 6.70E-09 | 2.55E-07 | 7.18E-08 |
| BP | GO:0061900 | glial cell activation | 7.82E-09 | 2.93E-07 | 8.26E-08 |
| BP | GO:2001234 | negative regulation of apoptotic signaling pathway | 8.15E-09 | 2.97E-07 | 8.37E-08 |
| BP | GO:0035690 | cellular response to drug | 8.17E-09 | 2.97E-07 | 8.37E-08 |
| BP | GO:0032872 | regulation of stress-activated MAPK cascade | 1.00E-08 | 3.53E-07 | 9.94E-08 |
| BP | GO:0043122 | regulation of I-kappaB kinase/NF-kappaB signaling | 1.00E-08 | 3.53E-07 | 9.94E-08 |
| BP | GO:1905953 | negative regulation of lipid localization | 1.01E-08 | 3.53E-07 | 9.94E-08 |
| BP | GO:0070302 | regulation of stress-activated protein kinase signaling cascade | 1.06E-08 | 3.65E-07 | 1.03E-07 |
| BP | GO:0046686 | response to cadmium ion | 1.19E-08 | 4.05E-07 | 1.14E-07 |
| BP | GO:0032768 | regulation of monooxygenase activity | 1.29E-08 | 4.27E-07 | 1.20E-07 |
| BP | GO:0048872 | homeostasis of number of cells | 1.30E-08 | 4.27E-07 | 1.20E-07 |
| BP | GO:0006606 | protein import into nucleus | 1.65E-08 | 5.36E-07 | 1.51E-07 |
| BP | GO:2001233 | regulation of apoptotic signaling pathway | 1.72E-08 | 5.52E-07 | 1.56E-07 |
| BP | GO:0019915 | lipid storage | 1.76E-08 | 5.59E-07 | 1.58E-07 |
| BP | GO:0051384 | response to glucocorticoid | 1.87E-08 | 5.84E-07 | 1.65E-07 |
| BP | GO:0014074 | response to purine-containing compound | 2.11E-08 | 6.51E-07 | 1.84E-07 |
| BP | GO:0001890 | placenta development | 2.37E-08 | 7.25E-07 | 2.04E-07 |
| BP | GO:0048568 | embryonic organ development | 2.59E-08 | 7.81E-07 | 2.20E-07 |
| BP | GO:2001236 | regulation of extrinsic apoptotic signaling pathway | 2.67E-08 | 7.95E-07 | 2.24E-07 |
| BP | GO:0014015 | positive regulation of gliogenesis | 2.71E-08 | 7.99E-07 | 2.25E-07 |
| BP | GO:1905952 | regulation of lipid localization | 2.88E-08 | 8.35E-07 | 2.35E-07 |
| BP | GO:0150076 | neuroinflammatory response | 2.91E-08 | 8.35E-07 | 2.35E-07 |
| BP | GO:0048143 | astrocyte activation | 3.01E-08 | 8.56E-07 | 2.41E-07 |
| BP | GO:0000082 | G1/S transition of mitotic cell cycle | 3.08E-08 | 8.66E-07 | 2.44E-07 |
| BP | GO:0031960 | response to corticosteroid | 3.47E-08 | 9.65E-07 | 2.72E-07 |
| BP | GO:0051170 | import into nucleus | 3.60E-08 | 9.90E-07 | 2.79E-07 |
| BP | GO:1904951 | positive regulation of establishment of protein localization | 4.23E-08 | 1.15E-06 | 3.24E-07 |
| BP | GO:0048708 | astrocyte differentiation | 4.29E-08 | 1.15E-06 | 3.25E-07 |
| BP | GO:0044843 | cell cycle G1/S phase transition | 4.84E-08 | 1.27E-06 | 3.59E-07 |
| BP | GO:0090068 | positive regulation of cell cycle process | 4.84E-08 | 1.27E-06 | 3.59E-07 |
| BP | GO:0018105 | peptidyl-serine phosphorylation | 4.96E-08 | 1.29E-06 | 3.64E-07 |
| BP | GO:1902895 | positive regulation of pri-miRNA transcription by RNA polymerase II | 5.39E-08 | 1.39E-06 | 3.91E-07 |
| BP | GO:0001936 | regulation of endothelial cell proliferation | 5.69E-08 | 1.45E-06 | 4.09E-07 |
| BP | GO:0009895 | negative regulation of catabolic process | 6.07E-08 | 1.53E-06 | 4.32E-07 |
| BP | GO:0038061 | NIK/NF-kappaB signaling | 7.18E-08 | 1.79E-06 | 5.05E-07 |
| BP | GO:2000045 | regulation of G1/S transition of mitotic cell cycle | 7.42E-08 | 1.83E-06 | 5.17E-07 |
| BP | GO:0018209 | peptidyl-serine modification | 8.23E-08 | 2.01E-06 | 5.68E-07 |
| BP | GO:1904031 | positive regulation of cyclin-dependent protein kinase activity | 8.93E-08 | 2.16E-06 | 6.10E-07 |
| BP | GO:1901216 | positive regulation of neuron death | 9.10E-08 | 2.17E-06 | 6.12E-07 |
| BP | GO:0046677 | response to antibiotic | 9.14E-08 | 2.17E-06 | 6.12E-07 |
| BP | GO:0001935 | endothelial cell proliferation | 9.26E-08 | 2.18E-06 | 6.14E-07 |
| BP | GO:0007219 | Notch signaling pathway | 9.85E-08 | 2.30E-06 | 6.47E-07 |
| BP | GO:0030224 | monocyte differentiation | 1.00E-07 | 2.30E-06 | 6.48E-07 |
| BP | GO:1903131 | mononuclear cell differentiation | 1.00E-07 | 2.30E-06 | 6.48E-07 |
| BP | GO:0032147 | activation of protein kinase activity | 1.03E-07 | 2.35E-06 | 6.61E-07 |
| BP | GO:0002685 | regulation of leukocyte migration | 1.08E-07 | 2.42E-06 | 6.83E-07 |
| BP | GO:0017038 | protein import | 1.18E-07 | 2.63E-06 | 7.41E-07 |
| BP | GO:0006913 | nucleocytoplasmic transport | 1.26E-07 | 2.79E-06 | 7.87E-07 |
| BP | GO:1902806 | regulation of cell cycle G1/S phase transition | 1.29E-07 | 2.82E-06 | 7.95E-07 |
| BP | GO:0051169 | nuclear transport | 1.34E-07 | 2.91E-06 | 8.20E-07 |
| BP | GO:0070997 | neuron death | 1.40E-07 | 3.00E-06 | 8.46E-07 |
| BP | GO:0050679 | positive regulation of epithelial cell proliferation | 1.45E-07 | 3.09E-06 | 8.70E-07 |
| BP | GO:0033138 | positive regulation of peptidyl-serine phosphorylation | 1.59E-07 | 3.35E-06 | 9.45E-07 |
| BP | GO:1902893 | regulation of pri-miRNA transcription by RNA polymerase II | 1.72E-07 | 3.60E-06 | 1.02E-06 |
| BP | GO:0051341 | regulation of oxidoreductase activity | 1.74E-07 | 3.62E-06 | 1.02E-06 |
| BP | GO:2000278 | regulation of DNA biosynthetic process | 1.83E-07 | 3.76E-06 | 1.06E-06 |
| BP | GO:0045862 | positive regulation of proteolysis | 1.86E-07 | 3.78E-06 | 1.06E-06 |
| BP | GO:0043281 | regulation of cysteine-type endopeptidase activity involved in apoptotic process | 1.86E-07 | 3.78E-06 | 1.06E-06 |
| BP | GO:0010038 | response to metal ion | 1.89E-07 | 3.81E-06 | 1.07E-06 |
| BP | GO:0007584 | response to nutrient | 2.08E-07 | 4.14E-06 | 1.17E-06 |
| BP | GO:0001701 | in utero embryonic development | 2.23E-07 | 4.42E-06 | 1.25E-06 |
| BP | GO:0014002 | astrocyte development | 2.30E-07 | 4.50E-06 | 1.27E-06 |
| BP | GO:0045444 | fat cell differentiation | 2.31E-07 | 4.50E-06 | 1.27E-06 |
| BP | GO:0097191 | extrinsic apoptotic signaling pathway | 2.37E-07 | 4.59E-06 | 1.29E-06 |
| BP | GO:0021782 | glial cell development | 2.61E-07 | 5.01E-06 | 1.41E-06 |
| BP | GO:0051054 | positive regulation of DNA metabolic process | 2.64E-07 | 5.01E-06 | 1.41E-06 |
| BP | GO:0045765 | regulation of angiogenesis | 2.67E-07 | 5.04E-06 | 1.42E-06 |
| BP | GO:1901222 | regulation of NIK/NF-kappaB signaling | 2.73E-07 | 5.11E-06 | 1.44E-06 |
| BP | GO:0010883 | regulation of lipid storage | 2.76E-07 | 5.13E-06 | 1.45E-06 |
| BP | GO:0045787 | positive regulation of cell cycle | 2.97E-07 | 5.48E-06 | 1.54E-06 |
| BP | GO:0097305 | response to alcohol | 2.99E-07 | 5.48E-06 | 1.55E-06 |
| BP | GO:0051972 | regulation of telomerase activity | 3.28E-07 | 5.97E-06 | 1.68E-06 |
| BP | GO:0007050 | cell cycle arrest | 3.31E-07 | 5.97E-06 | 1.68E-06 |
| BP | GO:1905477 | positive regulation of protein localization to membrane | 3.36E-07 | 6.02E-06 | 1.70E-06 |
| BP | GO:2000116 | regulation of cysteine-type endopeptidase activity | 3.48E-07 | 6.18E-06 | 1.74E-06 |
| BP | GO:0014013 | regulation of gliogenesis | 3.64E-07 | 6.43E-06 | 1.81E-06 |
| BP | GO:0022407 | regulation of cell-cell adhesion | 3.77E-07 | 6.61E-06 | 1.86E-06 |
| BP | GO:0009409 | response to cold | 3.88E-07 | 6.76E-06 | 1.90E-06 |
| BP | GO:0002687 | positive regulation of leukocyte migration | 4.27E-07 | 7.38E-06 | 2.08E-06 |
| BP | GO:0006352 | DNA-templated transcription, initiation | 4.42E-07 | 7.59E-06 | 2.14E-06 |
| BP | GO:1901550 | regulation of endothelial cell development | 4.63E-07 | 7.84E-06 | 2.21E-06 |
| BP | GO:1903140 | regulation of establishment of endothelial barrier | 4.63E-07 | 7.84E-06 | 2.21E-06 |
| BP | GO:0001558 | regulation of cell growth | 4.67E-07 | 7.85E-06 | 2.21E-06 |
| BP | GO:0051222 | positive regulation of protein transport | 4.82E-07 | 8.06E-06 | 2.27E-06 |
| BP | GO:0045598 | regulation of fat cell differentiation | 4.97E-07 | 8.25E-06 | 2.33E-06 |
| BP | GO:1901342 | regulation of vasculature development | 5.14E-07 | 8.47E-06 | 2.39E-06 |
| BP | GO:0046683 | response to organophosphorus | 5.36E-07 | 8.72E-06 | 2.46E-06 |
| BP | GO:0048565 | digestive tract development | 5.36E-07 | 8.72E-06 | 2.46E-06 |
| BP | GO:0043406 | positive regulation of MAP kinase activity | 5.45E-07 | 8.80E-06 | 2.48E-06 |
| BP | GO:0034504 | protein localization to nucleus | 5.96E-07 | 9.57E-06 | 2.70E-06 |
| BP | GO:0033135 | regulation of peptidyl-serine phosphorylation | 6.43E-07 | 1.03E-05 | 2.89E-06 |
| BP | GO:0060249 | anatomical structure homeostasis | 6.50E-07 | 1.03E-05 | 2.90E-06 |
| BP | GO:0031668 | cellular response to extracellular stimulus | 6.80E-07 | 1.07E-05 | 3.02E-06 |
| BP | GO:0030730 | sequestering of triglyceride | 7.36E-07 | 1.14E-05 | 3.21E-06 |
| BP | GO:0045073 | regulation of chemokine biosynthetic process | 7.36E-07 | 1.14E-05 | 3.21E-06 |
| BP | GO:0030879 | mammary gland development | 7.40E-07 | 1.14E-05 | 3.21E-06 |
| BP | GO:1902105 | regulation of leukocyte differentiation | 7.42E-07 | 1.14E-05 | 3.21E-06 |
| BP | GO:1902107 | positive regulation of leukocyte differentiation | 7.66E-07 | 1.17E-05 | 3.29E-06 |
| BP | GO:0052547 | regulation of peptidase activity | 8.16E-07 | 1.24E-05 | 3.48E-06 |
| BP | GO:0055123 | digestive system development | 8.20E-07 | 1.24E-05 | 3.48E-06 |
| BP | GO:0060485 | mesenchyme development | 8.43E-07 | 1.26E-05 | 3.54E-06 |
| BP | GO:0002262 | myeloid cell homeostasis | 8.48E-07 | 1.26E-05 | 3.54E-06 |
| BP | GO:0010212 | response to ionizing radiation | 8.48E-07 | 1.26E-05 | 3.54E-06 |
| BP | GO:0030522 | intracellular receptor signaling pathway | 8.79E-07 | 1.29E-05 | 3.64E-06 |
| BP | GO:0042033 | chemokine biosynthetic process | 9.05E-07 | 1.31E-05 | 3.69E-06 |
| BP | GO:0050755 | chemokine metabolic process | 9.05E-07 | 1.31E-05 | 3.69E-06 |
| BP | GO:0060965 | negative regulation of gene silencing by miRNA | 9.05E-07 | 1.31E-05 | 3.69E-06 |
| BP | GO:0046824 | positive regulation of nucleocytoplasmic transport | 9.31E-07 | 1.34E-05 | 3.77E-06 |
| BP | GO:0001819 | positive regulation of cytokine production | 9.72E-07 | 1.38E-05 | 3.90E-06 |
| BP | GO:0051348 | negative regulation of transferase activity | 9.74E-07 | 1.38E-05 | 3.90E-06 |
| BP | GO:0050729 | positive regulation of inflammatory response | 1.03E-06 | 1.45E-05 | 4.09E-06 |
| BP | GO:0062013 | positive regulation of small molecule metabolic process | 1.03E-06 | 1.45E-05 | 4.09E-06 |
| BP | GO:0030856 | regulation of epithelial cell differentiation | 1.14E-06 | 1.59E-05 | 4.48E-06 |
| BP | GO:0048511 | rhythmic process | 1.19E-06 | 1.65E-05 | 4.66E-06 |
| BP | GO:0042108 | positive regulation of cytokine biosynthetic process | 1.27E-06 | 1.76E-05 | 4.96E-06 |
| BP | GO:0016049 | cell growth | 1.29E-06 | 1.77E-05 | 4.99E-06 |
| BP | GO:0048871 | multicellular organismal homeostasis | 1.31E-06 | 1.77E-05 | 4.99E-06 |
| BP | GO:0060749 | mammary gland alveolus development | 1.32E-06 | 1.77E-05 | 4.99E-06 |
| BP | GO:0061377 | mammary gland lobule development | 1.32E-06 | 1.77E-05 | 4.99E-06 |
| BP | GO:0150078 | positive regulation of neuroinflammatory response | 1.32E-06 | 1.77E-05 | 4.99E-06 |
| BP | GO:0035924 | cellular response to vascular endothelial growth factor stimulus | 1.35E-06 | 1.81E-05 | 5.09E-06 |
| BP | GO:0045931 | positive regulation of mitotic cell cycle | 1.41E-06 | 1.88E-05 | 5.30E-06 |
| BP | GO:0060149 | negative regulation of posttranscriptional gene silencing | 1.56E-06 | 2.03E-05 | 5.73E-06 |
| BP | GO:0060252 | positive regulation of glial cell proliferation | 1.56E-06 | 2.03E-05 | 5.73E-06 |
| BP | GO:0060967 | negative regulation of gene silencing by RNA | 1.56E-06 | 2.03E-05 | 5.73E-06 |
| BP | GO:1903798 | regulation of production of miRNAs involved in gene silencing by miRNA | 1.56E-06 | 2.03E-05 | 5.73E-06 |
| BP | GO:0050900 | leukocyte migration | 1.58E-06 | 2.04E-05 | 5.76E-06 |
| BP | GO:0033209 | tumor necrosis factor-mediated signaling pathway | 1.59E-06 | 2.05E-05 | 5.78E-06 |
| BP | GO:1901214 | regulation of neuron death | 1.68E-06 | 2.15E-05 | 6.07E-06 |
| BP | GO:0061180 | mammary gland epithelium development | 1.70E-06 | 2.17E-05 | 6.12E-06 |
| BP | GO:0031100 | animal organ regeneration | 1.80E-06 | 2.27E-05 | 6.40E-06 |
| BP | GO:0043627 | response to estrogen | 1.80E-06 | 2.27E-05 | 6.40E-06 |
| BP | GO:0070371 | ERK1 and ERK2 cascade | 1.81E-06 | 2.27E-05 | 6.40E-06 |
| BP | GO:0032495 | response to muramyl dipeptide | 1.84E-06 | 2.29E-05 | 6.45E-06 |
| BP | GO:0030324 | lung development | 1.84E-06 | 2.29E-05 | 6.45E-06 |
| BP | GO:2001252 | positive regulation of chromosome organization | 1.95E-06 | 2.41E-05 | 6.79E-06 |
| BP | GO:0030323 | respiratory tube development | 2.06E-06 | 2.54E-05 | 7.15E-06 |
| BP | GO:0001959 | regulation of cytokine-mediated signaling pathway | 2.12E-06 | 2.58E-05 | 7.28E-06 |
| BP | GO:0009615 | response to virus | 2.13E-06 | 2.58E-05 | 7.28E-06 |
| BP | GO:0051412 | response to corticosterone | 2.14E-06 | 2.58E-05 | 7.28E-06 |
| BP | GO:0070920 | regulation of production of small RNA involved in gene silencing by RNA | 2.14E-06 | 2.58E-05 | 7.28E-06 |
| BP | GO:0006809 | nitric oxide biosynthetic process | 2.23E-06 | 2.66E-05 | 7.51E-06 |
| BP | GO:1901224 | positive regulation of NIK/NF-kappaB signaling | 2.23E-06 | 2.66E-05 | 7.51E-06 |
| BP | GO:0071347 | cellular response to interleukin-1 | 2.24E-06 | 2.66E-05 | 7.51E-06 |
| BP | GO:0051235 | maintenance of location | 2.28E-06 | 2.70E-05 | 7.61E-06 |
| BP | GO:0010827 | regulation of glucose transmembrane transport | 2.35E-06 | 2.77E-05 | 7.80E-06 |
| BP | GO:0071260 | cellular response to mechanical stimulus | 2.47E-06 | 2.89E-05 | 8.14E-06 |
| BP | GO:1901522 | positive regulation of transcription from RNA polymerase II promoter involved in cellular response to chemical stimulus | 2.48E-06 | 2.89E-05 | 8.14E-06 |
| BP | GO:0043123 | positive regulation of I-kappaB kinase/NF-kappaB signaling | 2.50E-06 | 2.90E-05 | 8.17E-06 |
| BP | GO:0043405 | regulation of MAP kinase activity | 2.58E-06 | 2.98E-05 | 8.39E-06 |
| BP | GO:0045930 | negative regulation of mitotic cell cycle | 2.62E-06 | 3.01E-05 | 8.50E-06 |
| BP | GO:1905475 | regulation of protein localization to membrane | 2.78E-06 | 3.18E-05 | 8.96E-06 |
| BP | GO:0033044 | regulation of chromosome organization | 2.81E-06 | 3.20E-05 | 9.01E-06 |
| BP | GO:0050995 | negative regulation of lipid catabolic process | 2.85E-06 | 3.21E-05 | 9.04E-06 |
| BP | GO:0006367 | transcription initiation from RNA polymerase II promoter | 2.85E-06 | 3.21E-05 | 9.04E-06 |
| BP | GO:0001101 | response to acid chemical | 2.85E-06 | 3.21E-05 | 9.04E-06 |
| BP | GO:0046209 | nitric oxide metabolic process | 2.87E-06 | 3.21E-05 | 9.06E-06 |
| BP | GO:0060759 | regulation of response to cytokine stimulus | 3.00E-06 | 3.33E-05 | 9.38E-06 |
| BP | GO:0071248 | cellular response to metal ion | 3.00E-06 | 3.33E-05 | 9.38E-06 |
| BP | GO:0042594 | response to starvation | 3.08E-06 | 3.40E-05 | 9.58E-06 |
| BP | GO:0007565 | female pregnancy | 3.16E-06 | 3.46E-05 | 9.75E-06 |
| BP | GO:0002718 | regulation of cytokine production involved in immune response | 3.16E-06 | 3.46E-05 | 9.75E-06 |
| BP | GO:1904705 | regulation of vascular smooth muscle cell proliferation | 3.32E-06 | 3.58E-05 | 1.01E-05 |
| BP | GO:1990874 | vascular smooth muscle cell proliferation | 3.32E-06 | 3.58E-05 | 1.01E-05 |
| BP | GO:2001057 | reactive nitrogen species metabolic process | 3.32E-06 | 3.58E-05 | 1.01E-05 |
| BP | GO:0071897 | DNA biosynthetic process | 3.50E-06 | 3.76E-05 | 1.06E-05 |
| BP | GO:0010952 | positive regulation of peptidase activity | 3.58E-06 | 3.84E-05 | 1.08E-05 |
| BP | GO:0001892 | embryonic placenta development | 3.64E-06 | 3.86E-05 | 1.09E-05 |
| BP | GO:0002690 | positive regulation of leukocyte chemotaxis | 3.64E-06 | 3.86E-05 | 1.09E-05 |
| BP | GO:0031099 | regeneration | 3.67E-06 | 3.87E-05 | 1.09E-05 |
| BP | GO:0060541 | respiratory system development | 3.67E-06 | 3.87E-05 | 1.09E-05 |
| BP | GO:0002053 | positive regulation of mesenchymal cell proliferation | 3.69E-06 | 3.87E-05 | 1.09E-05 |
| BP | GO:0097194 | execution phase of apoptosis | 3.81E-06 | 3.97E-05 | 1.12E-05 |
| BP | GO:1901992 | positive regulation of mitotic cell cycle phase transition | 4.36E-06 | 4.52E-05 | 1.28E-05 |
| BP | GO:0002064 | epithelial cell development | 4.57E-06 | 4.70E-05 | 1.33E-05 |
| BP | GO:0070555 | response to interleukin-1 | 4.57E-06 | 4.70E-05 | 1.33E-05 |
| BP | GO:2000144 | positive regulation of DNA-templated transcription, initiation | 4.69E-06 | 4.81E-05 | 1.35E-05 |
| BP | GO:0033273 | response to vitamin | 4.75E-06 | 4.85E-05 | 1.37E-05 |
| BP | GO:0071229 | cellular response to acid chemical | 4.79E-06 | 4.87E-05 | 1.37E-05 |
| BP | GO:0097529 | myeloid leukocyte migration | 4.90E-06 | 4.96E-05 | 1.40E-05 |
| BP | GO:0032770 | positive regulation of monooxygenase activity | 5.24E-06 | 5.27E-05 | 1.49E-05 |
| BP | GO:0033598 | mammary gland epithelial cell proliferation | 5.24E-06 | 5.27E-05 | 1.49E-05 |
| BP | GO:1901653 | cellular response to peptide | 5.55E-06 | 5.55E-05 | 1.56E-05 |
| BP | GO:0000075 | cell cycle checkpoint | 5.62E-06 | 5.56E-05 | 1.57E-05 |
| BP | GO:0032869 | cellular response to insulin stimulus | 5.62E-06 | 5.56E-05 | 1.57E-05 |
| BP | GO:0051591 | response to cAMP | 5.62E-06 | 5.56E-05 | 1.57E-05 |
| BP | GO:0071241 | cellular response to inorganic substance | 5.75E-06 | 5.66E-05 | 1.60E-05 |
| BP | GO:0007263 | nitric oxide mediated signal transduction | 5.84E-06 | 5.73E-05 | 1.62E-05 |
| BP | GO:1903039 | positive regulation of leukocyte cell-cell adhesion | 5.88E-06 | 5.74E-05 | 1.62E-05 |
| BP | GO:0070482 | response to oxygen levels | 6.33E-06 | 6.15E-05 | 1.73E-05 |
| BP | GO:0070498 | interleukin-1-mediated signaling pathway | 6.34E-06 | 6.15E-05 | 1.73E-05 |
| BP | GO:0044706 | multi-multicellular organism process | 6.42E-06 | 6.20E-05 | 1.75E-05 |
| BP | GO:0048147 | negative regulation of fibroblast proliferation | 6.49E-06 | 6.24E-05 | 1.76E-05 |
| BP | GO:0030595 | leukocyte chemotaxis | 6.71E-06 | 6.40E-05 | 1.81E-05 |
| BP | GO:0001503 | ossification | 6.71E-06 | 6.40E-05 | 1.81E-05 |
| BP | GO:0000079 | regulation of cyclin-dependent protein serine/threonine kinase activity | 6.86E-06 | 6.50E-05 | 1.83E-05 |
| BP | GO:0002367 | cytokine production involved in immune response | 6.86E-06 | 6.50E-05 | 1.83E-05 |
| BP | GO:0010876 | lipid localization | 6.90E-06 | 6.51E-05 | 1.84E-05 |
| BP | GO:0001894 | tissue homeostasis | 7.16E-06 | 6.67E-05 | 1.88E-05 |
| BP | GO:0002675 | positive regulation of acute inflammatory response | 7.18E-06 | 6.67E-05 | 1.88E-05 |
| BP | GO:0010165 | response to X-ray | 7.18E-06 | 6.67E-05 | 1.88E-05 |
| BP | GO:0045737 | positive regulation of cyclin-dependent protein serine/threonine kinase activity | 7.18E-06 | 6.67E-05 | 1.88E-05 |
| BP | GO:0045785 | positive regulation of cell adhesion | 7.21E-06 | 6.67E-05 | 1.88E-05 |
| BP | GO:0046822 | regulation of nucleocytoplasmic transport | 7.42E-06 | 6.84E-05 | 1.93E-05 |
| BP | GO:0008593 | regulation of Notch signaling pathway | 7.70E-06 | 7.08E-05 | 2.00E-05 |
| BP | GO:0051385 | response to mineralocorticoid | 7.91E-06 | 7.24E-05 | 2.04E-05 |
| BP | GO:0042136 | neurotransmitter biosynthetic process | 8.00E-06 | 7.24E-05 | 2.04E-05 |
| BP | GO:1901989 | positive regulation of cell cycle phase transition | 8.00E-06 | 7.24E-05 | 2.04E-05 |
| BP | GO:1904029 | regulation of cyclin-dependent protein kinase activity | 8.00E-06 | 7.24E-05 | 2.04E-05 |
| BP | GO:0071453 | cellular response to oxygen levels | 8.29E-06 | 7.48E-05 | 2.11E-05 |
| BP | GO:1904659 | glucose transmembrane transport | 8.61E-06 | 7.74E-05 | 2.18E-05 |
| BP | GO:0010464 | regulation of mesenchymal cell proliferation | 8.70E-06 | 7.79E-05 | 2.20E-05 |
| BP | GO:0031669 | cellular response to nutrient levels | 8.82E-06 | 7.87E-05 | 2.22E-05 |
| BP | GO:0052548 | regulation of endopeptidase activity | 9.76E-06 | 8.68E-05 | 2.45E-05 |
| BP | GO:0008645 | hexose transmembrane transport | 9.95E-06 | 8.82E-05 | 2.49E-05 |
| BP | GO:0001933 | negative regulation of protein phosphorylation | 1.03E-05 | 9.07E-05 | 2.56E-05 |
| BP | GO:0060964 | regulation of gene silencing by miRNA | 1.03E-05 | 9.07E-05 | 2.56E-05 |
| BP | GO:0060251 | regulation of glial cell proliferation | 1.04E-05 | 9.10E-05 | 2.57E-05 |
| BP | GO:0070423 | nucleotide-binding oligomerization domain containing signaling pathway | 1.04E-05 | 9.10E-05 | 2.57E-05 |
| BP | GO:0002688 | regulation of leukocyte chemotaxis | 1.07E-05 | 9.19E-05 | 2.59E-05 |
| BP | GO:0015749 | monosaccharide transmembrane transport | 1.07E-05 | 9.19E-05 | 2.59E-05 |
| BP | GO:0030218 | erythrocyte differentiation | 1.07E-05 | 9.19E-05 | 2.59E-05 |
| BP | GO:0042035 | regulation of cytokine biosynthetic process | 1.07E-05 | 9.19E-05 | 2.59E-05 |
| BP | GO:0010742 | macrophage derived foam cell differentiation | 1.14E-05 | 9.61E-05 | 2.71E-05 |
| BP | GO:0035872 | nucleotide-binding domain, leucine rich repeat containing receptor signaling pathway | 1.14E-05 | 9.61E-05 | 2.71E-05 |
| BP | GO:0090077 | foam cell differentiation | 1.14E-05 | 9.61E-05 | 2.71E-05 |
| BP | GO:1901099 | negative regulation of signal transduction in absence of ligand | 1.14E-05 | 9.61E-05 | 2.71E-05 |
| BP | GO:2001240 | negative regulation of extrinsic apoptotic signaling pathway in absence of ligand | 1.14E-05 | 9.61E-05 | 2.71E-05 |
| BP | GO:0034219 | carbohydrate transmembrane transport | 1.14E-05 | 9.65E-05 | 2.72E-05 |
| BP | GO:0060147 | regulation of posttranscriptional gene silencing | 1.18E-05 | 9.91E-05 | 2.79E-05 |
| BP | GO:0060966 | regulation of gene silencing by RNA | 1.18E-05 | 9.91E-05 | 2.79E-05 |
| BP | GO:2000027 | regulation of animal organ morphogenesis | 1.21E-05 | 0.000101098 | 2.85E-05 |
| BP | GO:2000142 | regulation of DNA-templated transcription, initiation | 1.23E-05 | 0.000102767 | 2.90E-05 |
| BP | GO:1901990 | regulation of mitotic cell cycle phase transition | 1.25E-05 | 0.000103854 | 2.93E-05 |
| BP | GO:0022409 | positive regulation of cell-cell adhesion | 1.26E-05 | 0.000103967 | 2.93E-05 |
| BP | GO:0050730 | regulation of peptidyl-tyrosine phosphorylation | 1.28E-05 | 0.000105599 | 2.98E-05 |
| BP | GO:0045088 | regulation of innate immune response | 1.39E-05 | 0.000113793 | 3.21E-05 |
| BP | GO:0034101 | erythrocyte homeostasis | 1.40E-05 | 0.000113882 | 3.21E-05 |
| BP | GO:1903409 | reactive oxygen species biosynthetic process | 1.40E-05 | 0.000113882 | 3.21E-05 |
| BP | GO:0042089 | cytokine biosynthetic process | 1.44E-05 | 0.000117228 | 3.30E-05 |
| BP | GO:0060969 | negative regulation of gene silencing | 1.45E-05 | 0.000117514 | 3.31E-05 |
| BP | GO:0008637 | apoptotic mitochondrial changes | 1.49E-05 | 0.000119465 | 3.37E-05 |
| BP | GO:0042107 | cytokine metabolic process | 1.49E-05 | 0.000119465 | 3.37E-05 |
| BP | GO:0051101 | regulation of DNA binding | 1.49E-05 | 0.000119465 | 3.37E-05 |
| BP | GO:0062012 | regulation of small molecule metabolic process | 1.51E-05 | 0.000120944 | 3.41E-05 |
| BP | GO:2000134 | negative regulation of G1/S transition of mitotic cell cycle | 1.54E-05 | 0.000122518 | 3.45E-05 |
| BP | GO:0031334 | positive regulation of protein complex assembly | 1.60E-05 | 0.000126665 | 3.57E-05 |
| BP | GO:0050714 | positive regulation of protein secretion | 1.60E-05 | 0.000126665 | 3.57E-05 |
| BP | GO:0043491 | protein kinase B signaling | 1.63E-05 | 0.000128366 | 3.62E-05 |
| BP | GO:0032368 | regulation of lipid transport | 1.64E-05 | 0.000128366 | 3.62E-05 |
| BP | GO:0043620 | regulation of DNA-templated transcription in response to stress | 1.64E-05 | 0.000128366 | 3.62E-05 |
| BP | GO:0045927 | positive regulation of growth | 1.66E-05 | 0.000129624 | 3.65E-05 |
| BP | GO:0043254 | regulation of protein complex assembly | 1.67E-05 | 0.000130018 | 3.67E-05 |
| BP | GO:0042326 | negative regulation of phosphorylation | 1.69E-05 | 0.000130436 | 3.68E-05 |
| BP | GO:0061028 | establishment of endothelial barrier | 1.69E-05 | 0.000130436 | 3.68E-05 |
| BP | GO:0150077 | regulation of neuroinflammatory response | 1.69E-05 | 0.000130436 | 3.68E-05 |
| BP | GO:0032868 | response to insulin | 1.72E-05 | 0.000132224 | 3.73E-05 |
| BP | GO:0050769 | positive regulation of neurogenesis | 1.81E-05 | 0.000139244 | 3.93E-05 |
| BP | GO:1902807 | negative regulation of cell cycle G1/S phase transition | 1.85E-05 | 0.000141465 | 3.99E-05 |
| BP | GO:0043280 | positive regulation of cysteine-type endopeptidase activity involved in apoptotic process | 1.90E-05 | 0.000145317 | 4.10E-05 |
| BP | GO:1901987 | regulation of cell cycle phase transition | 1.95E-05 | 0.000147535 | 4.16E-05 |
| BP | GO:0045429 | positive regulation of nitric oxide biosynthetic process | 1.95E-05 | 0.000147535 | 4.16E-05 |
| BP | GO:0045746 | negative regulation of Notch signaling pathway | 1.95E-05 | 0.000147535 | 4.16E-05 |
| BP | GO:0032355 | response to estradiol | 2.02E-05 | 0.000152301 | 4.29E-05 |
| BP | GO:0001889 | liver development | 2.08E-05 | 0.000154425 | 4.35E-05 |
| BP | GO:0050921 | positive regulation of chemotaxis | 2.08E-05 | 0.000154425 | 4.35E-05 |
| BP | GO:0010463 | mesenchymal cell proliferation | 2.09E-05 | 0.000154425 | 4.35E-05 |
| BP | GO:0030225 | macrophage differentiation | 2.09E-05 | 0.000154425 | 4.35E-05 |
| BP | GO:0045601 | regulation of endothelial cell differentiation | 2.09E-05 | 0.000154425 | 4.35E-05 |
| BP | GO:0070266 | necroptotic process | 2.09E-05 | 0.000154425 | 4.35E-05 |
| BP | GO:1904407 | positive regulation of nitric oxide metabolic process | 2.09E-05 | 0.000154425 | 4.35E-05 |
| BP | GO:0002793 | positive regulation of peptide secretion | 2.26E-05 | 0.000166292 | 4.69E-05 |
| BP | GO:0061008 | hepaticobiliary system development | 2.27E-05 | 0.000166418 | 4.69E-05 |
| BP | GO:0002700 | regulation of production of molecular mediator of immune response | 2.33E-05 | 0.000170703 | 4.81E-05 |
| BP | GO:0010632 | regulation of epithelial cell migration | 2.38E-05 | 0.000173098 | 4.88E-05 |
| BP | GO:0035196 | production of miRNAs involved in gene silencing by miRNA | 2.39E-05 | 0.000173098 | 4.88E-05 |
| BP | GO:0060711 | labyrinthine layer development | 2.39E-05 | 0.000173098 | 4.88E-05 |
| BP | GO:0071354 | cellular response to interleukin-6 | 2.39E-05 | 0.000173098 | 4.88E-05 |
| BP | GO:0006953 | acute-phase response | 2.56E-05 | 0.000183145 | 5.16E-05 |
| BP | GO:0070849 | response to epidermal growth factor | 2.56E-05 | 0.000183145 | 5.16E-05 |
| BP | GO:2001239 | regulation of extrinsic apoptotic signaling pathway in absence of ligand | 2.56E-05 | 0.000183145 | 5.16E-05 |
| BP | GO:0050890 | cognition | 2.58E-05 | 0.000184175 | 5.19E-05 |
| BP | GO:0001774 | microglial cell activation | 2.72E-05 | 0.000193515 | 5.46E-05 |
| BP | GO:0002269 | leukocyte activation involved in inflammatory response | 2.72E-05 | 0.000193515 | 5.46E-05 |
| BP | GO:0000077 | DNA damage checkpoint | 2.76E-05 | 0.00019461 | 5.49E-05 |
| BP | GO:0071901 | negative regulation of protein serine/threonine kinase activity | 2.76E-05 | 0.00019461 | 5.49E-05 |
| BP | GO:0042542 | response to hydrogen peroxide | 2.83E-05 | 0.000198793 | 5.60E-05 |
| BP | GO:0045834 | positive regulation of lipid metabolic process | 2.83E-05 | 0.000198793 | 5.60E-05 |
| BP | GO:0030857 | negative regulation of epithelial cell differentiation | 2.90E-05 | 0.000200778 | 5.66E-05 |
| BP | GO:0035094 | response to nicotine | 2.90E-05 | 0.000200778 | 5.66E-05 |
| BP | GO:0043124 | negative regulation of I-kappaB kinase/NF-kappaB signaling | 2.90E-05 | 0.000200778 | 5.66E-05 |
| BP | GO:0051205 | protein insertion into membrane | 2.90E-05 | 0.000200778 | 5.66E-05 |
| BP | GO:0097300 | programmed necrotic cell death | 2.90E-05 | 0.000200778 | 5.66E-05 |
| BP | GO:0060326 | cell chemotaxis | 2.93E-05 | 0.000201698 | 5.69E-05 |
| BP | GO:1903037 | regulation of leukocyte cell-cell adhesion | 2.93E-05 | 0.000201698 | 5.69E-05 |
| BP | GO:0008643 | carbohydrate transport | 2.99E-05 | 0.000203895 | 5.75E-05 |
| BP | GO:0014065 | phosphatidylinositol 3-kinase signaling | 2.99E-05 | 0.000203895 | 5.75E-05 |
| BP | GO:0051592 | response to calcium ion | 2.99E-05 | 0.000203895 | 5.75E-05 |
| BP | GO:0009267 | cellular response to starvation | 3.07E-05 | 0.000207606 | 5.85E-05 |
| BP | GO:2001056 | positive regulation of cysteine-type endopeptidase activity | 3.07E-05 | 0.000207606 | 5.85E-05 |
| BP | GO:0014009 | glial cell proliferation | 3.08E-05 | 0.000207606 | 5.85E-05 |
| BP | GO:0070741 | response to interleukin-6 | 3.08E-05 | 0.000207606 | 5.85E-05 |
| BP | GO:1900087 | positive regulation of G1/S transition of mitotic cell cycle | 3.08E-05 | 0.000207606 | 5.85E-05 |
| BP | GO:0048754 | branching morphogenesis of an epithelial tube | 3.15E-05 | 0.000211405 | 5.96E-05 |
| BP | GO:0031050 | dsRNA processing | 3.27E-05 | 0.000218618 | 6.16E-05 |
| BP | GO:0070918 | production of small RNA involved in gene silencing by RNA | 3.27E-05 | 0.000218618 | 6.16E-05 |
| BP | GO:0000187 | activation of MAPK activity | 3.31E-05 | 0.000220863 | 6.23E-05 |
| BP | GO:0042133 | neurotransmitter metabolic process | 3.40E-05 | 0.000224789 | 6.34E-05 |
| BP | GO:0045807 | positive regulation of endocytosis | 3.40E-05 | 0.000224789 | 6.34E-05 |
| BP | GO:0090316 | positive regulation of intracellular protein transport | 3.40E-05 | 0.000224789 | 6.34E-05 |
| BP | GO:0060968 | regulation of gene silencing | 3.58E-05 | 0.00023588 | 6.65E-05 |
| BP | GO:0043535 | regulation of blood vessel endothelial cell migration | 3.67E-05 | 0.000240969 | 6.79E-05 |
| BP | GO:0038066 | p38MAPK cascade | 3.67E-05 | 0.000240969 | 6.79E-05 |
| BP | GO:0031570 | DNA integrity checkpoint | 3.76E-05 | 0.000246043 | 6.94E-05 |
| BP | GO:0071375 | cellular response to peptide hormone stimulus | 3.80E-05 | 0.0002478 | 6.99E-05 |
| BP | GO:0002720 | positive regulation of cytokine production involved in immune response | 3.89E-05 | 0.000251602 | 7.09E-05 |
| BP | GO:0050994 | regulation of lipid catabolic process | 3.89E-05 | 0.000251602 | 7.09E-05 |
| BP | GO:0051353 | positive regulation of oxidoreductase activity | 3.89E-05 | 0.000251602 | 7.09E-05 |
| BP | GO:0002673 | regulation of acute inflammatory response | 3.95E-05 | 0.000255151 | 7.19E-05 |
| BP | GO:0010332 | response to gamma radiation | 4.34E-05 | 0.000277102 | 7.81E-05 |
| BP | GO:0060688 | regulation of morphogenesis of a branching structure | 4.34E-05 | 0.000277102 | 7.81E-05 |
| BP | GO:0090183 | regulation of kidney development | 4.34E-05 | 0.000277102 | 7.81E-05 |
| BP | GO:1903078 | positive regulation of protein localization to plasma membrane | 4.34E-05 | 0.000277102 | 7.81E-05 |
| BP | GO:0007093 | mitotic cell cycle checkpoint | 4.57E-05 | 0.000289272 | 8.15E-05 |
| BP | GO:0050680 | negative regulation of epithelial cell proliferation | 4.57E-05 | 0.000289272 | 8.15E-05 |
| BP | GO:0043525 | positive regulation of neuron apoptotic process | 4.58E-05 | 0.000289272 | 8.15E-05 |
| BP | GO:1903428 | positive regulation of reactive oxygen species biosynthetic process | 4.58E-05 | 0.000289272 | 8.15E-05 |
| BP | GO:0007159 | leukocyte cell-cell adhesion | 4.79E-05 | 0.000302065 | 8.52E-05 |
| BP | GO:0032722 | positive regulation of chemokine production | 4.82E-05 | 0.000303254 | 8.55E-05 |
| BP | GO:0038095 | Fc-epsilon receptor signaling pathway | 5.02E-05 | 0.000314701 | 8.87E-05 |
| BP | GO:0001836 | release of cytochrome c from mitochondria | 5.08E-05 | 0.000316026 | 8.91E-05 |
| BP | GO:0001885 | endothelial cell development | 5.08E-05 | 0.000316026 | 8.91E-05 |
| BP | GO:0033619 | membrane protein proteolysis | 5.08E-05 | 0.000316026 | 8.91E-05 |
| BP | GO:0010634 | positive regulation of epithelial cell migration | 5.25E-05 | 0.000326155 | 9.19E-05 |
| BP | GO:0010721 | negative regulation of cell development | 5.28E-05 | 0.000327234 | 9.22E-05 |
| BP | GO:0046324 | regulation of glucose import | 5.34E-05 | 0.000329077 | 9.28E-05 |
| BP | GO:1902808 | positive regulation of cell cycle G1/S phase transition | 5.34E-05 | 0.000329077 | 9.28E-05 |
| BP | GO:0001659 | temperature homeostasis | 5.49E-05 | 0.000336791 | 9.49E-05 |
| BP | GO:0003018 | vascular process in circulatory system | 5.49E-05 | 0.000336791 | 9.49E-05 |
| BP | GO:0048638 | regulation of developmental growth | 5.50E-05 | 0.000336791 | 9.49E-05 |
| BP | GO:0010631 | epithelial cell migration | 5.81E-05 | 0.000354741 | 1.00E-04 |
| BP | GO:0009408 | response to heat | 5.87E-05 | 0.000356882 | 0.000100603 |
| BP | GO:0002753 | cytoplasmic pattern recognition receptor signaling pathway | 5.89E-05 | 0.000356882 | 0.000100603 |
| BP | GO:0070265 | necrotic cell death | 5.89E-05 | 0.000356882 | 0.000100603 |
| BP | GO:0048469 | cell maturation | 6.01E-05 | 0.000362985 | 0.000102323 |
| BP | GO:0090132 | epithelium migration | 6.05E-05 | 0.000364849 | 0.000102849 |
| BP | GO:0010950 | positive regulation of endopeptidase activity | 6.14E-05 | 0.00036811 | 0.000103768 |
| BP | GO:0031571 | mitotic G1 DNA damage checkpoint | 6.18E-05 | 0.00036811 | 0.000103768 |
| BP | GO:0044819 | mitotic G1/S transition checkpoint | 6.18E-05 | 0.00036811 | 0.000103768 |
| BP | GO:0046622 | positive regulation of organ growth | 6.18E-05 | 0.00036811 | 0.000103768 |
| BP | GO:1904377 | positive regulation of protein localization to cell periphery | 6.18E-05 | 0.00036811 | 0.000103768 |
| BP | GO:0048771 | tissue remodeling | 6.27E-05 | 0.000372817 | 0.000105095 |
| BP | GO:0043534 | blood vessel endothelial cell migration | 6.41E-05 | 0.000375056 | 0.000105726 |
| BP | GO:1905330 | regulation of morphogenesis of an epithelium | 6.41E-05 | 0.000375056 | 0.000105726 |
| BP | GO:0044783 | G1 DNA damage checkpoint | 6.48E-05 | 0.000375056 | 0.000105726 |
| BP | GO:0045600 | positive regulation of fat cell differentiation | 6.48E-05 | 0.000375056 | 0.000105726 |
| BP | GO:0060135 | maternal process involved in female pregnancy | 6.48E-05 | 0.000375056 | 0.000105726 |
| BP | GO:0001660 | fever generation | 6.49E-05 | 0.000375056 | 0.000105726 |
| BP | GO:0010749 | regulation of nitric oxide mediated signal transduction | 6.49E-05 | 0.000375056 | 0.000105726 |
| BP | GO:0070391 | response to lipoteichoic acid | 6.49E-05 | 0.000375056 | 0.000105726 |
| BP | GO:0071223 | cellular response to lipoteichoic acid | 6.49E-05 | 0.000375056 | 0.000105726 |
| BP | GO:0072203 | cell proliferation involved in metanephros development | 6.49E-05 | 0.000375056 | 0.000105726 |
| BP | GO:1903799 | negative regulation of production of miRNAs involved in gene silencing by miRNA | 6.49E-05 | 0.000375056 | 0.000105726 |
| BP | GO:1903800 | positive regulation of production of miRNAs involved in gene silencing by miRNA | 6.49E-05 | 0.000375056 | 0.000105726 |
| BP | GO:0048015 | phosphatidylinositol-mediated signaling | 6.55E-05 | 0.000376721 | 0.000106196 |
| BP | GO:0090130 | tissue migration | 6.55E-05 | 0.000376721 | 0.000106196 |
| BP | GO:0061138 | morphogenesis of a branching epithelium | 6.69E-05 | 0.000383853 | 0.000108206 |
| BP | GO:0018108 | peptidyl-tyrosine phosphorylation | 6.82E-05 | 0.000390005 | 0.00010994 |
| BP | GO:0048017 | inositol lipid-mediated signaling | 6.98E-05 | 0.000397729 | 0.000112118 |
| BP | GO:0048639 | positive regulation of developmental growth | 6.98E-05 | 0.000397729 | 0.000112118 |
| BP | GO:0018212 | peptidyl-tyrosine modification | 7.09E-05 | 0.000402699 | 0.000113518 |
| BP | GO:0045428 | regulation of nitric oxide biosynthetic process | 7.10E-05 | 0.00040278 | 0.000113541 |
| BP | GO:0046328 | regulation of JNK cascade | 7.13E-05 | 0.000403399 | 0.000113716 |
| BP | GO:0051098 | regulation of binding | 7.75E-05 | 0.000437403 | 0.000123301 |
| BP | GO:0046323 | glucose import | 7.77E-05 | 0.000437403 | 0.000123301 |
| BP | GO:0031652 | positive regulation of heat generation | 7.93E-05 | 0.000441552 | 0.000124471 |
| BP | GO:0032494 | response to peptidoglycan | 7.93E-05 | 0.000441552 | 0.000124471 |
| BP | GO:0045899 | positive regulation of RNA polymerase II transcriptional preinitiation complex assembly | 7.93E-05 | 0.000441552 | 0.000124471 |
| BP | GO:0046886 | positive regulation of hormone biosynthetic process | 7.93E-05 | 0.000441552 | 0.000124471 |
| BP | GO:0051974 | negative regulation of telomerase activity | 7.93E-05 | 0.000441552 | 0.000124471 |
| BP | GO:0071478 | cellular response to radiation | 8.07E-05 | 0.000448357 | 0.000126389 |
| BP | GO:0042698 | ovulation cycle | 8.11E-05 | 0.000449747 | 0.000126781 |
| BP | GO:0043112 | receptor metabolic process | 8.24E-05 | 0.000454459 | 0.000128109 |
| BP | GO:0050731 | positive regulation of peptidyl-tyrosine phosphorylation | 8.24E-05 | 0.000454459 | 0.000128109 |
| BP | GO:0050766 | positive regulation of phagocytosis | 8.47E-05 | 0.000466371 | 0.000131467 |
| BP | GO:0042176 | regulation of protein catabolic process | 8.57E-05 | 0.000470617 | 0.000132664 |
| BP | GO:0071479 | cellular response to ionizing radiation | 8.84E-05 | 0.000484397 | 0.000136549 |
| BP | GO:0001763 | morphogenesis of a branching structure | 8.92E-05 | 0.000487884 | 0.000137532 |
| BP | GO:0002221 | pattern recognition receptor signaling pathway | 9.10E-05 | 0.000496491 | 0.000139958 |
| BP | GO:0038034 | signal transduction in absence of ligand | 9.21E-05 | 0.000500612 | 0.00014112 |
| BP | GO:0097192 | extrinsic apoptotic signaling pathway in absence of ligand | 9.21E-05 | 0.000500612 | 0.00014112 |
| BP | GO:0033197 | response to vitamin E | 9.51E-05 | 0.00051328 | 0.000144691 |
| BP | GO:0042368 | vitamin D biosynthetic process | 9.51E-05 | 0.00051328 | 0.000144691 |
| BP | GO:0045080 | positive regulation of chemokine biosynthetic process | 9.51E-05 | 0.00051328 | 0.000144691 |
| BP | GO:0014706 | striated muscle tissue development | 9.56E-05 | 0.000514858 | 0.000145135 |
| BP | GO:0006801 | superoxide metabolic process | 9.60E-05 | 0.000514858 | 0.000145135 |
| BP | GO:0045685 | regulation of glial cell differentiation | 9.60E-05 | 0.000514858 | 0.000145135 |
| BP | GO:1900182 | positive regulation of protein localization to nucleus | 1.00E-04 | 0.000535021 | 0.000150819 |
| BP | GO:0030278 | regulation of ossification | 0.000102176 | 0.000545527 | 0.000153781 |
| BP | GO:0035265 | organ growth | 0.000104134 | 0.000553592 | 0.000156054 |
| BP | GO:0045766 | positive regulation of angiogenesis | 0.000104134 | 0.000553592 | 0.000156054 |
| BP | GO:1903532 | positive regulation of secretion by cell | 0.000106476 | 0.000564826 | 0.000159221 |
| BP | GO:0001937 | negative regulation of endothelial cell proliferation | 0.000108265 | 0.000571859 | 0.000161204 |
| BP | GO:0072088 | nephron epithelium morphogenesis | 0.000108265 | 0.000571859 | 0.000161204 |
| BP | GO:0071456 | cellular response to hypoxia | 0.000110174 | 0.000580704 | 0.000163697 |
| BP | GO:0007623 | circadian rhythm | 0.000112243 | 0.000582005 | 0.000164064 |
| BP | GO:0050792 | regulation of viral process | 0.000112243 | 0.000582005 | 0.000164064 |
| BP | GO:0010745 | negative regulation of macrophage derived foam cell differentiation | 0.000112304 | 0.000582005 | 0.000164064 |
| BP | GO:0030656 | regulation of vitamin metabolic process | 0.000112304 | 0.000582005 | 0.000164064 |
| BP | GO:0031650 | regulation of heat generation | 0.000112304 | 0.000582005 | 0.000164064 |
| BP | GO:0061029 | eyelid development in camera-type eye | 0.000112304 | 0.000582005 | 0.000164064 |
| BP | GO:0070431 | nucleotide-binding oligomerization domain containing 2 signaling pathway | 0.000112304 | 0.000582005 | 0.000164064 |
| BP | GO:0090399 | replicative senescence | 0.000112304 | 0.000582005 | 0.000164064 |
| BP | GO:0043900 | regulation of multi-organism process | 0.000114212 | 0.000590077 | 0.000166339 |
| BP | GO:0006006 | glucose metabolic process | 0.000114339 | 0.000590077 | 0.000166339 |
| BP | GO:0072028 | nephron morphogenesis | 0.000116972 | 0.000601152 | 0.000169461 |
| BP | GO:0090398 | cellular senescence | 0.000116972 | 0.000601152 | 0.000169461 |
| BP | GO:0060537 | muscle tissue development | 0.000118239 | 0.000606403 | 0.000170942 |
| BP | GO:0019216 | regulation of lipid metabolic process | 0.000120984 | 0.000619196 | 0.000174548 |
| BP | GO:0043536 | positive regulation of blood vessel endothelial cell migration | 0.000121491 | 0.00062051 | 0.000174918 |
| BP | GO:0007254 | JNK cascade | 0.000125253 | 0.000638402 | 0.000179962 |
| BP | GO:0032388 | positive regulation of intracellular transport | 0.000127523 | 0.000648636 | 0.000182847 |
| BP | GO:0002699 | positive regulation of immune effector process | 0.000129823 | 0.00065898 | 0.000185762 |
| BP | GO:0045651 | positive regulation of macrophage differentiation | 0.000130924 | 0.000661847 | 0.000186571 |
| BP | GO:0050930 | induction of positive chemotaxis | 0.000130924 | 0.000661847 | 0.000186571 |
| BP | GO:0008406 | gonad development | 0.000132153 | 0.00066399 | 0.000187175 |
| BP | GO:0036294 | cellular response to decreased oxygen levels | 0.000132153 | 0.00066399 | 0.000187175 |
| BP | GO:0050920 | regulation of chemotaxis | 0.000132153 | 0.00066399 | 0.000187175 |
| BP | GO:0032642 | regulation of chemokine production | 0.00013573 | 0.000679199 | 0.000191462 |
| BP | GO:0034644 | cellular response to UV | 0.00013573 | 0.000679199 | 0.000191462 |
| BP | GO:0048762 | mesenchymal cell differentiation | 0.000136904 | 0.000683692 | 0.000192729 |
| BP | GO:0002526 | acute inflammatory response | 0.000139326 | 0.000694382 | 0.000195742 |
| BP | GO:0006970 | response to osmotic stress | 0.000140707 | 0.000699854 | 0.000197285 |
| BP | GO:0043903 | regulation of symbiosis, encompassing mutualism through parasitism | 0.000144262 | 0.000716095 | 0.000201863 |
| BP | GO:0045445 | myoblast differentiation | 0.000145801 | 0.00072084 | 0.000203201 |
| BP | GO:0046889 | positive regulation of lipid biosynthetic process | 0.000145801 | 0.00072084 | 0.000203201 |
| BP | GO:0045137 | development of primary sexual characteristics | 0.000146777 | 0.000724217 | 0.000204152 |
| BP | GO:0051047 | positive regulation of secretion | 0.000147956 | 0.000728581 | 0.000205383 |
| BP | GO:0032352 | positive regulation of hormone metabolic process | 0.000150952 | 0.000731675 | 0.000206255 |
| BP | GO:0034349 | glial cell apoptotic process | 0.000150952 | 0.000731675 | 0.000206255 |
| BP | GO:0042362 | fat-soluble vitamin biosynthetic process | 0.000150952 | 0.000731675 | 0.000206255 |
| BP | GO:0045410 | positive regulation of interleukin-6 biosynthetic process | 0.000150952 | 0.000731675 | 0.000206255 |
| BP | GO:0045898 | regulation of RNA polymerase II transcriptional preinitiation complex assembly | 0.000150952 | 0.000731675 | 0.000206255 |
| BP | GO:0051044 | positive regulation of membrane protein ectodomain proteolysis | 0.000150952 | 0.000731675 | 0.000206255 |
| BP | GO:0072075 | metanephric mesenchyme development | 0.000150952 | 0.000731675 | 0.000206255 |
| BP | GO:1900119 | positive regulation of execution phase of apoptosis | 0.000150952 | 0.000731675 | 0.000206255 |
| BP | GO:0033157 | regulation of intracellular protein transport | 0.000151902 | 0.000734839 | 0.000207147 |
| BP | GO:0001942 | hair follicle development | 0.000156347 | 0.000751924 | 0.000211963 |
| BP | GO:0006919 | activation of cysteine-type endopeptidase activity involved in apoptotic process | 0.000156347 | 0.000751924 | 0.000211963 |
| BP | GO:0008625 | extrinsic apoptotic signaling pathway via death domain receptors | 0.000156347 | 0.000751924 | 0.000211963 |
| BP | GO:0043434 | response to peptide hormone | 0.00016133 | 0.000774384 | 0.000218294 |
| BP | GO:0010594 | regulation of endothelial cell migration | 0.000162541 | 0.000778684 | 0.000219506 |
| BP | GO:1904018 | positive regulation of vasculature development | 0.000165283 | 0.000790288 | 0.000222777 |
| BP | GO:0019932 | second-messenger-mediated signaling | 0.000166578 | 0.000792634 | 0.000223439 |
| BP | GO:0022404 | molting cycle process | 0.000167377 | 0.000792634 | 0.000223439 |
| BP | GO:0022405 | hair cycle process | 0.000167377 | 0.000792634 | 0.000223439 |
| BP | GO:0034103 | regulation of tissue remodeling | 0.000167377 | 0.000792634 | 0.000223439 |
| BP | GO:0098773 | skin epidermis development | 0.000167377 | 0.000792634 | 0.000223439 |
| BP | GO:0002739 | regulation of cytokine secretion involved in immune response | 0.000172388 | 0.0008117 | 0.000228813 |
| BP | GO:0034116 | positive regulation of heterotypic cell-cell adhesion | 0.000172388 | 0.0008117 | 0.000228813 |
| BP | GO:0071380 | cellular response to prostaglandin E stimulus | 0.000172388 | 0.0008117 | 0.000228813 |
| BP | GO:0032602 | chemokine production | 0.000173076 | 0.000813392 | 0.00022929 |
| BP | GO:0031058 | positive regulation of histone modification | 0.000178901 | 0.000837203 | 0.000236003 |
| BP | GO:0071674 | mononuclear cell migration | 0.000178901 | 0.000837203 | 0.000236003 |
| BP | GO:0006469 | negative regulation of protein kinase activity | 0.000179497 | 0.000837203 | 0.000236003 |
| BP | GO:0046777 | protein autophosphorylation | 0.000179497 | 0.000837203 | 0.000236003 |
| BP | GO:0007265 | Ras protein signal transduction | 0.00018311 | 0.000852443 | 0.000240299 |
| BP | GO:0060333 | interferon-gamma-mediated signaling pathway | 0.000184851 | 0.000858932 | 0.000242128 |
| BP | GO:0051607 | defense response to virus | 0.00018844 | 0.000873963 | 0.000246365 |
| BP | GO:0045833 | negative regulation of lipid metabolic process | 0.000190929 | 0.000883849 | 0.000249152 |
| BP | GO:0051402 | neuron apoptotic process | 0.000191491 | 0.000884794 | 0.000249418 |
| BP | GO:0006978 | DNA damage response, signal transduction by p53 class mediator resulting in transcription of p21 class mediator | 0.000195226 | 0.000890405 | 0.000251 |
| BP | GO:0010224 | response to UV-B | 0.000195226 | 0.000890405 | 0.000251 |
| BP | GO:0031649 | heat generation | 0.000195226 | 0.000890405 | 0.000251 |
| BP | GO:0032966 | negative regulation of collagen biosynthetic process | 0.000195226 | 0.000890405 | 0.000251 |
| BP | GO:0071850 | mitotic cell cycle arrest | 0.000195226 | 0.000890405 | 0.000251 |
| BP | GO:1904996 | positive regulation of leukocyte adhesion to vascular endothelial cell | 0.000195226 | 0.000890405 | 0.000251 |
| BP | GO:1905331 | negative regulation of morphogenesis of an epithelium | 0.000195226 | 0.000890405 | 0.000251 |
| BP | GO:0072080 | nephron tubule development | 0.000197134 | 0.000895802 | 0.000252521 |
| BP | GO:1901655 | cellular response to ketone | 0.000197134 | 0.000895802 | 0.000252521 |
| BP | GO:0038093 | Fc receptor signaling pathway | 0.0001977 | 0.000896723 | 0.000252781 |
| BP | GO:0002697 | regulation of immune effector process | 0.000202925 | 0.000918739 | 0.000258987 |
| BP | GO:0060993 | kidney morphogenesis | 0.00020347 | 0.00091952 | 0.000259207 |
| BP | GO:0051896 | regulation of protein kinase B signaling | 0.000207285 | 0.000935052 | 0.000263586 |
| BP | GO:0001667 | ameboidal-type cell migration | 0.000209179 | 0.000938449 | 0.000264543 |
| BP | GO:0002702 | positive regulation of production of molecular mediator of immune response | 0.000209936 | 0.000938449 | 0.000264543 |
| BP | GO:0042116 | macrophage activation | 0.000209936 | 0.000938449 | 0.000264543 |
| BP | GO:0061326 | renal tubule development | 0.000209936 | 0.000938449 | 0.000264543 |
| BP | GO:1903076 | regulation of protein localization to plasma membrane | 0.000209936 | 0.000938449 | 0.000264543 |
| BP | GO:0002683 | negative regulation of immune system process | 0.000213429 | 0.000952341 | 0.000268459 |
| BP | GO:0042110 | T cell activation | 0.000215579 | 0.000957554 | 0.000269929 |
| BP | GO:0008585 | female gonad development | 0.000216534 | 0.000957554 | 0.000269929 |
| BP | GO:0019217 | regulation of fatty acid metabolic process | 0.000216534 | 0.000957554 | 0.000269929 |
| BP | GO:0032755 | positive regulation of interleukin-6 production | 0.000216534 | 0.000957554 | 0.000269929 |
| BP | GO:0048010 | vascular endothelial growth factor receptor signaling pathway | 0.000216534 | 0.000957554 | 0.000269929 |
| BP | GO:0097237 | cellular response to toxic substance | 0.000217202 | 0.000958791 | 0.000270277 |
| BP | GO:0010713 | negative regulation of collagen metabolic process | 0.000219465 | 0.000961911 | 0.000271157 |
| BP | GO:0031065 | positive regulation of histone deacetylation | 0.000219465 | 0.000961911 | 0.000271157 |
| BP | GO:0042772 | DNA damage response, signal transduction resulting in transcription | 0.000219465 | 0.000961911 | 0.000271157 |
| BP | GO:0051023 | regulation of immunoglobulin secretion | 0.000219465 | 0.000961911 | 0.000271157 |
| BP | GO:0051346 | negative regulation of hydrolase activity | 0.000219928 | 0.000962236 | 0.000271249 |
| BP | GO:1901991 | negative regulation of mitotic cell cycle phase transition | 0.000220582 | 0.000963391 | 0.000271574 |
| BP | GO:0015908 | fatty acid transport | 0.000223266 | 0.000969969 | 0.000273429 |
| BP | GO:0030316 | osteoclast differentiation | 0.000223266 | 0.000969969 | 0.000273429 |
| BP | GO:0044773 | mitotic DNA damage checkpoint | 0.000223266 | 0.000969969 | 0.000273429 |
| BP | GO:0019318 | hexose metabolic process | 0.000224001 | 0.000971456 | 0.000273848 |
| BP | GO:0030072 | peptide hormone secretion | 0.000227457 | 0.000982997 | 0.000277101 |
| BP | GO:0071383 | cellular response to steroid hormone stimulus | 0.000227457 | 0.000982997 | 0.000277101 |
| BP | GO:0050764 | regulation of phagocytosis | 0.000230131 | 0.000992818 | 0.000279869 |
| BP | GO:0050708 | regulation of protein secretion | 0.000233381 | 0.001005082 | 0.000283327 |
| BP | GO:0019395 | fatty acid oxidation | 0.000237132 | 0.001017693 | 0.000286882 |
| BP | GO:0048525 | negative regulation of viral process | 0.000237132 | 0.001017693 | 0.000286882 |
| BP | GO:0002824 | positive regulation of adaptive immune response based on somatic recombination of immune receptors built from immunoglobulin superfamily domains | 0.00024427 | 0.001039261 | 0.000292961 |
| BP | GO:0002726 | positive regulation of T cell cytokine production | 0.000245101 | 0.001039261 | 0.000292961 |
| BP | GO:0002922 | positive regulation of humoral immune response | 0.000245101 | 0.001039261 | 0.000292961 |
| BP | GO:0030949 | positive regulation of vascular endothelial growth factor receptor signaling pathway | 0.000245101 | 0.001039261 | 0.000292961 |
| BP | GO:0060716 | labyrinthine layer blood vessel development | 0.000245101 | 0.001039261 | 0.000292961 |
| BP | GO:0072074 | kidney mesenchyme development | 0.000245101 | 0.001039261 | 0.000292961 |
| BP | GO:2000010 | positive regulation of protein localization to cell surface | 0.000245101 | 0.001039261 | 0.000292961 |
| BP | GO:0007611 | learning or memory | 0.000249011 | 0.001054032 | 0.000297125 |
| BP | GO:0034440 | lipid oxidation | 0.000251546 | 0.00105932 | 0.000298616 |
| BP | GO:0046545 | development of primary female sexual characteristics | 0.000251546 | 0.00105932 | 0.000298616 |
| BP | GO:1903426 | regulation of reactive oxygen species biosynthetic process | 0.000251546 | 0.00105932 | 0.000298616 |
| BP | GO:0033673 | negative regulation of kinase activity | 0.000252741 | 0.001062545 | 0.000299525 |
| BP | GO:0003007 | heart morphogenesis | 0.000260323 | 0.001092561 | 0.000307986 |
| BP | GO:1905269 | positive regulation of chromatin organization | 0.000266515 | 0.001116653 | 0.000314778 |
| BP | GO:0002374 | cytokine secretion involved in immune response | 0.00027213 | 0.001134411 | 0.000319784 |
| BP | GO:0045655 | regulation of monocyte differentiation | 0.00027213 | 0.001134411 | 0.000319784 |
| BP | GO:0060261 | positive regulation of transcription initiation from RNA polymerase II promoter | 0.00027213 | 0.001134411 | 0.000319784 |
| BP | GO:0008630 | intrinsic apoptotic signaling pathway in response to DNA damage | 0.000274211 | 0.001139243 | 0.000321146 |
| BP | GO:0071887 | leukocyte apoptotic process | 0.000274211 | 0.001139243 | 0.000321146 |
| BP | GO:0002708 | positive regulation of lymphocyte mediated immunity | 0.00028205 | 0.001167883 | 0.000329219 |
| BP | GO:0002821 | positive regulation of adaptive immune response | 0.00028205 | 0.001167883 | 0.000329219 |
| BP | GO:0044774 | mitotic DNA integrity checkpoint | 0.000290032 | 0.001198927 | 0.00033797 |
| BP | GO:0072331 | signal transduction by p53 class mediator | 0.000292298 | 0.001204266 | 0.000339476 |
| BP | GO:1901988 | negative regulation of cell cycle phase transition | 0.000292298 | 0.001204266 | 0.000339476 |
| BP | GO:0010829 | negative regulation of glucose transmembrane transport | 0.00030055 | 0.001223987 | 0.000345035 |
| BP | GO:0010893 | positive regulation of steroid biosynthetic process | 0.00030055 | 0.001223987 | 0.000345035 |
| BP | GO:0035743 | CD4-positive, alpha-beta T cell cytokine production | 0.00030055 | 0.001223987 | 0.000345035 |
| BP | GO:0046827 | positive regulation of protein export from nucleus | 0.00030055 | 0.001223987 | 0.000345035 |
| BP | GO:0055093 | response to hyperoxia | 0.00030055 | 0.001223987 | 0.000345035 |
| BP | GO:0072111 | cell proliferation involved in kidney development | 0.00030055 | 0.001223987 | 0.000345035 |
| BP | GO:1903055 | positive regulation of extracellular matrix organization | 0.00030055 | 0.001223987 | 0.000345035 |
| BP | GO:0002791 | regulation of peptide secretion | 0.000304681 | 0.001237975 | 0.000348978 |
| BP | GO:0007548 | sex differentiation | 0.000304987 | 0.001237975 | 0.000348978 |
| BP | GO:0006275 | regulation of DNA replication | 0.000306431 | 0.001239767 | 0.000349483 |
| BP | GO:0071156 | regulation of cell cycle arrest | 0.000306431 | 0.001239767 | 0.000349483 |
| BP | GO:0046651 | lymphocyte proliferation | 0.000313663 | 0.001266953 | 0.000357147 |
| BP | GO:0072009 | nephron epithelium development | 0.000314851 | 0.001269675 | 0.000357914 |
| BP | GO:0043542 | endothelial cell migration | 0.000318067 | 0.001280558 | 0.000360982 |
| BP | GO:0032943 | mononuclear cell proliferation | 0.000322516 | 0.001296357 | 0.000365435 |
| BP | GO:0031281 | positive regulation of cyclase activity | 0.000330357 | 0.001312932 | 0.000370108 |
| BP | GO:0042359 | vitamin D metabolic process | 0.000330357 | 0.001312932 | 0.000370108 |
| BP | GO:0045649 | regulation of macrophage differentiation | 0.000330357 | 0.001312932 | 0.000370108 |
| BP | GO:0048305 | immunoglobulin secretion | 0.000330357 | 0.001312932 | 0.000370108 |
| BP | GO:0051000 | positive regulation of nitric-oxide synthase activity | 0.000330357 | 0.001312932 | 0.000370108 |
| BP | GO:0071379 | cellular response to prostaglandin stimulus | 0.000330357 | 0.001312932 | 0.000370108 |
| BP | GO:0090312 | positive regulation of protein deacetylation | 0.000330357 | 0.001312932 | 0.000370108 |
| BP | GO:1904019 | epithelial cell apoptotic process | 0.000332136 | 0.00131788 | 0.000371503 |
| BP | GO:0001938 | positive regulation of endothelial cell proliferation | 0.000341003 | 0.001346581 | 0.000379593 |
| BP | GO:0042303 | molting cycle | 0.000341003 | 0.001346581 | 0.000379593 |
| BP | GO:0042633 | hair cycle | 0.000341003 | 0.001346581 | 0.000379593 |
| BP | GO:0043200 | response to amino acid | 0.000350022 | 0.001377794 | 0.000388392 |
| BP | GO:0046620 | regulation of organ growth | 0.000350022 | 0.001377794 | 0.000388392 |
| BP | GO:0030100 | regulation of endocytosis | 0.00035492 | 0.001394852 | 0.000393201 |
| BP | GO:0045446 | endothelial cell differentiation | 0.000359194 | 0.001409407 | 0.000397304 |
| BP | GO:0034695 | response to prostaglandin E | 0.000361549 | 0.001409699 | 0.000397386 |
| BP | GO:0051043 | regulation of membrane protein ectodomain proteolysis | 0.000361549 | 0.001409699 | 0.000397386 |
| BP | GO:0072215 | regulation of metanephros development | 0.000361549 | 0.001409699 | 0.000397386 |
| BP | GO:2000637 | positive regulation of gene silencing by miRNA | 0.000361549 | 0.001409699 | 0.000397386 |
| BP | GO:0016579 | protein deubiquitination | 0.000364592 | 0.001419326 | 0.0004001 |
| BP | GO:0046660 | female sex differentiation | 0.000368519 | 0.001430108 | 0.000403139 |
| BP | GO:1904375 | regulation of protein localization to cell periphery | 0.000368519 | 0.001430108 | 0.000403139 |
| BP | GO:0021700 | developmental maturation | 0.000369499 | 0.001431663 | 0.000403577 |
| BP | GO:0034976 | response to endoplasmic reticulum stress | 0.000374453 | 0.001448587 | 0.000408348 |
| BP | GO:0007569 | cell aging | 0.000377999 | 0.001457743 | 0.000410929 |
| BP | GO:1900180 | regulation of protein localization to nucleus | 0.000377999 | 0.001457743 | 0.000410929 |
| BP | GO:0002440 | production of molecular mediator of immune response | 0.000379454 | 0.001461075 | 0.000411868 |
| BP | GO:0071824 | protein-DNA complex subunit organization | 0.0003896 | 0.00149781 | 0.000422224 |
| BP | GO:0009110 | vitamin biosynthetic process | 0.000394122 | 0.001508156 | 0.00042514 |
| BP | GO:0044346 | fibroblast apoptotic process | 0.000394122 | 0.001508156 | 0.00042514 |
| BP | GO:0060148 | positive regulation of posttranscriptional gene silencing | 0.000394122 | 0.001508156 | 0.00042514 |
| BP | GO:0097193 | intrinsic apoptotic signaling pathway | 0.000394746 | 0.00150821 | 0.000425155 |
| BP | GO:0005996 | monosaccharide metabolic process | 0.000410475 | 0.001563474 | 0.000440734 |
| BP | GO:0009410 | response to xenobiotic stimulus | 0.000410475 | 0.001563474 | 0.000440734 |
| BP | GO:0051146 | striated muscle cell differentiation | 0.000415817 | 0.001581383 | 0.000445782 |
| BP | GO:0022612 | gland morphogenesis | 0.00041749 | 0.001585306 | 0.000446888 |
| BP | GO:0050768 | negative regulation of neurogenesis | 0.000426649 | 0.001608198 | 0.000453341 |
| BP | GO:0043618 | regulation of transcription from RNA polymerase II promoter in response to stress | 0.00042776 | 0.001608198 | 0.000453341 |
| BP | GO:0019430 | removal of superoxide radicals | 0.000428072 | 0.001608198 | 0.000453341 |
| BP | GO:0045672 | positive regulation of osteoclast differentiation | 0.000428072 | 0.001608198 | 0.000453341 |
| BP | GO:0050927 | positive regulation of positive chemotaxis | 0.000428072 | 0.001608198 | 0.000453341 |
| BP | GO:0060330 | regulation of response to interferon-gamma | 0.000428072 | 0.001608198 | 0.000453341 |
| BP | GO:0060334 | regulation of interferon-gamma-mediated signaling pathway | 0.000428072 | 0.001608198 | 0.000453341 |
| BP | GO:0002758 | innate immune response-activating signal transduction | 0.000443275 | 0.001660266 | 0.000468019 |
| BP | GO:0070661 | leukocyte proliferation | 0.000443275 | 0.001660266 | 0.000468019 |
| BP | GO:0071621 | granulocyte chemotaxis | 0.000448786 | 0.001676324 | 0.000472546 |
| BP | GO:0070646 | protein modification by small protein removal | 0.000448918 | 0.001676324 | 0.000472546 |
| BP | GO:0070372 | regulation of ERK1 and ERK2 cascade | 0.000454613 | 0.001695029 | 0.000477819 |
| BP | GO:0014066 | regulation of phosphatidylinositol 3-kinase signaling | 0.000459544 | 0.001707181 | 0.000481244 |
| BP | GO:0002360 | T cell lineage commitment | 0.000463397 | 0.001707181 | 0.000481244 |
| BP | GO:0032800 | receptor biosynthetic process | 0.000463397 | 0.001707181 | 0.000481244 |
| BP | GO:0034114 | regulation of heterotypic cell-cell adhesion | 0.000463397 | 0.001707181 | 0.000481244 |
| BP | GO:0050926 | regulation of positive chemotaxis | 0.000463397 | 0.001707181 | 0.000481244 |
| BP | GO:0060706 | cell differentiation involved in embryonic placenta development | 0.000463397 | 0.001707181 | 0.000481244 |
| BP | GO:1900739 | regulation of protein insertion into mitochondrial membrane involved in apoptotic signaling pathway | 0.000463397 | 0.001707181 | 0.000481244 |
| BP | GO:1900740 | positive regulation of protein insertion into mitochondrial membrane involved in apoptotic signaling pathway | 0.000463397 | 0.001707181 | 0.000481244 |
| BP | GO:0035270 | endocrine system development | 0.00049281 | 0.001810142 | 0.000510268 |
| BP | GO:0045727 | positive regulation of translation | 0.00049281 | 0.001810142 | 0.000510268 |
| BP | GO:0009651 | response to salt stress | 0.000500094 | 0.001820668 | 0.000513235 |
| BP | GO:0035902 | response to immobilization stress | 0.000500094 | 0.001820668 | 0.000513235 |
| BP | GO:0046885 | regulation of hormone biosynthetic process | 0.000500094 | 0.001820668 | 0.000513235 |
| BP | GO:0071450 | cellular response to oxygen radical | 0.000500094 | 0.001820668 | 0.000513235 |
| BP | GO:0071451 | cellular response to superoxide | 0.000500094 | 0.001820668 | 0.000513235 |
| BP | GO:2000191 | regulation of fatty acid transport | 0.000500094 | 0.001820668 | 0.000513235 |
| BP | GO:0010595 | positive regulation of endothelial cell migration | 0.000504232 | 0.001830341 | 0.000515962 |
| BP | GO:0034763 | negative regulation of transmembrane transport | 0.000504232 | 0.001830341 | 0.000515962 |
| BP | GO:0042113 | B cell activation | 0.000514445 | 0.001864673 | 0.00052564 |
| BP | GO:0071482 | cellular response to light stimulus | 0.000515824 | 0.001866934 | 0.000526278 |
| BP | GO:0046879 | hormone secretion | 0.000527054 | 0.001903926 | 0.000536705 |
| BP | GO:0050671 | positive regulation of lymphocyte proliferation | 0.000527585 | 0.001903926 | 0.000536705 |
| BP | GO:0003180 | aortic valve morphogenesis | 0.00053816 | 0.00191967 | 0.000541144 |
| BP | GO:0036296 | response to increased oxygen levels | 0.00053816 | 0.00191967 | 0.000541144 |
| BP | GO:0045408 | regulation of interleukin-6 biosynthetic process | 0.00053816 | 0.00191967 | 0.000541144 |
| BP | GO:0051123 | RNA polymerase II preinitiation complex assembly | 0.00053816 | 0.00191967 | 0.000541144 |
| BP | GO:0060260 | regulation of transcription initiation from RNA polymerase II promoter | 0.00053816 | 0.00191967 | 0.000541144 |
| BP | GO:0071168 | protein localization to chromatin | 0.00053816 | 0.00191967 | 0.000541144 |
| BP | GO:1902932 | positive regulation of alcohol biosynthetic process | 0.00053816 | 0.00191967 | 0.000541144 |
| BP | GO:1904994 | regulation of leukocyte adhesion to vascular endothelial cell | 0.00053816 | 0.00191967 | 0.000541144 |
| BP | GO:0032946 | positive regulation of mononuclear cell proliferation | 0.000539517 | 0.001921737 | 0.000541726 |
| BP | GO:0051961 | negative regulation of nervous system development | 0.00054638 | 0.001943382 | 0.000547828 |
| BP | GO:0003158 | endothelium development | 0.000551621 | 0.001959205 | 0.000552288 |
| BP | GO:0002705 | positive regulation of leukocyte mediated immunity | 0.000563898 | 0.00199707 | 0.000562962 |
| BP | GO:0042770 | signal transduction in response to DNA damage | 0.000563898 | 0.00199707 | 0.000562962 |
| BP | GO:0002218 | activation of innate immune response | 0.000572928 | 0.00202615 | 0.00057116 |
| BP | GO:0000303 | response to superoxide | 0.000577591 | 0.002028131 | 0.000571718 |
| BP | GO:0010575 | positive regulation of vascular endothelial growth factor production | 0.000577591 | 0.002028131 | 0.000571718 |
| BP | GO:0031063 | regulation of histone deacetylation | 0.000577591 | 0.002028131 | 0.000571718 |
| BP | GO:0042226 | interleukin-6 biosynthetic process | 0.000577591 | 0.002028131 | 0.000571718 |
| BP | GO:0070102 | interleukin-6-mediated signaling pathway | 0.000577591 | 0.002028131 | 0.000571718 |
| BP | GO:0009914 | hormone transport | 0.000593435 | 0.002077864 | 0.000585738 |
| BP | GO:0060562 | epithelial tube morphogenesis | 0.000593435 | 0.002077864 | 0.000585738 |
| BP | GO:0072655 | establishment of protein localization to mitochondrion | 0.000614755 | 0.002140959 | 0.000603524 |
| BP | GO:0000305 | response to oxygen radical | 0.000618384 | 0.002140959 | 0.000603524 |
| BP | GO:0001844 | protein insertion into mitochondrial membrane involved in apoptotic signaling pathway | 0.000618384 | 0.002140959 | 0.000603524 |
| BP | GO:0010743 | regulation of macrophage derived foam cell differentiation | 0.000618384 | 0.002140959 | 0.000603524 |
| BP | GO:0045940 | positive regulation of steroid metabolic process | 0.000618384 | 0.002140959 | 0.000603524 |
| BP | GO:0046320 | regulation of fatty acid oxidation | 0.000618384 | 0.002140959 | 0.000603524 |
| BP | GO:0071480 | cellular response to gamma radiation | 0.000618384 | 0.002140959 | 0.000603524 |
| BP | GO:0097421 | liver regeneration | 0.000618384 | 0.002140959 | 0.000603524 |
| BP | GO:0050715 | positive regulation of cytokine secretion | 0.000641249 | 0.00221392 | 0.000624091 |
| BP | GO:0070665 | positive regulation of leukocyte proliferation | 0.000641249 | 0.00221392 | 0.000624091 |
| BP | GO:0001655 | urogenital system development | 0.000650676 | 0.002243334 | 0.000632382 |
| BP | GO:0072073 | kidney epithelium development | 0.000654765 | 0.002254288 | 0.00063547 |
| BP | GO:0002724 | regulation of T cell cytokine production | 0.000660536 | 0.002264695 | 0.000638404 |
| BP | GO:0034694 | response to prostaglandin | 0.000660536 | 0.002264695 | 0.000638404 |
| BP | GO:0045648 | positive regulation of erythrocyte differentiation | 0.000660536 | 0.002264695 | 0.000638404 |
| BP | GO:0008286 | insulin receptor signaling pathway | 0.000668462 | 0.002282374 | 0.000643388 |
| BP | GO:0070585 | protein localization to mitochondrion | 0.000668462 | 0.002282374 | 0.000643388 |
| BP | GO:0097530 | granulocyte migration | 0.000668462 | 0.002282374 | 0.000643388 |
| BP | GO:0051251 | positive regulation of lymphocyte activation | 0.000680723 | 0.002320149 | 0.000654036 |
| BP | GO:0038127 | ERBB signaling pathway | 0.000682342 | 0.002320149 | 0.000654036 |
| BP | GO:0072006 | nephron development | 0.000682342 | 0.002320149 | 0.000654036 |
| BP | GO:0031056 | regulation of histone modification | 0.000696404 | 0.002355008 | 0.000663863 |
| BP | GO:0035296 | regulation of tube diameter | 0.000696404 | 0.002355008 | 0.000663863 |
| BP | GO:0050880 | regulation of blood vessel size | 0.000696404 | 0.002355008 | 0.000663863 |
| BP | GO:0097746 | regulation of blood vessel diameter | 0.000696404 | 0.002355008 | 0.000663863 |
| BP | GO:0003176 | aortic valve development | 0.000704044 | 0.002361461 | 0.000665682 |
| BP | GO:0045907 | positive regulation of vasoconstriction | 0.000704044 | 0.002361461 | 0.000665682 |
| BP | GO:0055094 | response to lipoprotein particle | 0.000704044 | 0.002361461 | 0.000665682 |
| BP | GO:0060674 | placenta blood vessel development | 0.000704044 | 0.002361461 | 0.000665682 |
| BP | GO:1900745 | positive regulation of p38MAPK cascade | 0.000704044 | 0.002361461 | 0.000665682 |
| BP | GO:2000778 | positive regulation of interleukin-6 secretion | 0.000704044 | 0.002361461 | 0.000665682 |
| BP | GO:0035150 | regulation of tube size | 0.000710651 | 0.002380391 | 0.000671018 |
| BP | GO:0002822 | regulation of adaptive immune response based on somatic recombination of immune receptors built from immunoglobulin superfamily domains | 0.000725082 | 0.002425444 | 0.000683718 |
| BP | GO:0002224 | toll-like receptor signaling pathway | 0.0007397 | 0.002470998 | 0.00069656 |
| BP | GO:0016242 | negative regulation of macroautophagy | 0.000748905 | 0.002488298 | 0.000701436 |
| BP | GO:0036003 | positive regulation of transcription from RNA polymerase II promoter in response to stress | 0.000748905 | 0.002488298 | 0.000701436 |
| BP | GO:0051204 | protein insertion into mitochondrial membrane | 0.000748905 | 0.002488298 | 0.000701436 |
| BP | GO:1902692 | regulation of neuroblast proliferation | 0.000748905 | 0.002488298 | 0.000701436 |
| BP | GO:0071236 | cellular response to antibiotic | 0.000754505 | 0.002503537 | 0.000705732 |
| BP | GO:0002706 | regulation of lymphocyte mediated immunity | 0.000784679 | 0.002596689 | 0.000731991 |
| BP | GO:0051092 | positive regulation of NF-kappaB transcription factor activity | 0.000784679 | 0.002596689 | 0.000731991 |
| BP | GO:0007435 | salivary gland morphogenesis | 0.000795116 | 0.002617213 | 0.000737777 |
| BP | GO:0010574 | regulation of vascular endothelial growth factor production | 0.000795116 | 0.002617213 | 0.000737777 |
| BP | GO:0048566 | embryonic digestive tract development | 0.000795116 | 0.002617213 | 0.000737777 |
| BP | GO:0071402 | cellular response to lipoprotein particle stimulus | 0.000795116 | 0.002617213 | 0.000737777 |
| BP | GO:0007178 | transmembrane receptor protein serine/threonine kinase signaling pathway | 0.000802234 | 0.002637131 | 0.000743391 |
| BP | GO:0032675 | regulation of interleukin-6 production | 0.000831367 | 0.00272927 | 0.000769365 |
| BP | GO:0001893 | maternal placenta development | 0.000842674 | 0.002751769 | 0.000775707 |
| BP | GO:0032735 | positive regulation of interleukin-12 production | 0.000842674 | 0.002751769 | 0.000775707 |
| BP | GO:0110111 | negative regulation of animal organ morphogenesis | 0.000842674 | 0.002751769 | 0.000775707 |
| BP | GO:1901030 | positive regulation of mitochondrial outer membrane permeabilization involved in apoptotic signaling pathway | 0.000842674 | 0.002751769 | 0.000775707 |
| BP | GO:0001505 | regulation of neurotransmitter levels | 0.000845949 | 0.002756002 | 0.000776901 |
| BP | GO:0030168 | platelet activation | 0.000847314 | 0.002756002 | 0.000776901 |
| BP | GO:0034250 | positive regulation of cellular amide metabolic process | 0.000847314 | 0.002756002 | 0.000776901 |
| BP | GO:0006959 | humoral immune response | 0.000863899 | 0.002806252 | 0.000791066 |
| BP | GO:0001666 | response to hypoxia | 0.000891328 | 0.002866024 | 0.000807915 |
| BP | GO:0010573 | vascular endothelial growth factor production | 0.000891575 | 0.002866024 | 0.000807915 |
| BP | GO:0030947 | regulation of vascular endothelial growth factor receptor signaling pathway | 0.000891575 | 0.002866024 | 0.000807915 |
| BP | GO:0045740 | positive regulation of DNA replication | 0.000891575 | 0.002866024 | 0.000807915 |
| BP | GO:0046627 | negative regulation of insulin receptor signaling pathway | 0.000891575 | 0.002866024 | 0.000807915 |
| BP | GO:0051973 | positive regulation of telomerase activity | 0.000891575 | 0.002866024 | 0.000807915 |
| BP | GO:0071392 | cellular response to estradiol stimulus | 0.000891575 | 0.002866024 | 0.000807915 |
| BP | GO:0090322 | regulation of superoxide metabolic process | 0.000891575 | 0.002866024 | 0.000807915 |
| BP | GO:0007259 | JAK-STAT cascade | 0.000896322 | 0.002877544 | 0.000811163 |
| BP | GO:0010948 | negative regulation of cell cycle process | 0.000909953 | 0.002913737 | 0.000821365 |
| BP | GO:0031331 | positive regulation of cellular catabolic process | 0.000909953 | 0.002913737 | 0.000821365 |
| BP | GO:0007431 | salivary gland development | 0.000941816 | 0.002981011 | 0.000840329 |
| BP | GO:0014037 | Schwann cell differentiation | 0.000941816 | 0.002981011 | 0.000840329 |
| BP | GO:0030212 | hyaluronan metabolic process | 0.000941816 | 0.002981011 | 0.000840329 |
| BP | GO:0034405 | response to fluid shear stress | 0.000941816 | 0.002981011 | 0.000840329 |
| BP | GO:0038083 | peptidyl-tyrosine autophosphorylation | 0.000941816 | 0.002981011 | 0.000840329 |
| BP | GO:0042307 | positive regulation of protein import into nucleus | 0.000941816 | 0.002981011 | 0.000840329 |
| BP | GO:0045923 | positive regulation of fatty acid metabolic process | 0.000941816 | 0.002981011 | 0.000840329 |
| BP | GO:0050691 | regulation of defense response to virus by host | 0.000941816 | 0.002981011 | 0.000840329 |
| BP | GO:1905314 | semi-lunar valve development | 0.000941816 | 0.002981011 | 0.000840329 |
| BP | GO:0006869 | lipid transport | 0.000948029 | 0.002993012 | 0.000843712 |
| BP | GO:0032102 | negative regulation of response to external stimulus | 0.000948029 | 0.002993012 | 0.000843712 |
| BP | GO:0002819 | regulation of adaptive immune response | 0.000964423 | 0.003037011 | 0.000856115 |
| BP | GO:0007519 | skeletal muscle tissue development | 0.000964423 | 0.003037011 | 0.000856115 |
| BP | GO:0032635 | interleukin-6 production | 0.000981947 | 0.00308826 | 0.000870562 |
| BP | GO:0006909 | phagocytosis | 0.00098722 | 0.003092787 | 0.000871838 |
| BP | GO:0032350 | regulation of hormone metabolic process | 0.000993395 | 0.003092787 | 0.000871838 |
| BP | GO:0046326 | positive regulation of glucose import | 0.000993395 | 0.003092787 | 0.000871838 |
| BP | GO:0046825 | regulation of protein export from nucleus | 0.000993395 | 0.003092787 | 0.000871838 |
| BP | GO:0060045 | positive regulation of cardiac muscle cell proliferation | 0.000993395 | 0.003092787 | 0.000871838 |
| BP | GO:1900077 | negative regulation of cellular response to insulin stimulus | 0.000993395 | 0.003092787 | 0.000871838 |
| BP | GO:1904706 | negative regulation of vascular smooth muscle cell proliferation | 0.000993395 | 0.003092787 | 0.000871838 |
| BP | GO:2000279 | negative regulation of DNA biosynthetic process | 0.000993395 | 0.003092787 | 0.000871838 |
| BP | GO:0036293 | response to decreased oxygen levels | 0.000997194 | 0.003100708 | 0.000874071 |
| BP | GO:0015718 | monocarboxylic acid transport | 0.000999672 | 0.003104508 | 0.000875142 |
| BP | GO:0010661 | positive regulation of muscle cell apoptotic process | 0.001046308 | 0.00323714 | 0.00091253 |
| BP | GO:1902042 | negative regulation of extrinsic apoptotic signaling pathway via death domain receptors | 0.001046308 | 0.00323714 | 0.00091253 |
| BP | GO:1904591 | positive regulation of protein import | 0.001046308 | 0.00323714 | 0.00091253 |
| BP | GO:0097696 | STAT cascade | 0.001072602 | 0.00331434 | 0.000934293 |
| BP | GO:0035051 | cardiocyte differentiation | 0.001091347 | 0.003368051 | 0.000949434 |
| BP | GO:0002369 | T cell cytokine production | 0.001100553 | 0.003376428 | 0.000951795 |
| BP | GO:0045124 | regulation of bone resorption | 0.001100553 | 0.003376428 | 0.000951795 |
| BP | GO:0090184 | positive regulation of kidney development | 0.001100553 | 0.003376428 | 0.000951795 |
| BP | GO:2000008 | regulation of protein localization to cell surface | 0.001100553 | 0.003376428 | 0.000951795 |
| BP | GO:0002696 | positive regulation of leukocyte activation | 0.00110089 | 0.003376428 | 0.000951795 |
| BP | GO:0050806 | positive regulation of synaptic transmission | 0.001110298 | 0.003396821 | 0.000957544 |
| BP | GO:0051302 | regulation of cell division | 0.001110298 | 0.003396821 | 0.000957544 |
| BP | GO:0045089 | positive regulation of innate immune response | 0.001111662 | 0.003396821 | 0.000957544 |
| BP | GO:0030307 | positive regulation of cell growth | 0.001129457 | 0.003438447 | 0.000969278 |
| BP | GO:0051100 | negative regulation of binding | 0.001129457 | 0.003438447 | 0.000969278 |
| BP | GO:0060538 | skeletal muscle organ development | 0.001129457 | 0.003438447 | 0.000969278 |
| BP | GO:0006631 | fatty acid metabolic process | 0.001133428 | 0.00344629 | 0.000971489 |
| BP | GO:0043433 | negative regulation of DNA-binding transcription factor activity | 0.001148825 | 0.003488815 | 0.000983476 |
| BP | GO:0042692 | muscle cell differentiation | 0.001155493 | 0.003498094 | 0.000986092 |
| BP | GO:1900117 | regulation of execution phase of apoptosis | 0.001156126 | 0.003498094 | 0.000986092 |
| BP | GO:1903053 | regulation of extracellular matrix organization | 0.001156126 | 0.003498094 | 0.000986092 |
| BP | GO:0043901 | negative regulation of multi-organism process | 0.001168402 | 0.003530917 | 0.000995345 |
| BP | GO:0032874 | positive regulation of stress-activated MAPK cascade | 0.001188189 | 0.00358633 | 0.001010965 |
| BP | GO:0070304 | positive regulation of stress-activated protein kinase signaling cascade | 0.001208188 | 0.003642244 | 0.001026727 |
| BP | GO:0006509 | membrane protein ectodomain proteolysis | 0.001213024 | 0.003652368 | 0.001029581 |
| BP | GO:0050867 | positive regulation of cell activation | 0.001258549 | 0.003784832 | 0.001066922 |
| BP | GO:0051897 | positive regulation of protein kinase B signaling | 0.001269459 | 0.003790728 | 0.001068584 |
| BP | GO:0006775 | fat-soluble vitamin metabolic process | 0.001271243 | 0.003790728 | 0.001068584 |
| BP | GO:0010863 | positive regulation of phospholipase C activity | 0.001271243 | 0.003790728 | 0.001068584 |
| BP | GO:0031279 | regulation of cyclase activity | 0.001271243 | 0.003790728 | 0.001068584 |
| BP | GO:0034198 | cellular response to amino acid starvation | 0.001271243 | 0.003790728 | 0.001068584 |
| BP | GO:0045687 | positive regulation of glial cell differentiation | 0.001271243 | 0.003790728 | 0.001068584 |
| BP | GO:0071364 | cellular response to epidermal growth factor stimulus | 0.001271243 | 0.003790728 | 0.001068584 |
| BP | GO:0010828 | positive regulation of glucose transmembrane transport | 0.001330782 | 0.00393504 | 0.001109265 |
| BP | GO:0032965 | regulation of collagen biosynthetic process | 0.001330782 | 0.00393504 | 0.001109265 |
| BP | GO:0061756 | leukocyte adhesion to vascular endothelial cell | 0.001330782 | 0.00393504 | 0.001109265 |
| BP | GO:0070897 | transcription preinitiation complex assembly | 0.001330782 | 0.00393504 | 0.001109265 |
| BP | GO:0071622 | regulation of granulocyte chemotaxis | 0.001330782 | 0.00393504 | 0.001109265 |
| BP | GO:0090311 | regulation of protein deacetylation | 0.001330782 | 0.00393504 | 0.001109265 |
| BP | GO:1901028 | regulation of mitochondrial outer membrane permeabilization involved in apoptotic signaling pathway | 0.001330782 | 0.00393504 | 0.001109265 |
| BP | GO:0071346 | cellular response to interferon-gamma | 0.001354162 | 0.003994617 | 0.001126059 |
| BP | GO:1901796 | regulation of signal transduction by p53 class mediator | 0.001354162 | 0.003994617 | 0.001126059 |
| BP | GO:0010565 | regulation of cellular ketone metabolic process | 0.001375881 | 0.004053848 | 0.001142756 |
| BP | GO:0031018 | endocrine pancreas development | 0.001391637 | 0.004080815 | 0.001150357 |
| BP | GO:0032570 | response to progesterone | 0.001391637 | 0.004080815 | 0.001150357 |
| BP | GO:0042771 | intrinsic apoptotic signaling pathway in response to DNA damage by p53 class mediator | 0.001391637 | 0.004080815 | 0.001150357 |
| BP | GO:1900274 | regulation of phospholipase C activity | 0.001391637 | 0.004080815 | 0.001150357 |
| BP | GO:0006338 | chromatin remodeling | 0.001397819 | 0.004089241 | 0.001152733 |
| BP | GO:0010821 | regulation of mitochondrion organization | 0.001397819 | 0.004089241 | 0.001152733 |
| BP | GO:0042098 | T cell proliferation | 0.001442355 | 0.0042046 | 0.001185252 |
| BP | GO:0043401 | steroid hormone mediated signaling pathway | 0.001442355 | 0.0042046 | 0.001185252 |
| BP | GO:0043409 | negative regulation of MAPK cascade | 0.001442355 | 0.0042046 | 0.001185252 |
| BP | GO:0006984 | ER-nucleus signaling pathway | 0.001453805 | 0.004213136 | 0.001187658 |
| BP | GO:0022602 | ovulation cycle process | 0.001453805 | 0.004213136 | 0.001187658 |
| BP | GO:0043330 | response to exogenous dsRNA | 0.001453805 | 0.004213136 | 0.001187658 |
| BP | GO:0071675 | regulation of mononuclear cell migration | 0.001453805 | 0.004213136 | 0.001187658 |
| BP | GO:1990928 | response to amino acid starvation | 0.001453805 | 0.004213136 | 0.001187658 |
| BP | GO:0007517 | muscle organ development | 0.001457471 | 0.004218815 | 0.001189259 |
| BP | GO:1902275 | regulation of chromatin organization | 0.001464954 | 0.004235517 | 0.001193967 |
| BP | GO:0030308 | negative regulation of cell growth | 0.001487776 | 0.004296475 | 0.001211151 |
| BP | GO:0031952 | regulation of protein autophosphorylation | 0.001517282 | 0.004361304 | 0.001229426 |
| BP | GO:0045646 | regulation of erythrocyte differentiation | 0.001517282 | 0.004361304 | 0.001229426 |
| BP | GO:0070231 | T cell apoptotic process | 0.001517282 | 0.004361304 | 0.001229426 |
| BP | GO:1900744 | regulation of p38MAPK cascade | 0.001517282 | 0.004361304 | 0.001229426 |
| BP | GO:0002711 | positive regulation of T cell mediated immunity | 0.001582067 | 0.004510806 | 0.001271569 |
| BP | GO:0007595 | lactation | 0.001582067 | 0.004510806 | 0.001271569 |
| BP | GO:0035272 | exocrine system development | 0.001582067 | 0.004510806 | 0.001271569 |
| BP | GO:0046850 | regulation of bone remodeling | 0.001582067 | 0.004510806 | 0.001271569 |
| BP | GO:0055023 | positive regulation of cardiac muscle tissue growth | 0.001582067 | 0.004510806 | 0.001271569 |
| BP | GO:0072604 | interleukin-6 secretion | 0.001582067 | 0.004510806 | 0.001271569 |
| BP | GO:0090199 | regulation of release of cytochrome c from mitochondria | 0.001582067 | 0.004510806 | 0.001271569 |
| BP | GO:0009896 | positive regulation of catabolic process | 0.001634582 | 0.004649812 | 0.001310754 |
| BP | GO:0032386 | regulation of intracellular transport | 0.001634582 | 0.004649812 | 0.001310754 |
| BP | GO:0010712 | regulation of collagen metabolic process | 0.001648155 | 0.00466877 | 0.001316098 |
| BP | GO:0032757 | positive regulation of interleukin-8 production | 0.001648155 | 0.00466877 | 0.001316098 |
| BP | GO:1904707 | positive regulation of vascular smooth muscle cell proliferation | 0.001648155 | 0.00466877 | 0.001316098 |
| BP | GO:0006417 | regulation of translation | 0.001648801 | 0.00466877 | 0.001316098 |
| BP | GO:0010718 | positive regulation of epithelial to mesenchymal transition | 0.001715544 | 0.004835603 | 0.001363128 |
| BP | GO:0032873 | negative regulation of stress-activated MAPK cascade | 0.001715544 | 0.004835603 | 0.001363128 |
| BP | GO:0060425 | lung morphogenesis | 0.001715544 | 0.004835603 | 0.001363128 |
| BP | GO:0070303 | negative regulation of stress-activated protein kinase signaling cascade | 0.001715544 | 0.004835603 | 0.001363128 |
| BP | GO:0046890 | regulation of lipid biosynthetic process | 0.001779274 | 0.005009529 | 0.001412156 |
| BP | GO:0045599 | negative regulation of fat cell differentiation | 0.00178423 | 0.005017767 | 0.001414479 |
| BP | GO:0007179 | transforming growth factor beta receptor signaling pathway | 0.00180506 | 0.005064822 | 0.001427743 |
| BP | GO:0034341 | response to interferon-gamma | 0.00180506 | 0.005064822 | 0.001427743 |
| BP | GO:0050804 | modulation of chemical synaptic transmission | 0.001826249 | 0.005118465 | 0.001442865 |
| BP | GO:0099177 | regulation of trans-synaptic signaling | 0.001841613 | 0.005155682 | 0.001453356 |
| BP | GO:0003179 | heart valve morphogenesis | 0.001854212 | 0.005173376 | 0.001458344 |
| BP | GO:0060421 | positive regulation of heart growth | 0.001854212 | 0.005173376 | 0.001458344 |
| BP | GO:0072132 | mesenchyme morphogenesis | 0.001854212 | 0.005173376 | 0.001458344 |
| BP | GO:0002703 | regulation of leukocyte mediated immunity | 0.001857333 | 0.005176242 | 0.001459152 |
| BP | GO:0050870 | positive regulation of T cell activation | 0.001883822 | 0.005244152 | 0.001478295 |
| BP | GO:0000186 | activation of MAPKK activity | 0.001925485 | 0.005330121 | 0.001502529 |
| BP | GO:0032964 | collagen biosynthetic process | 0.001925485 | 0.005330121 | 0.001502529 |
| BP | GO:0043331 | response to dsRNA | 0.001925485 | 0.005330121 | 0.001502529 |
| BP | GO:0045661 | regulation of myoblast differentiation | 0.001925485 | 0.005330121 | 0.001502529 |
| BP | GO:0045840 | positive regulation of mitotic nuclear division | 0.001925485 | 0.005330121 | 0.001502529 |
| BP | GO:0032655 | regulation of interleukin-12 production | 0.001998046 | 0.005512467 | 0.001553932 |
| BP | GO:0043392 | negative regulation of DNA binding | 0.001998046 | 0.005512467 | 0.001553932 |
| BP | GO:0097345 | mitochondrial outer membrane permeabilization | 0.001998046 | 0.005512467 | 0.001553932 |
| BP | GO:0050670 | regulation of lymphocyte proliferation | 0.002047735 | 0.005636973 | 0.001589029 |
| BP | GO:0090276 | regulation of peptide hormone secretion | 0.002047735 | 0.005636973 | 0.001589029 |
| BP | GO:0001541 | ovarian follicle development | 0.002071894 | 0.00568284 | 0.001601959 |
| BP | GO:0010823 | negative regulation of mitochondrion organization | 0.002071894 | 0.00568284 | 0.001601959 |
| BP | GO:0042306 | regulation of protein import into nucleus | 0.002071894 | 0.00568284 | 0.001601959 |
| BP | GO:0032944 | regulation of mononuclear cell proliferation | 0.002075892 | 0.00568284 | 0.001601959 |
| BP | GO:0051701 | interaction with host | 0.002075892 | 0.00568284 | 0.001601959 |
| BP | GO:0043523 | regulation of neuron apoptotic process | 0.002104289 | 0.005747848 | 0.001620284 |
| BP | GO:0050707 | regulation of cytokine secretion | 0.002104289 | 0.005747848 | 0.001620284 |
| BP | GO:0006977 | DNA damage response, signal transduction by p53 class mediator resulting in cell cycle arrest | 0.002147024 | 0.005851646 | 0.001649544 |
| BP | GO:0032615 | interleukin-12 production | 0.002147024 | 0.005851646 | 0.001649544 |
| BP | GO:0045732 | positive regulation of protein catabolic process | 0.002220306 | 0.006020074 | 0.001697023 |
| BP | GO:2001020 | regulation of response to DNA damage stimulus | 0.002220306 | 0.006020074 | 0.001697023 |
| BP | GO:0060043 | regulation of cardiac muscle cell proliferation | 0.002223434 | 0.006020074 | 0.001697023 |
| BP | GO:0071398 | cellular response to fatty acid | 0.002223434 | 0.006020074 | 0.001697023 |
| BP | GO:0072431 | signal transduction involved in mitotic G1 DNA damage checkpoint | 0.002223434 | 0.006020074 | 0.001697023 |
| BP | GO:1902400 | intracellular signal transduction involved in G1 DNA damage checkpoint | 0.002223434 | 0.006020074 | 0.001697023 |
| BP | GO:0070374 | positive regulation of ERK1 and ERK2 cascade | 0.002249921 | 0.006085126 | 0.001715361 |
| BP | GO:0023061 | signal release | 0.002255729 | 0.006094165 | 0.001717909 |
| BP | GO:0019229 | regulation of vasoconstriction | 0.00230112 | 0.006189738 | 0.00174485 |
| BP | GO:1902041 | regulation of extrinsic apoptotic signaling pathway via death domain receptors | 0.00230112 | 0.006189738 | 0.00174485 |
| BP | GO:1903749 | positive regulation of establishment of protein localization to mitochondrion | 0.00230112 | 0.006189738 | 0.00174485 |
| BP | GO:1904589 | regulation of protein import | 0.00230112 | 0.006189738 | 0.00174485 |
| BP | GO:0019722 | calcium-mediated signaling | 0.002340243 | 0.006288131 | 0.001772587 |
| BP | GO:0001658 | branching involved in ureteric bud morphogenesis | 0.00238008 | 0.006340041 | 0.00178722 |
| BP | GO:0010518 | positive regulation of phospholipase activity | 0.00238008 | 0.006340041 | 0.00178722 |
| BP | GO:0032890 | regulation of organic acid transport | 0.00238008 | 0.006340041 | 0.00178722 |
| BP | GO:0043388 | positive regulation of DNA binding | 0.00238008 | 0.006340041 | 0.00178722 |
| BP | GO:0072413 | signal transduction involved in mitotic cell cycle checkpoint | 0.00238008 | 0.006340041 | 0.00178722 |
| BP | GO:0097755 | positive regulation of blood vessel diameter | 0.00238008 | 0.006340041 | 0.00178722 |
| BP | GO:1902402 | signal transduction involved in mitotic DNA damage checkpoint | 0.00238008 | 0.006340041 | 0.00178722 |
| BP | GO:1902403 | signal transduction involved in mitotic DNA integrity checkpoint | 0.00238008 | 0.006340041 | 0.00178722 |
| BP | GO:1902110 | positive regulation of mitochondrial membrane permeability involved in apoptotic process | 0.002460311 | 0.006546706 | 0.001845477 |
| BP | GO:0070663 | regulation of leukocyte proliferation | 0.002464145 | 0.006549856 | 0.001846365 |
| BP | GO:0003170 | heart valve development | 0.00254181 | 0.006720165 | 0.001894375 |
| BP | GO:0007405 | neuroblast proliferation | 0.00254181 | 0.006720165 | 0.001894375 |
| BP | GO:0010803 | regulation of tumor necrosis factor-mediated signaling pathway | 0.00254181 | 0.006720165 | 0.001894375 |
| BP | GO:0032370 | positive regulation of lipid transport | 0.00254181 | 0.006720165 | 0.001894375 |
| BP | GO:0034113 | heterotypic cell-cell adhesion | 0.00254181 | 0.006720165 | 0.001894375 |
| BP | GO:0001649 | osteoblast differentiation | 0.002559701 | 0.006760236 | 0.00190567 |
| BP | GO:0045453 | bone resorption | 0.002624574 | 0.006909421 | 0.001947725 |
| BP | GO:0055025 | positive regulation of cardiac muscle tissue development | 0.002624574 | 0.006909421 | 0.001947725 |
| BP | GO:1902686 | mitochondrial outer membrane permeabilization involved in programmed cell death | 0.002624574 | 0.006909421 | 0.001947725 |
| BP | GO:0034248 | regulation of cellular amide metabolic process | 0.002650302 | 0.006969731 | 0.001964726 |
| BP | GO:0051249 | regulation of lymphocyte activation | 0.002690208 | 0.007067156 | 0.001992189 |
| BP | GO:0002637 | regulation of immunoglobulin production | 0.002708599 | 0.007085351 | 0.001997318 |
| BP | GO:0032371 | regulation of sterol transport | 0.002708599 | 0.007085351 | 0.001997318 |
| BP | GO:0032374 | regulation of cholesterol transport | 0.002708599 | 0.007085351 | 0.001997318 |
| BP | GO:0034394 | protein localization to cell surface | 0.002708599 | 0.007085351 | 0.001997318 |
| BP | GO:0042445 | hormone metabolic process | 0.002791546 | 0.007285316 | 0.002053687 |
| BP | GO:0035794 | positive regulation of mitochondrial membrane permeability | 0.002793883 | 0.007285316 | 0.002053687 |
| BP | GO:0045670 | regulation of osteoclast differentiation | 0.002793883 | 0.007285316 | 0.002053687 |
| BP | GO:0048738 | cardiac muscle tissue development | 0.002825692 | 0.007360496 | 0.00207488 |
| BP | GO:0032729 | positive regulation of interferon-gamma production | 0.002880424 | 0.007487284 | 0.002110621 |
| BP | GO:0060675 | ureteric bud morphogenesis | 0.002880424 | 0.007487284 | 0.002110621 |
| BP | GO:0009755 | hormone-mediated signaling pathway | 0.002894759 | 0.007516643 | 0.002118897 |
| BP | GO:0046626 | regulation of insulin receptor signaling pathway | 0.002968217 | 0.007667118 | 0.002161315 |
| BP | GO:0051785 | positive regulation of nuclear division | 0.002968217 | 0.007667118 | 0.002161315 |
| BP | GO:0072171 | mesonephric tubule morphogenesis | 0.002968217 | 0.007667118 | 0.002161315 |
| BP | GO:1902108 | regulation of mitochondrial membrane permeability involved in apoptotic process | 0.002968217 | 0.007667118 | 0.002161315 |
| BP | GO:1905710 | positive regulation of membrane permeability | 0.002968217 | 0.007667118 | 0.002161315 |
| BP | GO:0030258 | lipid modification | 0.003000308 | 0.007741922 | 0.002182402 |
| BP | GO:0048662 | negative regulation of smooth muscle cell proliferation | 0.00305726 | 0.007864253 | 0.002216886 |
| BP | GO:0060038 | cardiac muscle cell proliferation | 0.00305726 | 0.007864253 | 0.002216886 |
| BP | GO:2000573 | positive regulation of DNA biosynthetic process | 0.00305726 | 0.007864253 | 0.002216886 |
| BP | GO:0050663 | cytokine secretion | 0.003071981 | 0.007893905 | 0.002225245 |
| BP | GO:0035914 | skeletal muscle cell differentiation | 0.00314755 | 0.008062948 | 0.002272897 |
| BP | GO:0050918 | positive chemotaxis | 0.00314755 | 0.008062948 | 0.002272897 |
| BP | GO:0071230 | cellular response to amino acid stimulus | 0.00314755 | 0.008062948 | 0.002272897 |
| BP | GO:0015909 | long-chain fatty acid transport | 0.003239085 | 0.008280267 | 0.002334158 |
| BP | GO:0070227 | lymphocyte apoptotic process | 0.003239085 | 0.008280267 | 0.002334158 |
| BP | GO:0002709 | regulation of T cell mediated immunity | 0.003331861 | 0.008473621 | 0.002388663 |
| BP | GO:0010517 | regulation of phospholipase activity | 0.003331861 | 0.008473621 | 0.002388663 |
| BP | GO:0051966 | regulation of synaptic transmission, glutamatergic | 0.003331861 | 0.008473621 | 0.002388663 |
| BP | GO:0060395 | SMAD protein signal transduction | 0.003331861 | 0.008473621 | 0.002388663 |
| BP | GO:0072091 | regulation of stem cell proliferation | 0.003331861 | 0.008473621 | 0.002388663 |
| BP | GO:0042180 | cellular ketone metabolic process | 0.003369239 | 0.008551087 | 0.002410501 |
| BP | GO:0065004 | protein-DNA complex assembly | 0.003369239 | 0.008551087 | 0.002410501 |
| BP | GO:0045926 | negative regulation of growth | 0.003407596 | 0.008630713 | 0.002432947 |
| BP | GO:0071560 | cellular response to transforming growth factor beta stimulus | 0.003407596 | 0.008630713 | 0.002432947 |
| BP | GO:0042531 | positive regulation of tyrosine phosphorylation of STAT protein | 0.003425875 | 0.008668129 | 0.002443494 |
| BP | GO:0006305 | DNA alkylation | 0.003521124 | 0.008872802 | 0.00250119 |
| BP | GO:0006306 | DNA methylation | 0.003521124 | 0.008872802 | 0.00250119 |
| BP | GO:0060193 | positive regulation of lipase activity | 0.003521124 | 0.008872802 | 0.00250119 |
| BP | GO:1903747 | regulation of establishment of protein localization to mitochondrion | 0.003521124 | 0.008872802 | 0.00250119 |
| BP | GO:0050688 | regulation of defense response to virus | 0.003617606 | 0.009078906 | 0.00255929 |
| BP | GO:0072401 | signal transduction involved in DNA integrity checkpoint | 0.003617606 | 0.009078906 | 0.00255929 |
| BP | GO:0072422 | signal transduction involved in DNA damage checkpoint | 0.003617606 | 0.009078906 | 0.00255929 |
| BP | GO:1903524 | positive regulation of blood circulation | 0.003617606 | 0.009078906 | 0.00255929 |
| BP | GO:0071559 | response to transforming growth factor beta | 0.003643397 | 0.009134359 | 0.002574921 |
| BP | GO:0032677 | regulation of interleukin-8 production | 0.003715318 | 0.009277036 | 0.002615141 |
| BP | GO:0072078 | nephron tubule morphogenesis | 0.003715318 | 0.009277036 | 0.002615141 |
| BP | GO:0072395 | signal transduction involved in cell cycle checkpoint | 0.003715318 | 0.009277036 | 0.002615141 |
| BP | GO:1900076 | regulation of cellular response to insulin stimulus | 0.003715318 | 0.009277036 | 0.002615141 |
| BP | GO:0048863 | stem cell differentiation | 0.003724169 | 0.009289754 | 0.002618726 |
| BP | GO:0001960 | negative regulation of cytokine-mediated signaling pathway | 0.003814256 | 0.009495308 | 0.002676671 |
| BP | GO:0007422 | peripheral nervous system development | 0.003814256 | 0.009495308 | 0.002676671 |
| BP | GO:0072659 | protein localization to plasma membrane | 0.00384738 | 0.009568132 | 0.0026972 |
| BP | GO:0042310 | vasoconstriction | 0.003914418 | 0.009695833 | 0.002733198 |
| BP | GO:0046902 | regulation of mitochondrial membrane permeability | 0.003914418 | 0.009695833 | 0.002733198 |
| BP | GO:0060411 | cardiac septum morphogenesis | 0.003914418 | 0.009695833 | 0.002733198 |
| BP | GO:0061045 | negative regulation of wound healing | 0.003914418 | 0.009695833 | 0.002733198 |
| BP | GO:0031330 | negative regulation of cellular catabolic process | 0.004015517 | 0.009907245 | 0.002792793 |
| BP | GO:0016575 | histone deacetylation | 0.004015801 | 0.009907245 | 0.002792793 |
| BP | GO:0032481 | positive regulation of type I interferon production | 0.004015801 | 0.009907245 | 0.002792793 |
| BP | GO:0061418 | regulation of transcription from RNA polymerase II promoter in response to hypoxia | 0.004015801 | 0.009907245 | 0.002792793 |
| BP | GO:0044839 | cell cycle G2/M phase transition | 0.004101248 | 0.010097894 | 0.002846536 |
| BP | GO:0046883 | regulation of hormone secretion | 0.004101248 | 0.010097894 | 0.002846536 |
| BP | GO:0031016 | pancreas development | 0.004118401 | 0.010099889 | 0.002847099 |
| BP | GO:0055021 | regulation of cardiac muscle tissue growth | 0.004118401 | 0.010099889 | 0.002847099 |
| BP | GO:0061333 | renal tubule morphogenesis | 0.004118401 | 0.010099889 | 0.002847099 |
| BP | GO:0072332 | intrinsic apoptotic signaling pathway by p53 class mediator | 0.004118401 | 0.010099889 | 0.002847099 |
| BP | GO:0003151 | outflow tract morphogenesis | 0.004222217 | 0.010344223 | 0.002915975 |
| BP | GO:0045165 | cell fate commitment | 0.00427606 | 0.010465764 | 0.002950237 |
| BP | GO:1901617 | organic hydroxy compound biosynthetic process | 0.004320464 | 0.010563984 | 0.002977924 |
| BP | GO:0014855 | striated muscle cell proliferation | 0.004433483 | 0.010786977 | 0.003040785 |
| BP | GO:0032204 | regulation of telomere maintenance | 0.004433483 | 0.010786977 | 0.003040785 |
| BP | GO:0060761 | negative regulation of response to cytokine stimulus | 0.004433483 | 0.010786977 | 0.003040785 |
| BP | GO:0110110 | positive regulation of animal organ morphogenesis | 0.004433483 | 0.010786977 | 0.003040785 |
| BP | GO:1902930 | regulation of alcohol biosynthetic process | 0.004433483 | 0.010786977 | 0.003040785 |
| BP | GO:0006260 | DNA replication | 0.004455368 | 0.010829566 | 0.00305279 |
| BP | GO:0032637 | interleukin-8 production | 0.004540926 | 0.01099429 | 0.003099225 |
| BP | GO:0034502 | protein localization to chromosome | 0.004540926 | 0.01099429 | 0.003099225 |
| BP | GO:0071158 | positive regulation of cell cycle arrest | 0.004540926 | 0.01099429 | 0.003099225 |
| BP | GO:1905954 | positive regulation of lipid localization | 0.004540926 | 0.01099429 | 0.003099225 |
| BP | GO:0001822 | kidney development | 0.004639206 | 0.011221249 | 0.003163203 |
| BP | GO:0042509 | regulation of tyrosine phosphorylation of STAT protein | 0.004649574 | 0.011224362 | 0.003164081 |
| BP | GO:0071277 | cellular response to calcium ion | 0.004649574 | 0.011224362 | 0.003164081 |
| BP | GO:0010507 | negative regulation of autophagy | 0.004759422 | 0.011433713 | 0.003223096 |
| BP | GO:0043154 | negative regulation of cysteine-type endopeptidase activity involved in apoptotic process | 0.004759422 | 0.011433713 | 0.003223096 |
| BP | GO:0050886 | endocrine process | 0.004759422 | 0.011433713 | 0.003223096 |
| BP | GO:0060420 | regulation of heart growth | 0.004759422 | 0.011433713 | 0.003223096 |
| BP | GO:0097756 | negative regulation of blood vessel diameter | 0.004759422 | 0.011433713 | 0.003223096 |
| BP | GO:0002028 | regulation of sodium ion transport | 0.004870468 | 0.011689123 | 0.003295094 |
| BP | GO:0031647 | regulation of protein stability | 0.004923533 | 0.011805017 | 0.003327764 |
| BP | GO:0015980 | energy derivation by oxidation of organic compounds | 0.004971927 | 0.011866336 | 0.003345049 |
| BP | GO:0001776 | leukocyte homeostasis | 0.004982709 | 0.011866336 | 0.003345049 |
| BP | GO:0007260 | tyrosine phosphorylation of STAT protein | 0.004982709 | 0.011866336 | 0.003345049 |
| BP | GO:0042058 | regulation of epidermal growth factor receptor signaling pathway | 0.004982709 | 0.011866336 | 0.003345049 |
| BP | GO:0042446 | hormone biosynthetic process | 0.004982709 | 0.011866336 | 0.003345049 |
| BP | GO:0070542 | response to fatty acid | 0.004982709 | 0.011866336 | 0.003345049 |
| BP | GO:0090559 | regulation of membrane permeability | 0.004982709 | 0.011866336 | 0.003345049 |
| BP | GO:0006112 | energy reserve metabolic process | 0.005096142 | 0.012089888 | 0.003408068 |
| BP | GO:0045844 | positive regulation of striated muscle tissue development | 0.005096142 | 0.012089888 | 0.003408068 |
| BP | GO:0048636 | positive regulation of muscle organ development | 0.005096142 | 0.012089888 | 0.003408068 |
| BP | GO:0051781 | positive regulation of cell division | 0.005096142 | 0.012089888 | 0.003408068 |
| BP | GO:1900407 | regulation of cellular response to oxidative stress | 0.005210765 | 0.01229104 | 0.003464771 |
| BP | GO:1901863 | positive regulation of muscle tissue development | 0.005210765 | 0.01229104 | 0.003464771 |
| BP | GO:1901888 | regulation of cell junction assembly | 0.005210765 | 0.01229104 | 0.003464771 |
| BP | GO:1903321 | negative regulation of protein modification by small protein conjugation or removal | 0.005210765 | 0.01229104 | 0.003464771 |
| BP | GO:1904035 | regulation of epithelial cell apoptotic process | 0.005210765 | 0.01229104 | 0.003464771 |
| BP | GO:2000177 | regulation of neural precursor cell proliferation | 0.005210765 | 0.01229104 | 0.003464771 |
| BP | GO:0090287 | regulation of cellular response to growth factor stimulus | 0.005318809 | 0.012528344 | 0.003531666 |
| BP | GO:0046427 | positive regulation of JAK-STAT cascade | 0.005326573 | 0.012528344 | 0.003531666 |
| BP | GO:0097306 | cellular response to alcohol | 0.005326573 | 0.012528344 | 0.003531666 |
| BP | GO:0072001 | renal system development | 0.005369529 | 0.012617373 | 0.003556762 |
| BP | GO:0001656 | metanephros development | 0.005443566 | 0.012742893 | 0.003592146 |
| BP | GO:0010717 | regulation of epithelial to mesenchymal transition | 0.005443566 | 0.012742893 | 0.003592146 |
| BP | GO:0046849 | bone remodeling | 0.005443566 | 0.012742893 | 0.003592146 |
| BP | GO:1903035 | negative regulation of response to wounding | 0.005443566 | 0.012742893 | 0.003592146 |
| BP | GO:0045638 | negative regulation of myeloid cell differentiation | 0.005561739 | 0.013007209 | 0.003666655 |
| BP | GO:1903522 | regulation of blood circulation | 0.005575347 | 0.013026708 | 0.003672152 |
| BP | GO:1904894 | positive regulation of STAT cascade | 0.005681091 | 0.013248732 | 0.003734739 |
| BP | GO:2000117 | negative regulation of cysteine-type endopeptidase activity | 0.005681091 | 0.013248732 | 0.003734739 |
| BP | GO:0006476 | protein deacetylation | 0.005801617 | 0.013478945 | 0.003799635 |
| BP | GO:0007589 | body fluid secretion | 0.005801617 | 0.013478945 | 0.003799635 |
| BP | GO:0035249 | synaptic transmission, glutamatergic | 0.005801617 | 0.013478945 | 0.003799635 |
| BP | GO:1901184 | regulation of ERBB signaling pathway | 0.005801617 | 0.013478945 | 0.003799635 |
| BP | GO:0044728 | DNA methylation or demethylation | 0.005923316 | 0.013748767 | 0.003875696 |
| BP | GO:0042102 | positive regulation of T cell proliferation | 0.006046184 | 0.013981448 | 0.003941287 |
| BP | GO:0044070 | regulation of anion transport | 0.006046184 | 0.013981448 | 0.003941287 |
| BP | GO:0045069 | regulation of viral genome replication | 0.006046184 | 0.013981448 | 0.003941287 |
| BP | GO:0050810 | regulation of steroid biosynthetic process | 0.006046184 | 0.013981448 | 0.003941287 |
| BP | GO:0010660 | regulation of muscle cell apoptotic process | 0.00617022 | 0.014254937 | 0.004018382 |
| BP | GO:0001657 | ureteric bud development | 0.006295419 | 0.014503518 | 0.004088456 |
| BP | GO:1902882 | regulation of response to oxidative stress | 0.006295419 | 0.014503518 | 0.004088456 |
| BP | GO:2001022 | positive regulation of response to DNA damage stimulus | 0.006295419 | 0.014503518 | 0.004088456 |
| BP | GO:1990778 | protein localization to cell periphery | 0.00633307 | 0.014576676 | 0.004109078 |
| BP | GO:0002042 | cell migration involved in sprouting angiogenesis | 0.006421779 | 0.014726008 | 0.004151174 |
| BP | GO:0060191 | regulation of lipase activity | 0.006421779 | 0.014726008 | 0.004151174 |
| BP | GO:0072163 | mesonephric epithelium development | 0.006421779 | 0.014726008 | 0.004151174 |
| BP | GO:0072164 | mesonephric tubule development | 0.006421779 | 0.014726008 | 0.004151174 |
| BP | GO:0043010 | camera-type eye development | 0.006503084 | 0.014884836 | 0.004195947 |
| BP | GO:0050863 | regulation of T cell activation | 0.006503084 | 0.014884836 | 0.004195947 |
| BP | GO:0070301 | cellular response to hydrogen peroxide | 0.006549297 | 0.014976746 | 0.004221856 |
| BP | GO:0010657 | muscle cell apoptotic process | 0.006677971 | 0.015228732 | 0.004292889 |
| BP | GO:0030301 | cholesterol transport | 0.006677971 | 0.015228732 | 0.004292889 |
| BP | GO:0055024 | regulation of cardiac muscle tissue development | 0.006677971 | 0.015228732 | 0.004292889 |
| BP | GO:0032611 | interleukin-1 beta production | 0.006807798 | 0.015481947 | 0.004364269 |
| BP | GO:0032649 | regulation of interferon-gamma production | 0.006807798 | 0.015481947 | 0.004364269 |
| BP | GO:0050830 | defense response to Gram-positive bacterium | 0.006807798 | 0.015481947 | 0.004364269 |
| BP | GO:0001823 | mesonephros development | 0.006938775 | 0.015750828 | 0.004440065 |
| BP | GO:0098869 | cellular oxidant detoxification | 0.006938775 | 0.015750828 | 0.004440065 |
| BP | GO:0035601 | protein deacylation | 0.007070899 | 0.01603602 | 0.004520459 |
| BP | GO:0030593 | neutrophil chemotaxis | 0.007204167 | 0.016308334 | 0.004597223 |
| BP | GO:0098732 | macromolecule deacylation | 0.007204167 | 0.016308334 | 0.004597223 |
| BP | GO:0019058 | viral life cycle | 0.007332691 | 0.016567089 | 0.004670164 |
| BP | GO:0045185 | maintenance of protein location | 0.007338577 | 0.016567089 | 0.004670164 |
| BP | GO:0055017 | cardiac muscle tissue growth | 0.007338577 | 0.016567089 | 0.004670164 |
| BP | GO:0002286 | T cell activation involved in immune response | 0.007474126 | 0.016842333 | 0.004747754 |
| BP | GO:0002456 | T cell mediated immunity | 0.007474126 | 0.016842333 | 0.004747754 |
| BP | GO:0008202 | steroid metabolic process | 0.007518294 | 0.016926432 | 0.004771461 |
| BP | GO:0090150 | establishment of protein localization to membrane | 0.007580781 | 0.017051583 | 0.00480674 |
| BP | GO:0030330 | DNA damage response, signal transduction by p53 class mediator | 0.007610811 | 0.017088034 | 0.004817015 |
| BP | GO:0062014 | negative regulation of small molecule metabolic process | 0.007610811 | 0.017088034 | 0.004817015 |
| BP | GO:0015849 | organic acid transport | 0.007643578 | 0.017114967 | 0.004824607 |
| BP | GO:0016042 | lipid catabolic process | 0.007643578 | 0.017114967 | 0.004824607 |
| BP | GO:0046942 | carboxylic acid transport | 0.007643578 | 0.017114967 | 0.004824607 |
| BP | GO:0007596 | blood coagulation | 0.007833833 | 0.0175251 | 0.004940221 |
| BP | GO:0007599 | hemostasis | 0.008157164 | 0.018231925 | 0.005139471 |
| BP | GO:0031623 | receptor internalization | 0.008168858 | 0.018241569 | 0.00514219 |
| BP | GO:0050817 | coagulation | 0.008222769 | 0.018345384 | 0.005171455 |
| BP | GO:0003279 | cardiac septum development | 0.008311183 | 0.018426228 | 0.005194244 |
| BP | GO:0015918 | sterol transport | 0.008311183 | 0.018426228 | 0.005194244 |
| BP | GO:0043279 | response to alkaloid | 0.008311183 | 0.018426228 | 0.005194244 |
| BP | GO:0051817 | modification of morphology or physiology of other organism involved in symbiotic interaction | 0.008311183 | 0.018426228 | 0.005194244 |
| BP | GO:0060419 | heart growth | 0.008311183 | 0.018426228 | 0.005194244 |
| BP | GO:1903510 | mucopolysaccharide metabolic process | 0.008311183 | 0.018426228 | 0.005194244 |
| BP | GO:1990748 | cellular detoxification | 0.008311183 | 0.018426228 | 0.005194244 |
| BP | GO:0032609 | interferon-gamma production | 0.008454627 | 0.018727454 | 0.005279158 |
| BP | GO:0042752 | regulation of circadian rhythm | 0.008599188 | 0.019030612 | 0.005364616 |
| BP | GO:0032612 | interleukin-1 production | 0.008744864 | 0.019318413 | 0.005445746 |
| BP | GO:0032963 | collagen metabolic process | 0.008744864 | 0.019318413 | 0.005445746 |
| BP | GO:0006304 | DNA modification | 0.008891651 | 0.01961755 | 0.005530071 |
| BP | GO:0002449 | lymphocyte mediated immunity | 0.008896146 | 0.01961755 | 0.005530071 |
| BP | GO:0010822 | positive regulation of mitochondrion organization | 0.009039547 | 0.01988057 | 0.005604214 |
| BP | GO:0010906 | regulation of glucose metabolic process | 0.009039547 | 0.01988057 | 0.005604214 |
| BP | GO:1900371 | regulation of purine nucleotide biosynthetic process | 0.009039547 | 0.01988057 | 0.005604214 |
| BP | GO:0030808 | regulation of nucleotide biosynthetic process | 0.009188549 | 0.020172374 | 0.005686472 |
| BP | GO:1990266 | neutrophil migration | 0.009188549 | 0.020172374 | 0.005686472 |
| BP | GO:0007173 | epidermal growth factor receptor signaling pathway | 0.009338655 | 0.020483721 | 0.005774239 |
| BP | GO:0072089 | stem cell proliferation | 0.009489861 | 0.020796929 | 0.005862531 |
| BP | GO:0002460 | adaptive immune response based on somatic recombination of immune receptors built from immunoglobulin superfamily domains | 0.009529313 | 0.020864891 | 0.005881689 |
| BP | GO:0001654 | eye development | 0.009601262 | 0.021003823 | 0.005920853 |
| BP | GO:0030968 | endoplasmic reticulum unfolded protein response | 0.009642165 | 0.021074652 | 0.005940819 |
| BP | GO:0030111 | regulation of Wnt signaling pathway | 0.009673532 | 0.021105889 | 0.005949625 |
| BP | GO:0045861 | negative regulation of proteolysis | 0.009673532 | 0.021105889 | 0.005949625 |
| BP | GO:0019079 | viral genome replication | 0.009795564 | 0.021334481 | 0.006014063 |
| BP | GO:0043500 | muscle adaptation | 0.009795564 | 0.021334481 | 0.006014063 |
| BP | GO:0150063 | visual system development | 0.00989227 | 0.021526136 | 0.00606809 |
| BP | GO:0002062 | chondrocyte differentiation | 0.009950057 | 0.021632841 | 0.006098169 |
| BP | GO:0030198 | extracellular matrix organization | 0.010039704 | 0.021808566 | 0.006147705 |
| BP | GO:0019218 | regulation of steroid metabolic process | 0.010105639 | 0.021932519 | 0.006182647 |
| BP | GO:0045471 | response to ethanol | 0.010262308 | 0.022235596 | 0.006268082 |
| BP | GO:0048880 | sensory system development | 0.010263275 | 0.022235596 | 0.006268082 |
| BP | GO:0018107 | peptidyl-threonine phosphorylation | 0.010420062 | 0.022516079 | 0.006347149 |
| BP | GO:0032479 | regulation of type I interferon production | 0.010420062 | 0.022516079 | 0.006347149 |
| BP | GO:0045667 | regulation of osteoblast differentiation | 0.010420062 | 0.022516079 | 0.006347149 |
| BP | GO:0032606 | type I interferon production | 0.010738814 | 0.023184583 | 0.006535596 |
| BP | GO:0003206 | cardiac chamber morphogenesis | 0.010899806 | 0.023511623 | 0.006627787 |
| BP | GO:0098754 | detoxification | 0.011225009 | 0.024191998 | 0.006819581 |
| BP | GO:0030518 | intracellular steroid hormone receptor signaling pathway | 0.011389215 | 0.024503167 | 0.006907297 |
| BP | GO:0042177 | negative regulation of protein catabolic process | 0.011389215 | 0.024503167 | 0.006907297 |
| BP | GO:0009165 | nucleotide biosynthetic process | 0.011424946 | 0.024558666 | 0.006922942 |
| BP | GO:0002920 | regulation of humoral immune response | 0.011720822 | 0.025020616 | 0.007053163 |
| BP | GO:0006766 | vitamin metabolic process | 0.011720822 | 0.025020616 | 0.007053163 |
| BP | GO:0007006 | mitochondrial membrane organization | 0.011720822 | 0.025020616 | 0.007053163 |
| BP | GO:0018210 | peptidyl-threonine modification | 0.011720822 | 0.025020616 | 0.007053163 |
| BP | GO:0030260 | entry into host cell | 0.011720822 | 0.025020616 | 0.007053163 |
| BP | GO:0044409 | entry into host | 0.011720822 | 0.025020616 | 0.007053163 |
| BP | GO:0051806 | entry into cell of other organism involved in symbiotic interaction | 0.011720822 | 0.025020616 | 0.007053163 |
| BP | GO:0051828 | entry into other organism involved in symbiotic interaction | 0.011720822 | 0.025020616 | 0.007053163 |
| BP | GO:1901293 | nucleoside phosphate biosynthetic process | 0.011747135 | 0.025055149 | 0.007062898 |
| BP | GO:0060359 | response to ammonium ion | 0.011888219 | 0.025334204 | 0.007141561 |
| BP | GO:0034605 | cellular response to heat | 0.012226183 | 0.025476978 | 0.007181809 |
| BP | GO:0046425 | regulation of JAK-STAT cascade | 0.012226183 | 0.025476978 | 0.007181809 |
| BP | GO:0010918 | positive regulation of mitochondrial membrane potential | 0.012254097 | 0.025476978 | 0.007181809 |
| BP | GO:0031284 | positive regulation of guanylate cyclase activity | 0.012254097 | 0.025476978 | 0.007181809 |
| BP | GO:0032025 | response to cobalt ion | 0.012254097 | 0.025476978 | 0.007181809 |
| BP | GO:0032070 | regulation of deoxyribonuclease activity | 0.012254097 | 0.025476978 | 0.007181809 |
| BP | GO:0032308 | positive regulation of prostaglandin secretion | 0.012254097 | 0.025476978 | 0.007181809 |
| BP | GO:0034085 | establishment of sister chromatid cohesion | 0.012254097 | 0.025476978 | 0.007181809 |
| BP | GO:0044359 | modulation of molecular function in other organism | 0.012254097 | 0.025476978 | 0.007181809 |
| BP | GO:0045657 | positive regulation of monocyte differentiation | 0.012254097 | 0.025476978 | 0.007181809 |
| BP | GO:0045713 | low-density lipoprotein particle receptor biosynthetic process | 0.012254097 | 0.025476978 | 0.007181809 |
| BP | GO:0045792 | negative regulation of cell size | 0.012254097 | 0.025476978 | 0.007181809 |
| BP | GO:0046322 | negative regulation of fatty acid oxidation | 0.012254097 | 0.025476978 | 0.007181809 |
| BP | GO:0052205 | modulation of molecular function in other organism involved in symbiotic interaction | 0.012254097 | 0.025476978 | 0.007181809 |
| BP | GO:0060068 | vagina development | 0.012254097 | 0.025476978 | 0.007181809 |
| BP | GO:0070091 | glucagon secretion | 0.012254097 | 0.025476978 | 0.007181809 |
| BP | GO:0071104 | response to interleukin-9 | 0.012254097 | 0.025476978 | 0.007181809 |
| BP | GO:0072610 | interleukin-12 secretion | 0.012254097 | 0.025476978 | 0.007181809 |
| BP | GO:0090037 | positive regulation of protein kinase C signaling | 0.012254097 | 0.025476978 | 0.007181809 |
| BP | GO:0090154 | positive regulation of sphingolipid biosynthetic process | 0.012254097 | 0.025476978 | 0.007181809 |
| BP | GO:0090336 | positive regulation of brown fat cell differentiation | 0.012254097 | 0.025476978 | 0.007181809 |
| BP | GO:0097050 | type B pancreatic cell apoptotic process | 0.012254097 | 0.025476978 | 0.007181809 |
| BP | GO:0140052 | cellular response to oxidised low-density lipoprotein particle stimulus | 0.012254097 | 0.025476978 | 0.007181809 |
| BP | GO:1900222 | negative regulation of amyloid-beta clearance | 0.012254097 | 0.025476978 | 0.007181809 |
| BP | GO:1901033 | positive regulation of response to reactive oxygen species | 0.012254097 | 0.025476978 | 0.007181809 |
| BP | GO:1901725 | regulation of histone deacetylase activity | 0.012254097 | 0.025476978 | 0.007181809 |
| BP | GO:1902101 | positive regulation of metaphase/anaphase transition of cell cycle | 0.012254097 | 0.025476978 | 0.007181809 |
| BP | GO:2000304 | positive regulation of ceramide biosynthetic process | 0.012254097 | 0.025476978 | 0.007181809 |
| BP | GO:2000628 | regulation of miRNA metabolic process | 0.012254097 | 0.025476978 | 0.007181809 |
| BP | GO:0008584 | male gonad development | 0.012396746 | 0.025751895 | 0.007259306 |
| BP | GO:0002831 | regulation of response to biotic stimulus | 0.012568359 | 0.026064583 | 0.007347451 |
| BP | GO:0046546 | development of primary male sexual characteristics | 0.012568359 | 0.026064583 | 0.007347451 |
| BP | GO:0001837 | epithelial to mesenchymal transition | 0.01274102 | 0.026356318 | 0.007429689 |
| BP | GO:0034620 | cellular response to unfolded protein | 0.01274102 | 0.026356318 | 0.007429689 |
| BP | GO:0046330 | positive regulation of JNK cascade | 0.01274102 | 0.026356318 | 0.007429689 |
| BP | GO:0050709 | negative regulation of protein secretion | 0.012914725 | 0.026693312 | 0.007524686 |
| BP | GO:0010675 | regulation of cellular carbohydrate metabolic process | 0.013089474 | 0.026986805 | 0.00760742 |
| BP | GO:0050871 | positive regulation of B cell activation | 0.013089474 | 0.026986805 | 0.00760742 |
| BP | GO:1900542 | regulation of purine nucleotide metabolic process | 0.013089474 | 0.026986805 | 0.00760742 |
| BP | GO:0044344 | cellular response to fibroblast growth factor stimulus | 0.013265262 | 0.027140778 | 0.007650824 |
| BP | GO:0106106 | cold-induced thermogenesis | 0.013265262 | 0.027140778 | 0.007650824 |
| BP | GO:0120161 | regulation of cold-induced thermogenesis | 0.013265262 | 0.027140778 | 0.007650824 |
| BP | GO:0000185 | activation of MAPKKK activity | 0.013471575 | 0.027140778 | 0.007650824 |
| BP | GO:0002923 | regulation of humoral immune response mediated by circulating immunoglobulin | 0.013471575 | 0.027140778 | 0.007650824 |
| BP | GO:0009629 | response to gravity | 0.013471575 | 0.027140778 | 0.007650824 |
| BP | GO:0032306 | regulation of prostaglandin secretion | 0.013471575 | 0.027140778 | 0.007650824 |
| BP | GO:0033234 | negative regulation of protein sumoylation | 0.013471575 | 0.027140778 | 0.007650824 |
| BP | GO:0033327 | Leydig cell differentiation | 0.013471575 | 0.027140778 | 0.007650824 |
| BP | GO:0036462 | TRAIL-activated apoptotic signaling pathway | 0.013471575 | 0.027140778 | 0.007650824 |
| BP | GO:0043117 | positive regulation of vascular permeability | 0.013471575 | 0.027140778 | 0.007650824 |
| BP | GO:0043129 | surfactant homeostasis | 0.013471575 | 0.027140778 | 0.007650824 |
| BP | GO:0051024 | positive regulation of immunoglobulin secretion | 0.013471575 | 0.027140778 | 0.007650824 |
| BP | GO:0060439 | trachea morphogenesis | 0.013471575 | 0.027140778 | 0.007650824 |
| BP | GO:0060525 | prostate glandular acinus development | 0.013471575 | 0.027140778 | 0.007650824 |
| BP | GO:0060736 | prostate gland growth | 0.013471575 | 0.027140778 | 0.007650824 |
| BP | GO:0060947 | cardiac vascular smooth muscle cell differentiation | 0.013471575 | 0.027140778 | 0.007650824 |
| BP | GO:0061307 | cardiac neural crest cell differentiation involved in heart development | 0.013471575 | 0.027140778 | 0.007650824 |
| BP | GO:0061308 | cardiac neural crest cell development involved in heart development | 0.013471575 | 0.027140778 | 0.007650824 |
| BP | GO:0070106 | interleukin-27-mediated signaling pathway | 0.013471575 | 0.027140778 | 0.007650824 |
| BP | GO:0070673 | response to interleukin-18 | 0.013471575 | 0.027140778 | 0.007650824 |
| BP | GO:0070757 | interleukin-35-mediated signaling pathway | 0.013471575 | 0.027140778 | 0.007650824 |
| BP | GO:0071803 | positive regulation of podosome assembly | 0.013471575 | 0.027140778 | 0.007650824 |
| BP | GO:0072584 | caveolin-mediated endocytosis | 0.013471575 | 0.027140778 | 0.007650824 |
| BP | GO:1905820 | positive regulation of chromosome separation | 0.013471575 | 0.027140778 | 0.007650824 |
| BP | GO:1990440 | positive regulation of transcription from RNA polymerase II promoter in response to endoplasmic reticulum stress | 0.013471575 | 0.027140778 | 0.007650824 |
| BP | GO:2000551 | regulation of T-helper 2 cell cytokine production | 0.013471575 | 0.027140778 | 0.007650824 |
| BP | GO:2001269 | positive regulation of cysteine-type endopeptidase activity involved in apoptotic signaling pathway | 0.013471575 | 0.027140778 | 0.007650824 |
| BP | GO:0007612 | learning | 0.013619948 | 0.027417354 | 0.007728789 |
| BP | GO:0006140 | regulation of nucleotide metabolic process | 0.01379884 | 0.027732303 | 0.007817571 |
| BP | GO:1904892 | regulation of STAT cascade | 0.01379884 | 0.027732303 | 0.007817571 |
| BP | GO:0061351 | neural precursor cell proliferation | 0.013978762 | 0.02807108 | 0.007913071 |
| BP | GO:0002792 | negative regulation of peptide secretion | 0.014159711 | 0.028388325 | 0.0080025 |
| BP | GO:0061041 | regulation of wound healing | 0.014159711 | 0.028388325 | 0.0080025 |
| BP | GO:0043588 | skin development | 0.014241188 | 0.028528539 | 0.008042025 |
| BP | GO:1903900 | regulation of viral life cycle | 0.014341684 | 0.028566321 | 0.008052676 |
| BP | GO:0043062 | extracellular structure organization | 0.014515188 | 0.028566321 | 0.008052676 |
| BP | GO:0050777 | negative regulation of immune response | 0.014524679 | 0.028566321 | 0.008052676 |
| BP | GO:0071774 | response to fibroblast growth factor | 0.014524679 | 0.028566321 | 0.008052676 |
| BP | GO:0002551 | mast cell chemotaxis | 0.014687619 | 0.028566321 | 0.008052676 |
| BP | GO:0002674 | negative regulation of acute inflammatory response | 0.014687619 | 0.028566321 | 0.008052676 |
| BP | GO:0002863 | positive regulation of inflammatory response to antigenic stimulus | 0.014687619 | 0.028566321 | 0.008052676 |
| BP | GO:0003337 | mesenchymal to epithelial transition involved in metanephros morphogenesis | 0.014687619 | 0.028566321 | 0.008052676 |
| BP | GO:0006527 | arginine catabolic process | 0.014687619 | 0.028566321 | 0.008052676 |
| BP | GO:0006983 | ER overload response | 0.014687619 | 0.028566321 | 0.008052676 |
| BP | GO:0010623 | programmed cell death involved in cell development | 0.014687619 | 0.028566321 | 0.008052676 |
| BP | GO:0010889 | regulation of sequestering of triglyceride | 0.014687619 | 0.028566321 | 0.008052676 |
| BP | GO:0010960 | magnesium ion homeostasis | 0.014687619 | 0.028566321 | 0.008052676 |
| BP | GO:0031282 | regulation of guanylate cyclase activity | 0.014687619 | 0.028566321 | 0.008052676 |
| BP | GO:0031392 | regulation of prostaglandin biosynthetic process | 0.014687619 | 0.028566321 | 0.008052676 |
| BP | GO:0032429 | regulation of phospholipase A2 activity | 0.014687619 | 0.028566321 | 0.008052676 |
| BP | GO:0034086 | maintenance of sister chromatid cohesion | 0.014687619 | 0.028566321 | 0.008052676 |
| BP | GO:0034088 | maintenance of mitotic sister chromatid cohesion | 0.014687619 | 0.028566321 | 0.008052676 |
| BP | GO:0043922 | negative regulation by host of viral transcription | 0.014687619 | 0.028566321 | 0.008052676 |
| BP | GO:0045416 | positive regulation of interleukin-8 biosynthetic process | 0.014687619 | 0.028566321 | 0.008052676 |
| BP | GO:0051095 | regulation of helicase activity | 0.014687619 | 0.028566321 | 0.008052676 |
| BP | GO:0051798 | positive regulation of hair follicle development | 0.014687619 | 0.028566321 | 0.008052676 |
| BP | GO:0060442 | branching involved in prostate gland morphogenesis | 0.014687619 | 0.028566321 | 0.008052676 |
| BP | GO:0070243 | regulation of thymocyte apoptotic process | 0.014687619 | 0.028566321 | 0.008052676 |
| BP | GO:0070486 | leukocyte aggregation | 0.014687619 | 0.028566321 | 0.008052676 |
| BP | GO:0071639 | positive regulation of monocyte chemotactic protein-1 production | 0.014687619 | 0.028566321 | 0.008052676 |
| BP | GO:0071679 | commissural neuron axon guidance | 0.014687619 | 0.028566321 | 0.008052676 |
| BP | GO:0072540 | T-helper 17 cell lineage commitment | 0.014687619 | 0.028566321 | 0.008052676 |
| BP | GO:0097201 | negative regulation of transcription from RNA polymerase II promoter in response to stress | 0.014687619 | 0.028566321 | 0.008052676 |
| BP | GO:0097284 | hepatocyte apoptotic process | 0.014687619 | 0.028566321 | 0.008052676 |
| BP | GO:0097531 | mast cell migration | 0.014687619 | 0.028566321 | 0.008052676 |
| BP | GO:0150065 | regulation of deacetylase activity | 0.014687619 | 0.028566321 | 0.008052676 |
| BP | GO:1900402 | regulation of carbohydrate metabolic process by regulation of transcription from RNA polymerase II promoter | 0.014687619 | 0.028566321 | 0.008052676 |
| BP | GO:1902510 | regulation of apoptotic DNA fragmentation | 0.014687619 | 0.028566321 | 0.008052676 |
| BP | GO:1905461 | positive regulation of vascular associated smooth muscle cell apoptotic process | 0.014687619 | 0.028566321 | 0.008052676 |
| BP | GO:2000121 | regulation of removal of superoxide radicals | 0.014687619 | 0.028566321 | 0.008052676 |
| BP | GO:2000341 | regulation of chemokine (C-X-C motif) ligand 2 production | 0.014687619 | 0.028566321 | 0.008052676 |
| BP | GO:0016202 | regulation of striated muscle tissue development | 0.014893724 | 0.028944408 | 0.008159256 |
| BP | GO:1990845 | adaptive thermogenesis | 0.015266826 | 0.029646186 | 0.008357084 |
| BP | GO:1901861 | regulation of muscle tissue development | 0.015454892 | 0.029964308 | 0.00844676 |
| BP | GO:1903707 | negative regulation of hemopoiesis | 0.015454892 | 0.029964308 | 0.00844676 |
| BP | GO:0042129 | regulation of T cell proliferation | 0.015643964 | 0.030238699 | 0.008524109 |
| BP | GO:0048634 | regulation of muscle organ development | 0.015643964 | 0.030238699 | 0.008524109 |
| BP | GO:0051053 | negative regulation of DNA metabolic process | 0.015643964 | 0.030238699 | 0.008524109 |
| BP | GO:0043271 | negative regulation of ion transport | 0.015834041 | 0.030238699 | 0.008524109 |
| BP | GO:0007494 | midgut development | 0.015902228 | 0.030238699 | 0.008524109 |
| BP | GO:0010763 | positive regulation of fibroblast migration | 0.015902228 | 0.030238699 | 0.008524109 |
| BP | GO:0014745 | negative regulation of muscle adaptation | 0.015902228 | 0.030238699 | 0.008524109 |
| BP | GO:0031953 | negative regulation of protein autophosphorylation | 0.015902228 | 0.030238699 | 0.008524109 |
| BP | GO:0032725 | positive regulation of granulocyte macrophage colony-stimulating factor production | 0.015902228 | 0.030238699 | 0.008524109 |
| BP | GO:0033127 | regulation of histone phosphorylation | 0.015902228 | 0.030238699 | 0.008524109 |
| BP | GO:0035745 | T-helper 2 cell cytokine production | 0.015902228 | 0.030238699 | 0.008524109 |
| BP | GO:0042635 | positive regulation of hair cycle | 0.015902228 | 0.030238699 | 0.008524109 |
| BP | GO:0045086 | positive regulation of interleukin-2 biosynthetic process | 0.015902228 | 0.030238699 | 0.008524109 |
| BP | GO:0045602 | negative regulation of endothelial cell differentiation | 0.015902228 | 0.030238699 | 0.008524109 |
| BP | GO:0045779 | negative regulation of bone resorption | 0.015902228 | 0.030238699 | 0.008524109 |
| BP | GO:0048875 | chemical homeostasis within a tissue | 0.015902228 | 0.030238699 | 0.008524109 |
| BP | GO:0050872 | white fat cell differentiation | 0.015902228 | 0.030238699 | 0.008524109 |
| BP | GO:0060100 | positive regulation of phagocytosis, engulfment | 0.015902228 | 0.030238699 | 0.008524109 |
| BP | GO:0060576 | intestinal epithelial cell development | 0.015902228 | 0.030238699 | 0.008524109 |
| BP | GO:0060712 | spongiotrophoblast layer development | 0.015902228 | 0.030238699 | 0.008524109 |
| BP | GO:0072182 | regulation of nephron tubule epithelial cell differentiation | 0.015902228 | 0.030238699 | 0.008524109 |
| BP | GO:1902894 | negative regulation of pri-miRNA transcription by RNA polymerase II | 0.015902228 | 0.030238699 | 0.008524109 |
| BP | GO:1902947 | regulation of tau-protein kinase activity | 0.015902228 | 0.030238699 | 0.008524109 |
| BP | GO:1903960 | negative regulation of anion transmembrane transport | 0.015902228 | 0.030238699 | 0.008524109 |
| BP | GO:1905155 | positive regulation of membrane invagination | 0.015902228 | 0.030238699 | 0.008524109 |
| BP | GO:0043467 | regulation of generation of precursor metabolites and energy | 0.016025118 | 0.030448957 | 0.00858338 |
| BP | GO:0030203 | glycosaminoglycan metabolic process | 0.016410268 | 0.031132911 | 0.008776183 |
| BP | GO:0046661 | male sex differentiation | 0.016410268 | 0.031132911 | 0.008776183 |
| BP | GO:0035967 | cellular response to topologically incorrect protein | 0.016604335 | 0.03147693 | 0.008873159 |
| BP | GO:0044282 | small molecule catabolic process | 0.016716537 | 0.031665348 | 0.008926273 |
| BP | GO:0000723 | telomere maintenance | 0.016799393 | 0.031797933 | 0.008963648 |
| BP | GO:0030213 | hyaluronan biosynthetic process | 0.017115406 | 0.032003996 | 0.009021736 |
| BP | GO:0032310 | prostaglandin secretion | 0.017115406 | 0.032003996 | 0.009021736 |
| BP | GO:0036295 | cellular response to increased oxygen levels | 0.017115406 | 0.032003996 | 0.009021736 |
| BP | GO:0048308 | organelle inheritance | 0.017115406 | 0.032003996 | 0.009021736 |
| BP | GO:0048313 | Golgi inheritance | 0.017115406 | 0.032003996 | 0.009021736 |
| BP | GO:0050862 | positive regulation of T cell receptor signaling pathway | 0.017115406 | 0.032003996 | 0.009021736 |
| BP | GO:0070234 | positive regulation of T cell apoptotic process | 0.017115406 | 0.032003996 | 0.009021736 |
| BP | GO:0071391 | cellular response to estrogen stimulus | 0.017115406 | 0.032003996 | 0.009021736 |
| BP | GO:0072216 | positive regulation of metanephros development | 0.017115406 | 0.032003996 | 0.009021736 |
| BP | GO:0072567 | chemokine (C-X-C motif) ligand 2 production | 0.017115406 | 0.032003996 | 0.009021736 |
| BP | GO:0097202 | activation of cysteine-type endopeptidase activity | 0.017115406 | 0.032003996 | 0.009021736 |
| BP | GO:1901722 | regulation of cell proliferation involved in kidney development | 0.017115406 | 0.032003996 | 0.009021736 |
| BP | GO:1903351 | cellular response to dopamine | 0.017115406 | 0.032003996 | 0.009021736 |
| BP | GO:1903624 | regulation of DNA catabolic process | 0.017115406 | 0.032003996 | 0.009021736 |
| BP | GO:1905050 | positive regulation of metallopeptidase activity | 0.017115406 | 0.032003996 | 0.009021736 |
| BP | GO:2001279 | regulation of unsaturated fatty acid biosynthetic process | 0.017115406 | 0.032003996 | 0.009021736 |
| BP | GO:0007088 | regulation of mitotic nuclear division | 0.017192474 | 0.032099544 | 0.009048671 |
| BP | GO:0035821 | modification of morphology or physiology of other organism | 0.017192474 | 0.032099544 | 0.009048671 |
| BP | GO:0071695 | anatomical structure maturation | 0.017390491 | 0.032420283 | 0.009139085 |
| BP | GO:2001242 | regulation of intrinsic apoptotic signaling pathway | 0.017390491 | 0.032420283 | 0.009139085 |
| BP | GO:0016570 | histone modification | 0.017626689 | 0.032835851 | 0.009256231 |
| BP | GO:0050728 | negative regulation of inflammatory response | 0.018192349 | 0.033583929 | 0.00946711 |
| BP | GO:0010759 | positive regulation of macrophage chemotaxis | 0.018327152 | 0.033583929 | 0.00946711 |
| BP | GO:0015671 | oxygen transport | 0.018327152 | 0.033583929 | 0.00946711 |
| BP | GO:0030812 | negative regulation of nucleotide catabolic process | 0.018327152 | 0.033583929 | 0.00946711 |
| BP | GO:0032645 | regulation of granulocyte macrophage colony-stimulating factor production | 0.018327152 | 0.033583929 | 0.00946711 |
| BP | GO:0035635 | entry of bacterium into host cell | 0.018327152 | 0.033583929 | 0.00946711 |
| BP | GO:0045838 | positive regulation of membrane potential | 0.018327152 | 0.033583929 | 0.00946711 |
| BP | GO:0046321 | positive regulation of fatty acid oxidation | 0.018327152 | 0.033583929 | 0.00946711 |
| BP | GO:0046851 | negative regulation of bone remodeling | 0.018327152 | 0.033583929 | 0.00946711 |
| BP | GO:0048569 | post-embryonic animal organ development | 0.018327152 | 0.033583929 | 0.00946711 |
| BP | GO:0051198 | negative regulation of coenzyme metabolic process | 0.018327152 | 0.033583929 | 0.00946711 |
| BP | GO:0051770 | positive regulation of nitric-oxide synthase biosynthetic process | 0.018327152 | 0.033583929 | 0.00946711 |
| BP | GO:0060099 | regulation of phagocytosis, engulfment | 0.018327152 | 0.033583929 | 0.00946711 |
| BP | GO:0060841 | venous blood vessel development | 0.018327152 | 0.033583929 | 0.00946711 |
| BP | GO:0071801 | regulation of podosome assembly | 0.018327152 | 0.033583929 | 0.00946711 |
| BP | GO:0072160 | nephron tubule epithelial cell differentiation | 0.018327152 | 0.033583929 | 0.00946711 |
| BP | GO:0072283 | metanephric renal vesicle morphogenesis | 0.018327152 | 0.033583929 | 0.00946711 |
| BP | GO:0090197 | positive regulation of chemokine secretion | 0.018327152 | 0.033583929 | 0.00946711 |
| BP | GO:1903350 | response to dopamine | 0.018327152 | 0.033583929 | 0.00946711 |
| BP | GO:1903358 | regulation of Golgi organization | 0.018327152 | 0.033583929 | 0.00946711 |
| BP | GO:2000402 | negative regulation of lymphocyte migration | 0.018327152 | 0.033583929 | 0.00946711 |
| BP | GO:2001028 | positive regulation of endothelial cell chemotaxis | 0.018327152 | 0.033583929 | 0.00946711 |
| BP | GO:0006022 | aminoglycan metabolic process | 0.018395248 | 0.033683743 | 0.009495247 |
| BP | GO:0003205 | cardiac chamber development | 0.018599116 | 0.034006668 | 0.009586278 |
| BP | GO:0016241 | regulation of macroautophagy | 0.018599116 | 0.034006668 | 0.009586278 |
| BP | GO:0008544 | epidermis development | 0.018670315 | 0.034111617 | 0.009615862 |
| BP | GO:0016525 | negative regulation of angiogenesis | 0.019009749 | 0.03470613 | 0.009783452 |
| BP | GO:0032200 | telomere organization | 0.019424228 | 0.035022934 | 0.009872757 |
| BP | GO:0046165 | alcohol biosynthetic process | 0.019424228 | 0.035022934 | 0.009872757 |
| BP | GO:2000181 | negative regulation of blood vessel morphogenesis | 0.019424228 | 0.035022934 | 0.009872757 |
| BP | GO:0001780 | neutrophil homeostasis | 0.01953747 | 0.035022934 | 0.009872757 |
| BP | GO:0002070 | epithelial cell maturation | 0.01953747 | 0.035022934 | 0.009872757 |
| BP | GO:0002295 | T-helper cell lineage commitment | 0.01953747 | 0.035022934 | 0.009872757 |
| BP | GO:0002830 | positive regulation of type 2 immune response | 0.01953747 | 0.035022934 | 0.009872757 |
| BP | GO:0006206 | pyrimidine nucleobase metabolic process | 0.01953747 | 0.035022934 | 0.009872757 |
| BP | GO:0010225 | response to UV-C | 0.01953747 | 0.035022934 | 0.009872757 |
| BP | GO:0010875 | positive regulation of cholesterol efflux | 0.01953747 | 0.035022934 | 0.009872757 |
| BP | GO:0010885 | regulation of cholesterol storage | 0.01953747 | 0.035022934 | 0.009872757 |
| BP | GO:0032305 | positive regulation of icosanoid secretion | 0.01953747 | 0.035022934 | 0.009872757 |
| BP | GO:0032604 | granulocyte macrophage colony-stimulating factor production | 0.01953747 | 0.035022934 | 0.009872757 |
| BP | GO:0043217 | myelin maintenance | 0.01953747 | 0.035022934 | 0.009872757 |
| BP | GO:0046325 | negative regulation of glucose import | 0.01953747 | 0.035022934 | 0.009872757 |
| BP | GO:0070886 | positive regulation of calcineurin-NFAT signaling cascade | 0.01953747 | 0.035022934 | 0.009872757 |
| BP | GO:0090036 | regulation of protein kinase C signaling | 0.01953747 | 0.035022934 | 0.009872757 |
| BP | GO:0090153 | regulation of sphingolipid biosynthetic process | 0.01953747 | 0.035022934 | 0.009872757 |
| BP | GO:0090335 | regulation of brown fat cell differentiation | 0.01953747 | 0.035022934 | 0.009872757 |
| BP | GO:0106058 | positive regulation of calcineurin-mediated signaling | 0.01953747 | 0.035022934 | 0.009872757 |
| BP | GO:1902001 | fatty acid transmembrane transport | 0.01953747 | 0.035022934 | 0.009872757 |
| BP | GO:1902004 | positive regulation of amyloid-beta formation | 0.01953747 | 0.035022934 | 0.009872757 |
| BP | GO:1905038 | regulation of membrane lipid metabolic process | 0.01953747 | 0.035022934 | 0.009872757 |
| BP | GO:1905153 | regulation of membrane invagination | 0.01953747 | 0.035022934 | 0.009872757 |
| BP | GO:2000303 | regulation of ceramide biosynthetic process | 0.01953747 | 0.035022934 | 0.009872757 |
| BP | GO:0006986 | response to unfolded protein | 0.019632903 | 0.035143039 | 0.009906614 |
| BP | GO:0050796 | regulation of insulin secretion | 0.019632903 | 0.035143039 | 0.009906614 |
| BP | GO:0016569 | covalent chromatin modification | 0.019748099 | 0.035323662 | 0.00995753 |
| BP | GO:0043488 | regulation of mRNA stability | 0.019842533 | 0.035466914 | 0.009997912 |
| BP | GO:0050821 | protein stabilization | 0.020053114 | 0.035817412 | 0.010096716 |
| BP | GO:0006611 | protein export from nucleus | 0.020264645 | 0.03606494 | 0.010166492 |
| BP | GO:0008361 | regulation of cell size | 0.020264645 | 0.03606494 | 0.010166492 |
| BP | GO:0051099 | positive regulation of binding | 0.020264645 | 0.03606494 | 0.010166492 |
| BP | GO:1903034 | regulation of response to wounding | 0.020264645 | 0.03606494 | 0.010166492 |
| BP | GO:2001235 | positive regulation of apoptotic signaling pathway | 0.020264645 | 0.03606494 | 0.010166492 |
| BP | GO:0071466 | cellular response to xenobiotic stimulus | 0.020477122 | 0.036116199 | 0.010180942 |
| BP | GO:0015711 | organic anion transport | 0.020634985 | 0.036116199 | 0.010180942 |
| BP | GO:0002285 | lymphocyte activation involved in immune response | 0.020690544 | 0.036116199 | 0.010180942 |
| BP | GO:0022408 | negative regulation of cell-cell adhesion | 0.020690544 | 0.036116199 | 0.010180942 |
| BP | GO:0003184 | pulmonary valve morphogenesis | 0.02074636 | 0.036116199 | 0.010180942 |
| BP | GO:0006089 | lactate metabolic process | 0.02074636 | 0.036116199 | 0.010180942 |
| BP | GO:0008340 | determination of adult lifespan | 0.02074636 | 0.036116199 | 0.010180942 |
| BP | GO:0010715 | regulation of extracellular matrix disassembly | 0.02074636 | 0.036116199 | 0.010180942 |
| BP | GO:0015732 | prostaglandin transport | 0.02074636 | 0.036116199 | 0.010180942 |
| BP | GO:0030540 | female genitalia development | 0.02074636 | 0.036116199 | 0.010180942 |
| BP | GO:0031000 | response to caffeine | 0.02074636 | 0.036116199 | 0.010180942 |
| BP | GO:0032740 | positive regulation of interleukin-17 production | 0.02074636 | 0.036116199 | 0.010180942 |
| BP | GO:0033599 | regulation of mammary gland epithelial cell proliferation | 0.02074636 | 0.036116199 | 0.010180942 |
| BP | GO:0035729 | cellular response to hepatocyte growth factor stimulus | 0.02074636 | 0.036116199 | 0.010180942 |
| BP | GO:0036270 | response to diuretic | 0.02074636 | 0.036116199 | 0.010180942 |
| BP | GO:0042953 | lipoprotein transport | 0.02074636 | 0.036116199 | 0.010180942 |
| BP | GO:0042994 | cytoplasmic sequestering of transcription factor | 0.02074636 | 0.036116199 | 0.010180942 |
| BP | GO:0043373 | CD4-positive, alpha-beta T cell lineage commitment | 0.02074636 | 0.036116199 | 0.010180942 |
| BP | GO:0043923 | positive regulation by host of viral transcription | 0.02074636 | 0.036116199 | 0.010180942 |
| BP | GO:0044872 | lipoprotein localization | 0.02074636 | 0.036116199 | 0.010180942 |
| BP | GO:0045091 | regulation of single stranded viral RNA replication via double stranded DNA intermediate | 0.02074636 | 0.036116199 | 0.010180942 |
| BP | GO:0045725 | positive regulation of glycogen biosynthetic process | 0.02074636 | 0.036116199 | 0.010180942 |
| BP | GO:0060644 | mammary gland epithelial cell differentiation | 0.02074636 | 0.036116199 | 0.010180942 |
| BP | GO:0060850 | regulation of transcription involved in cell fate commitment | 0.02074636 | 0.036116199 | 0.010180942 |
| BP | GO:0062033 | positive regulation of mitotic sister chromatid segregation | 0.02074636 | 0.036116199 | 0.010180942 |
| BP | GO:0070242 | thymocyte apoptotic process | 0.02074636 | 0.036116199 | 0.010180942 |
| BP | GO:0090185 | negative regulation of kidney development | 0.02074636 | 0.036116199 | 0.010180942 |
| BP | GO:1901524 | regulation of mitophagy | 0.02074636 | 0.036116199 | 0.010180942 |
| BP | GO:1904355 | positive regulation of telomere capping | 0.02074636 | 0.036116199 | 0.010180942 |
| BP | GO:2000641 | regulation of early endosome to late endosome transport | 0.02074636 | 0.036116199 | 0.010180942 |
| BP | GO:2001267 | regulation of cysteine-type endopeptidase activity involved in apoptotic signaling pathway | 0.02074636 | 0.036116199 | 0.010180942 |
| BP | GO:0008217 | regulation of blood pressure | 0.020904907 | 0.036366595 | 0.010251527 |
| BP | GO:0002040 | sprouting angiogenesis | 0.021120211 | 0.036689501 | 0.010342552 |
| BP | GO:0043487 | regulation of RNA stability | 0.021120211 | 0.036689501 | 0.010342552 |
| BP | GO:0050864 | regulation of B cell activation | 0.021336451 | 0.03703912 | 0.010441108 |
| BP | GO:0007221 | positive regulation of transcription of Notch receptor target | 0.021953825 | 0.03753102 | 0.010579771 |
| BP | GO:0007252 | I-kappaB phosphorylation | 0.021953825 | 0.03753102 | 0.010579771 |
| BP | GO:0010878 | cholesterol storage | 0.021953825 | 0.03753102 | 0.010579771 |
| BP | GO:0031293 | membrane protein intracellular domain proteolysis | 0.021953825 | 0.03753102 | 0.010579771 |
| BP | GO:0031998 | regulation of fatty acid beta-oxidation | 0.021953825 | 0.03753102 | 0.010579771 |
| BP | GO:0039692 | single stranded viral RNA replication via double stranded DNA intermediate | 0.021953825 | 0.03753102 | 0.010579771 |
| BP | GO:0045780 | positive regulation of bone resorption | 0.021953825 | 0.03753102 | 0.010579771 |
| BP | GO:0046852 | positive regulation of bone remodeling | 0.021953825 | 0.03753102 | 0.010579771 |
| BP | GO:0048739 | cardiac muscle fiber development | 0.021953825 | 0.03753102 | 0.010579771 |
| BP | GO:0051782 | negative regulation of cell division | 0.021953825 | 0.03753102 | 0.010579771 |
| BP | GO:0060977 | coronary vasculature morphogenesis | 0.021953825 | 0.03753102 | 0.010579771 |
| BP | GO:0070230 | positive regulation of lymphocyte apoptotic process | 0.021953825 | 0.03753102 | 0.010579771 |
| BP | GO:0070875 | positive regulation of glycogen metabolic process | 0.021953825 | 0.03753102 | 0.010579771 |
| BP | GO:0071605 | monocyte chemotactic protein-1 production | 0.021953825 | 0.03753102 | 0.010579771 |
| BP | GO:0071637 | regulation of monocyte chemotactic protein-1 production | 0.021953825 | 0.03753102 | 0.010579771 |
| BP | GO:0072077 | renal vesicle morphogenesis | 0.021953825 | 0.03753102 | 0.010579771 |
| BP | GO:0090190 | positive regulation of branching involved in ureteric bud morphogenesis | 0.021953825 | 0.03753102 | 0.010579771 |
| BP | GO:0090196 | regulation of chemokine secretion | 0.021953825 | 0.03753102 | 0.010579771 |
| BP | GO:1900221 | regulation of amyloid-beta clearance | 0.021953825 | 0.03753102 | 0.010579771 |
| BP | GO:2000047 | regulation of cell-cell adhesion mediated by cadherin | 0.021953825 | 0.03753102 | 0.010579771 |
| BP | GO:2000193 | positive regulation of fatty acid transport | 0.021953825 | 0.03753102 | 0.010579771 |
| BP | GO:2000647 | negative regulation of stem cell proliferation | 0.021953825 | 0.03753102 | 0.010579771 |
| BP | GO:1901343 | negative regulation of vasculature development | 0.021990769 | 0.037568198 | 0.010590252 |
| BP | GO:0051783 | regulation of nuclear division | 0.022210734 | 0.037917772 | 0.010688795 |
| BP | GO:0042119 | neutrophil activation | 0.02247468 | 0.038341897 | 0.010808353 |
| BP | GO:0002446 | neutrophil mediated immunity | 0.022592584 | 0.03851646 | 0.010857561 |
| BP | GO:0002363 | alpha-beta T cell lineage commitment | 0.023159865 | 0.038788066 | 0.010934125 |
| BP | GO:0002544 | chronic inflammatory response | 0.023159865 | 0.038788066 | 0.010934125 |
| BP | GO:0010544 | negative regulation of platelet activation | 0.023159865 | 0.038788066 | 0.010934125 |
| BP | GO:0010663 | positive regulation of striated muscle cell apoptotic process | 0.023159865 | 0.038788066 | 0.010934125 |
| BP | GO:0010666 | positive regulation of cardiac muscle cell apoptotic process | 0.023159865 | 0.038788066 | 0.010934125 |
| BP | GO:0015669 | gas transport | 0.023159865 | 0.038788066 | 0.010934125 |
| BP | GO:0032026 | response to magnesium ion | 0.023159865 | 0.038788066 | 0.010934125 |
| BP | GO:0032303 | regulation of icosanoid secretion | 0.023159865 | 0.038788066 | 0.010934125 |
| BP | GO:0032930 | positive regulation of superoxide anion generation | 0.023159865 | 0.038788066 | 0.010934125 |
| BP | GO:0033189 | response to vitamin A | 0.023159865 | 0.038788066 | 0.010934125 |
| BP | GO:0034138 | toll-like receptor 3 signaling pathway | 0.023159865 | 0.038788066 | 0.010934125 |
| BP | GO:0035728 | response to hepatocyte growth factor | 0.023159865 | 0.038788066 | 0.010934125 |
| BP | GO:0036303 | lymph vessel morphogenesis | 0.023159865 | 0.038788066 | 0.010934125 |
| BP | GO:0045076 | regulation of interleukin-2 biosynthetic process | 0.023159865 | 0.038788066 | 0.010934125 |
| BP | GO:0045414 | regulation of interleukin-8 biosynthetic process | 0.023159865 | 0.038788066 | 0.010934125 |
| BP | GO:0060231 | mesenchymal to epithelial transition | 0.023159865 | 0.038788066 | 0.010934125 |
| BP | GO:0060438 | trachea development | 0.023159865 | 0.038788066 | 0.010934125 |
| BP | GO:0071800 | podosome assembly | 0.023159865 | 0.038788066 | 0.010934125 |
| BP | GO:0072087 | renal vesicle development | 0.023159865 | 0.038788066 | 0.010934125 |
| BP | GO:0090201 | negative regulation of release of cytochrome c from mitochondria | 0.023159865 | 0.038788066 | 0.010934125 |
| BP | GO:1900409 | positive regulation of cellular response to oxidative stress | 0.023159865 | 0.038788066 | 0.010934125 |
| BP | GO:1901741 | positive regulation of myoblast fusion | 0.023159865 | 0.038788066 | 0.010934125 |
| BP | GO:1902176 | negative regulation of oxidative stress-induced intrinsic apoptotic signaling pathway | 0.023159865 | 0.038788066 | 0.010934125 |
| BP | GO:1905288 | vascular associated smooth muscle cell apoptotic process | 0.023159865 | 0.038788066 | 0.010934125 |
| BP | GO:1905459 | regulation of vascular associated smooth muscle cell apoptotic process | 0.023159865 | 0.038788066 | 0.010934125 |
| BP | GO:2000696 | regulation of epithelial cell differentiation involved in kidney development | 0.023159865 | 0.038788066 | 0.010934125 |
| BP | GO:0002377 | immunoglobulin production | 0.023324383 | 0.03901074 | 0.010996896 |
| BP | GO:0051224 | negative regulation of protein transport | 0.023324383 | 0.03901074 | 0.010996896 |
| BP | GO:0051168 | nuclear export | 0.023549861 | 0.039361228 | 0.011095696 |
| BP | GO:0006694 | steroid biosynthetic process | 0.024003547 | 0.040092412 | 0.011301813 |
| BP | GO:1904950 | negative regulation of establishment of protein localization | 0.024231751 | 0.040125916 | 0.011311257 |
| BP | GO:0001502 | cartilage condensation | 0.024364482 | 0.040125916 | 0.011311257 |
| BP | GO:0002827 | positive regulation of T-helper 1 type immune response | 0.024364482 | 0.040125916 | 0.011311257 |
| BP | GO:0006525 | arginine metabolic process | 0.024364482 | 0.040125916 | 0.011311257 |
| BP | GO:0032042 | mitochondrial DNA metabolic process | 0.024364482 | 0.040125916 | 0.011311257 |
| BP | GO:0032793 | positive regulation of CREB transcription factor activity | 0.024364482 | 0.040125916 | 0.011311257 |
| BP | GO:0034104 | negative regulation of tissue remodeling | 0.024364482 | 0.040125916 | 0.011311257 |
| BP | GO:0034393 | positive regulation of smooth muscle cell apoptotic process | 0.024364482 | 0.040125916 | 0.011311257 |
| BP | GO:0035458 | cellular response to interferon-beta | 0.024364482 | 0.040125916 | 0.011311257 |
| BP | GO:0042228 | interleukin-8 biosynthetic process | 0.024364482 | 0.040125916 | 0.011311257 |
| BP | GO:0043369 | CD4-positive or CD8-positive, alpha-beta T cell lineage commitment | 0.024364482 | 0.040125916 | 0.011311257 |
| BP | GO:0043586 | tongue development | 0.024364482 | 0.040125916 | 0.011311257 |
| BP | GO:0051767 | nitric-oxide synthase biosynthetic process | 0.024364482 | 0.040125916 | 0.011311257 |
| BP | GO:0051769 | regulation of nitric-oxide synthase biosynthetic process | 0.024364482 | 0.040125916 | 0.011311257 |
| BP | GO:0051797 | regulation of hair follicle development | 0.024364482 | 0.040125916 | 0.011311257 |
| BP | GO:0060602 | branch elongation of an epithelium | 0.024364482 | 0.040125916 | 0.011311257 |
| BP | GO:0072234 | metanephric nephron tubule development | 0.024364482 | 0.040125916 | 0.011311257 |
| BP | GO:0090195 | chemokine secretion | 0.024364482 | 0.040125916 | 0.011311257 |
| BP | GO:1902993 | positive regulation of amyloid precursor protein catabolic process | 0.024364482 | 0.040125916 | 0.011311257 |
| BP | GO:1903978 | regulation of microglial cell activation | 0.024364482 | 0.040125916 | 0.011311257 |
| BP | GO:1905523 | positive regulation of macrophage migration | 0.024364482 | 0.040125916 | 0.011311257 |
| BP | GO:0035966 | response to topologically incorrect protein | 0.024690866 | 0.040609328 | 0.011447528 |
| BP | GO:0061013 | regulation of mRNA catabolic process | 0.024690866 | 0.040609328 | 0.011447528 |
| BP | GO:0050852 | T cell receptor signaling pathway | 0.025386274 | 0.041499214 | 0.011698382 |
| BP | GO:0003177 | pulmonary valve development | 0.025567679 | 0.041499214 | 0.011698382 |
| BP | GO:0030728 | ovulation | 0.025567679 | 0.041499214 | 0.011698382 |
| BP | GO:0031290 | retinal ganglion cell axon guidance | 0.025567679 | 0.041499214 | 0.011698382 |
| BP | GO:0032332 | positive regulation of chondrocyte differentiation | 0.025567679 | 0.041499214 | 0.011698382 |
| BP | GO:0032682 | negative regulation of chemokine production | 0.025567679 | 0.041499214 | 0.011698382 |
| BP | GO:0032891 | negative regulation of organic acid transport | 0.025567679 | 0.041499214 | 0.011698382 |
| BP | GO:0032986 | protein-DNA complex disassembly | 0.025567679 | 0.041499214 | 0.011698382 |
| BP | GO:0035162 | embryonic hemopoiesis | 0.025567679 | 0.041499214 | 0.011698382 |
| BP | GO:0035357 | peroxisome proliferator activated receptor signaling pathway | 0.025567679 | 0.041499214 | 0.011698382 |
| BP | GO:0036499 | PERK-mediated unfolded protein response | 0.025567679 | 0.041499214 | 0.011698382 |
| BP | GO:0043153 | entrainment of circadian clock by photoperiod | 0.025567679 | 0.041499214 | 0.011698382 |
| BP | GO:0061213 | positive regulation of mesonephros development | 0.025567679 | 0.041499214 | 0.011698382 |
| BP | GO:0071459 | protein localization to chromosome, centromeric region | 0.025567679 | 0.041499214 | 0.011698382 |
| BP | GO:0098743 | cell aggregation | 0.025567679 | 0.041499214 | 0.011698382 |
| BP | GO:1901739 | regulation of myoblast fusion | 0.025567679 | 0.041499214 | 0.011698382 |
| BP | GO:1902884 | positive regulation of response to oxidative stress | 0.025567679 | 0.041499214 | 0.011698382 |
| BP | GO:1903392 | negative regulation of adherens junction organization | 0.025567679 | 0.041499214 | 0.011698382 |
| BP | GO:2000269 | regulation of fibroblast apoptotic process | 0.025567679 | 0.041499214 | 0.011698382 |
| BP | GO:2000810 | regulation of bicellular tight junction assembly | 0.025567679 | 0.041499214 | 0.011698382 |
| BP | GO:0034764 | positive regulation of transmembrane transport | 0.025854342 | 0.041936964 | 0.011821781 |
| BP | GO:0006109 | regulation of carbohydrate metabolic process | 0.026325958 | 0.042673947 | 0.012029532 |
| BP | GO:0030073 | insulin secretion | 0.026563091 | 0.042775757 | 0.012058232 |
| BP | GO:0000423 | mitophagy | 0.026769456 | 0.042775757 | 0.012058232 |
| BP | GO:0002052 | positive regulation of neuroblast proliferation | 0.026769456 | 0.042775757 | 0.012058232 |
| BP | GO:0006925 | inflammatory cell apoptotic process | 0.026769456 | 0.042775757 | 0.012058232 |
| BP | GO:0007063 | regulation of sister chromatid cohesion | 0.026769456 | 0.042775757 | 0.012058232 |
| BP | GO:0010869 | regulation of receptor biosynthetic process | 0.026769456 | 0.042775757 | 0.012058232 |
| BP | GO:0030878 | thyroid gland development | 0.026769456 | 0.042775757 | 0.012058232 |
| BP | GO:0032069 | regulation of nuclease activity | 0.026769456 | 0.042775757 | 0.012058232 |
| BP | GO:0032727 | positive regulation of interferon-alpha production | 0.026769456 | 0.042775757 | 0.012058232 |
| BP | GO:0032928 | regulation of superoxide anion generation | 0.026769456 | 0.042775757 | 0.012058232 |
| BP | GO:0033233 | regulation of protein sumoylation | 0.026769456 | 0.042775757 | 0.012058232 |
| BP | GO:0042094 | interleukin-2 biosynthetic process | 0.026769456 | 0.042775757 | 0.012058232 |
| BP | GO:0044321 | response to leptin | 0.026769456 | 0.042775757 | 0.012058232 |
| BP | GO:0045663 | positive regulation of myoblast differentiation | 0.026769456 | 0.042775757 | 0.012058232 |
| BP | GO:0045723 | positive regulation of fatty acid biosynthetic process | 0.026769456 | 0.042775757 | 0.012058232 |
| BP | GO:0051195 | negative regulation of cofactor metabolic process | 0.026769456 | 0.042775757 | 0.012058232 |
| BP | GO:0060065 | uterus development | 0.026769456 | 0.042775757 | 0.012058232 |
| BP | GO:0060575 | intestinal epithelial cell differentiation | 0.026769456 | 0.042775757 | 0.012058232 |
| BP | GO:0070935 | 3'-UTR-mediated mRNA stabilization | 0.026769456 | 0.042775757 | 0.012058232 |
| BP | GO:0071404 | cellular response to low-density lipoprotein particle stimulus | 0.026769456 | 0.042775757 | 0.012058232 |
| BP | GO:0090189 | regulation of branching involved in ureteric bud morphogenesis | 0.026769456 | 0.042775757 | 0.012058232 |
| BP | GO:1903589 | positive regulation of blood vessel endothelial cell proliferation involved in sprouting angiogenesis | 0.026769456 | 0.042775757 | 0.012058232 |
| BP | GO:1901215 | negative regulation of neuron death | 0.026801105 | 0.042798663 | 0.012064689 |
| BP | GO:0051216 | cartilage development | 0.027039995 | 0.043152272 | 0.012164369 |
| BP | GO:1903531 | negative regulation of secretion by cell | 0.027520401 | 0.043890602 | 0.0123725 |
| BP | GO:0007064 | mitotic sister chromatid cohesion | 0.027969816 | 0.044264651 | 0.012477942 |
| BP | GO:0032373 | positive regulation of sterol transport | 0.027969816 | 0.044264651 | 0.012477942 |
| BP | GO:0032376 | positive regulation of cholesterol transport | 0.027969816 | 0.044264651 | 0.012477942 |
| BP | GO:0032799 | low-density lipoprotein receptor particle metabolic process | 0.027969816 | 0.044264651 | 0.012477942 |
| BP | GO:0048714 | positive regulation of oligodendrocyte differentiation | 0.027969816 | 0.044264651 | 0.012477942 |
| BP | GO:0051349 | positive regulation of lyase activity | 0.027969816 | 0.044264651 | 0.012477942 |
| BP | GO:0060445 | branching involved in salivary gland morphogenesis | 0.027969816 | 0.044264651 | 0.012477942 |
| BP | GO:0071157 | negative regulation of cell cycle arrest | 0.027969816 | 0.044264651 | 0.012477942 |
| BP | GO:0072170 | metanephric tubule development | 0.027969816 | 0.044264651 | 0.012477942 |
| BP | GO:0072243 | metanephric nephron epithelium development | 0.027969816 | 0.044264651 | 0.012477942 |
| BP | GO:0090343 | positive regulation of cell aging | 0.027969816 | 0.044264651 | 0.012477942 |
| BP | GO:1901889 | negative regulation of cell junction assembly | 0.027969816 | 0.044264651 | 0.012477942 |
| BP | GO:1902749 | regulation of cell cycle G2/M phase transition | 0.028004291 | 0.044290855 | 0.012485329 |
| BP | GO:0002092 | positive regulation of receptor internalization | 0.029168759 | 0.045781062 | 0.012905409 |
| BP | GO:0002861 | regulation of inflammatory response to antigenic stimulus | 0.029168759 | 0.045781062 | 0.012905409 |
| BP | GO:0032897 | negative regulation of viral transcription | 0.029168759 | 0.045781062 | 0.012905409 |
| BP | GO:0046697 | decidualization | 0.029168759 | 0.045781062 | 0.012905409 |
| BP | GO:0050857 | positive regulation of antigen receptor-mediated signaling pathway | 0.029168759 | 0.045781062 | 0.012905409 |
| BP | GO:0050996 | positive regulation of lipid catabolic process | 0.029168759 | 0.045781062 | 0.012905409 |
| BP | GO:0051220 | cytoplasmic sequestering of protein | 0.029168759 | 0.045781062 | 0.012905409 |
| BP | GO:0071677 | positive regulation of mononuclear cell migration | 0.029168759 | 0.045781062 | 0.012905409 |
| BP | GO:0090023 | positive regulation of neutrophil chemotaxis | 0.029168759 | 0.045781062 | 0.012905409 |
| BP | GO:1903649 | regulation of cytoplasmic transport | 0.029168759 | 0.045781062 | 0.012905409 |
| BP | GO:2000178 | negative regulation of neural precursor cell proliferation | 0.029168759 | 0.045781062 | 0.012905409 |
| BP | GO:2001026 | regulation of endothelial cell chemotaxis | 0.029168759 | 0.045781062 | 0.012905409 |
| BP | GO:0006814 | sodium ion transport | 0.029229133 | 0.045817639 | 0.01291572 |
| BP | GO:0050807 | regulation of synapse organization | 0.029229133 | 0.045817639 | 0.01291572 |
| BP | GO:0001945 | lymph vessel development | 0.030366288 | 0.047122073 | 0.013283432 |
| BP | GO:0002719 | negative regulation of cytokine production involved in immune response | 0.030366288 | 0.047122073 | 0.013283432 |
| BP | GO:0009299 | mRNA transcription | 0.030366288 | 0.047122073 | 0.013283432 |
| BP | GO:0009648 | photoperiodism | 0.030366288 | 0.047122073 | 0.013283432 |
| BP | GO:0019054 | modulation by virus of host process | 0.030366288 | 0.047122073 | 0.013283432 |
| BP | GO:0032461 | positive regulation of protein oligomerization | 0.030366288 | 0.047122073 | 0.013283432 |
| BP | GO:0045662 | negative regulation of myoblast differentiation | 0.030366288 | 0.047122073 | 0.013283432 |
| BP | GO:0050901 | leukocyte tethering or rolling | 0.030366288 | 0.047122073 | 0.013283432 |
| BP | GO:0051894 | positive regulation of focal adhesion assembly | 0.030366288 | 0.047122073 | 0.013283432 |
| BP | GO:0060444 | branching involved in mammary gland duct morphogenesis | 0.030366288 | 0.047122073 | 0.013283432 |
| BP | GO:0060571 | morphogenesis of an epithelial fold | 0.030366288 | 0.047122073 | 0.013283432 |
| BP | GO:0061217 | regulation of mesonephros development | 0.030366288 | 0.047122073 | 0.013283432 |
| BP | GO:0072202 | cell differentiation involved in metanephros development | 0.030366288 | 0.047122073 | 0.013283432 |
| BP | GO:0072273 | metanephric nephron morphogenesis | 0.030366288 | 0.047122073 | 0.013283432 |
| BP | GO:1904385 | cellular response to angiotensin | 0.030366288 | 0.047122073 | 0.013283432 |
| BP | GO:2000679 | positive regulation of transcription regulatory region DNA binding | 0.030366288 | 0.047122073 | 0.013283432 |
| BP | GO:0050803 | regulation of synapse structure or activity | 0.031487508 | 0.04849115 | 0.013669367 |
| BP | GO:0002230 | positive regulation of defense response to virus by host | 0.031562403 | 0.04849115 | 0.013669367 |
| BP | GO:0006309 | apoptotic DNA fragmentation | 0.031562403 | 0.04849115 | 0.013669367 |
| BP | GO:0010758 | regulation of macrophage chemotaxis | 0.031562403 | 0.04849115 | 0.013669367 |
| BP | GO:0022011 | myelination in peripheral nervous system | 0.031562403 | 0.04849115 | 0.013669367 |
| BP | GO:0031954 | positive regulation of protein autophosphorylation | 0.031562403 | 0.04849115 | 0.013669367 |
| BP | GO:0032292 | peripheral nervous system axon ensheathment | 0.031562403 | 0.04849115 | 0.013669367 |
| BP | GO:0060544 | regulation of necroptotic process | 0.031562403 | 0.04849115 | 0.013669367 |
| BP | GO:0060740 | prostate gland epithelium morphogenesis | 0.031562403 | 0.04849115 | 0.013669367 |
| BP | GO:0071624 | positive regulation of granulocyte chemotaxis | 0.031562403 | 0.04849115 | 0.013669367 |
| BP | GO:0072539 | T-helper 17 cell differentiation | 0.031562403 | 0.04849115 | 0.013669367 |
| BP | GO:0099560 | synaptic membrane adhesion | 0.031562403 | 0.04849115 | 0.013669367 |
| BP | GO:1903579 | negative regulation of ATP metabolic process | 0.031562403 | 0.04849115 | 0.013669367 |
| BP | GO:1903959 | regulation of anion transmembrane transport | 0.031562403 | 0.04849115 | 0.013669367 |
| BP | GO:1904353 | regulation of telomere capping | 0.031562403 | 0.04849115 | 0.013669367 |
| BP | GO:1905048 | regulation of metallopeptidase activity | 0.031562403 | 0.04849115 | 0.013669367 |
| BP | GO:1903320 | regulation of protein modification by small protein conjugation or removal | 0.032513022 | 0.049920615 | 0.014072324 |
| BP | GO:0002026 | regulation of the force of heart contraction | 0.032757107 | 0.049954083 | 0.014081759 |
| BP | GO:0002825 | regulation of T-helper 1 type immune response | 0.032757107 | 0.049954083 | 0.014081759 |
| BP | GO:0009649 | entrainment of circadian clock | 0.032757107 | 0.049954083 | 0.014081759 |
| BP | GO:0010971 | positive regulation of G2/M transition of mitotic cell cycle | 0.032757107 | 0.049954083 | 0.014081759 |
| BP | GO:0043921 | modulation by host of viral transcription | 0.032757107 | 0.049954083 | 0.014081759 |
| BP | GO:0052472 | modulation by host of symbiont transcription | 0.032757107 | 0.049954083 | 0.014081759 |
| BP | GO:0060143 | positive regulation of syncytium formation by plasma membrane fusion | 0.032757107 | 0.049954083 | 0.014081759 |
| BP | GO:0072207 | metanephric epithelium development | 0.032757107 | 0.049954083 | 0.014081759 |
| BP | GO:1902175 | regulation of oxidative stress-induced intrinsic apoptotic signaling pathway | 0.032757107 | 0.049954083 | 0.014081759 |
| BP | GO:1905208 | negative regulation of cardiocyte differentiation | 0.032757107 | 0.049954083 | 0.014081759 |
| BP | GO:1905563 | negative regulation of vascular endothelial cell proliferation | 0.032757107 | 0.049954083 | 0.014081759 |
| CC | GO:0000790 | nuclear chromatin | 1.66E-07 | 1.65E-05 | 1.10E-05 |
| CC | GO:0090575 | RNA polymerase II transcription factor complex | 1.08E-06 | 5.36E-05 | 3.59E-05 |
| CC | GO:0005667 | transcription factor complex | 2.99E-06 | 7.51E-05 | 5.03E-05 |
| CC | GO:0044798 | nuclear transcription factor complex | 3.04E-06 | 7.51E-05 | 5.03E-05 |
| CC | GO:0000307 | cyclin-dependent protein kinase holoenzyme complex | 1.55E-05 | 0.000305936 | 0.000204933 |
| CC | GO:0045121 | membrane raft | 2.68E-05 | 0.000384587 | 0.000257618 |
| CC | GO:0098857 | membrane microdomain | 2.72E-05 | 0.000384587 | 0.000257618 |
| CC | GO:0098589 | membrane region | 3.25E-05 | 0.000402208 | 0.000269421 |
| CC | GO:1902554 | serine/threonine protein kinase complex | 0.000142619 | 0.001568806 | 0.001050875 |
| CC | GO:0061695 | transferase complex, transferring phosphorus-containing groups | 0.000211604 | 0.002094879 | 0.001403268 |
| CC | GO:1902911 | protein kinase complex | 0.000268519 | 0.002416672 | 0.001618824 |
| CC | GO:0031983 | vesicle lumen | 0.000587013 | 0.004842854 | 0.003244017 |
| CC | GO:0005819 | spindle | 0.000640704 | 0.004879205 | 0.003268367 |
| CC | GO:0005901 | caveola | 0.003891901 | 0.027521303 | 0.018435322 |
| CC | GO:0017053 | transcriptional repressor complex | 0.004281275 | 0.028256418 | 0.018927744 |
| CC | GO:0016605 | PML body | 0.005894823 | 0.034631385 | 0.023198057 |
| CC | GO:0034774 | secretory granule lumen | 0.005946804 | 0.034631385 | 0.023198057 |
| CC | GO:0060205 | cytoplasmic vesicle lumen | 0.006856895 | 0.037006038 | 0.024788734 |
| CC | GO:0044853 | plasma membrane raft | 0.007102169 | 0.037006038 | 0.024788734 |
| CC | GO:1904813 | ficolin-1-rich granule lumen | 0.009104738 | 0.045068455 | 0.030189395 |
| MF | GO:0044389 | ubiquitin-like protein ligase binding | 8.60E-11 | 1.44E-08 | 5.71E-09 |
| MF | GO:0033613 | activating transcription factor binding | 9.70E-10 | 8.10E-08 | 3.22E-08 |
| MF | GO:0031625 | ubiquitin protein ligase binding | 1.87E-09 | 1.04E-07 | 4.13E-08 |
| MF | GO:0019902 | phosphatase binding | 2.59E-09 | 1.08E-07 | 4.30E-08 |
| MF | GO:0019903 | protein phosphatase binding | 1.99E-08 | 6.64E-07 | 2.64E-07 |
| MF | GO:0001085 | RNA polymerase II transcription factor binding | 3.66E-08 | 1.02E-06 | 4.04E-07 |
| MF | GO:0005126 | cytokine receptor binding | 5.26E-08 | 1.25E-06 | 4.98E-07 |
| MF | GO:0001047 | core promoter binding | 7.08E-07 | 1.48E-05 | 5.86E-06 |
| MF | GO:0001228 | DNA-binding transcription activator activity, RNA polymerase II-specific | 9.58E-07 | 1.78E-05 | 7.06E-06 |
| MF | GO:0070491 | repressing transcription factor binding | 1.99E-06 | 3.32E-05 | 1.32E-05 |
| MF | GO:0005125 | cytokine activity | 7.95E-06 | 0.000117138 | 4.65E-05 |
| MF | GO:0005164 | tumor necrosis factor receptor binding | 8.42E-06 | 0.000117138 | 4.65E-05 |
| MF | GO:0051721 | protein phosphatase 2A binding | 9.28E-06 | 0.000119212 | 4.73E-05 |
| MF | GO:0004712 | protein serine/threonine/tyrosine kinase activity | 2.29E-05 | 0.000272869 | 0.000108356 |
| MF | GO:0001046 | core promoter sequence-specific DNA binding | 2.63E-05 | 0.000292339 | 0.000116088 |
| MF | GO:0032813 | tumor necrosis factor receptor superfamily binding | 2.81E-05 | 0.00029294 | 0.000116327 |
| MF | GO:0035257 | nuclear hormone receptor binding | 4.08E-05 | 0.000399462 | 0.000158627 |
| MF | GO:0001102 | RNA polymerase II activating transcription factor binding | 4.31E-05 | 0.000399462 | 0.000158627 |
| MF | GO:0051427 | hormone receptor binding | 8.76E-05 | 0.000770171 | 0.000305835 |
| MF | GO:0004707 | MAP kinase activity | 0.000145644 | 0.00121613 | 0.000482926 |
| MF | GO:0004708 | MAP kinase kinase activity | 0.000191755 | 0.001524908 | 0.000605542 |
| MF | GO:0035258 | steroid hormone receptor binding | 0.000223306 | 0.001695098 | 0.000673124 |
| MF | GO:0000980 | RNA polymerase II distal enhancer sequence-specific DNA binding | 0.00027726 | 0.002013147 | 0.000799422 |
| MF | GO:0001091 | RNA polymerase II basal transcription factor binding | 0.000302653 | 0.00210596 | 0.000836278 |
| MF | GO:0048018 | receptor ligand activity | 0.000329026 | 0.002197897 | 0.000872786 |
| MF | GO:0042826 | histone deacetylase binding | 0.000388134 | 0.002397951 | 0.000952227 |
| MF | GO:0001223 | transcription coactivator binding | 0.000402052 | 0.002397951 | 0.000952227 |
| MF | GO:0070412 | R-SMAD binding | 0.000402052 | 0.002397951 | 0.000952227 |
| MF | GO:0001158 | enhancer sequence-specific DNA binding | 0.000475896 | 0.002740503 | 0.001088255 |
| MF | GO:0035035 | histone acetyltransferase binding | 0.000642137 | 0.003404972 | 0.001352116 |
| MF | GO:0051059 | NF-kappaB binding | 0.000642137 | 0.003404972 | 0.001352116 |
| MF | GO:0035326 | enhancer binding | 0.000658332 | 0.003404972 | 0.001352116 |
| MF | GO:0070851 | growth factor receptor binding | 0.000672839 | 0.003404972 | 0.001352116 |
| MF | GO:0097718 | disordered domain specific binding | 0.000832459 | 0.004088842 | 0.001623681 |
| MF | GO:0000979 | RNA polymerase II core promoter sequence-specific DNA binding | 0.000883789 | 0.004216936 | 0.001674547 |
| MF | GO:0030331 | estrogen receptor binding | 0.001347859 | 0.006252568 | 0.002482898 |
| MF | GO:0001221 | transcription cofactor binding | 0.001412492 | 0.006375304 | 0.002531637 |
| MF | GO:0042805 | actinin binding | 0.001615139 | 0.007037346 | 0.002794534 |
| MF | GO:0004879 | nuclear receptor activity | 0.001685592 | 0.007037346 | 0.002794534 |
| MF | GO:0098531 | transcription factor activity, direct ligand regulated sequence-specific DNA binding | 0.001685592 | 0.007037346 | 0.002794534 |
| MF | GO:1990841 | promoter-specific chromatin binding | 0.001757491 | 0.007158559 | 0.002842668 |
| MF | GO:0016538 | cyclin-dependent protein serine/threonine kinase regulator activity | 0.001830832 | 0.007279735 | 0.002890787 |
| MF | GO:0070888 | E-box binding | 0.001905611 | 0.007400863 | 0.002938887 |
| MF | GO:0005516 | calmodulin binding | 0.002131429 | 0.008089742 | 0.003212441 |
| MF | GO:0004674 | protein serine/threonine kinase activity | 0.002274758 | 0.008441881 | 0.003352276 |
| MF | GO:0003707 | steroid hormone receptor activity | 0.002384309 | 0.008471906 | 0.003364198 |
| MF | GO:0043621 | protein self-association | 0.002384309 | 0.008471906 | 0.003364198 |
| MF | GO:0097110 | scaffold protein binding | 0.002642797 | 0.009194731 | 0.003651233 |
| MF | GO:0042379 | chemokine receptor binding | 0.003294907 | 0.011229582 | 0.004459273 |
| MF | GO:0001227 | DNA-binding transcription repressor activity, RNA polymerase II-specific | 0.003653776 | 0.012203613 | 0.004846061 |
| MF | GO:0001098 | basal transcription machinery binding | 0.003907709 | 0.012549758 | 0.004983516 |
| MF | GO:0001099 | basal RNA polymerase II transcription machinery binding | 0.003907709 | 0.012549758 | 0.004983516 |
| MF | GO:0046332 | SMAD binding | 0.004800762 | 0.01512693 | 0.006006912 |
| MF | GO:0051219 | phosphoprotein binding | 0.005157729 | 0.015950755 | 0.006334054 |
| MF | GO:0003713 | transcription coactivator activity | 0.007865478 | 0.02388245 | 0.009483734 |
| MF | GO:0047485 | protein N-terminus binding | 0.008740336 | 0.026064929 | 0.010350397 |
| MF | GO:0002020 | protease binding | 0.011890626 | 0.034837449 | 0.01383397 |
| MF | GO:0005178 | integrin binding | 0.012608737 | 0.036304467 | 0.014416523 |
| MF | GO:0004955 | prostaglandin receptor activity | 0.012924074 | 0.0365817 | 0.014526613 |
| MF | GO:0001094 | TFIID-class transcription factor complex binding | 0.014207658 | 0.038269013 | 0.015196646 |
| MF | GO:0004954 | prostanoid receptor activity | 0.014207658 | 0.038269013 | 0.015196646 |
| MF | GO:0045236 | CXCR chemokine receptor binding | 0.014207658 | 0.038269013 | 0.015196646 |
| MF | GO:0004861 | cyclin-dependent protein serine/threonine kinase inhibitor activity | 0.015489645 | 0.040418291 | 0.016050125 |
| MF | GO:0042301 | phosphate ion binding | 0.015489645 | 0.040418291 | 0.016050125 |
| MF | GO:0005172 | vascular endothelial growth factor receptor binding | 0.016770037 | 0.042433275 | 0.016850276 |
| MF | GO:0016653 | oxidoreductase activity, acting on NAD(P)H, heme protein as acceptor | 0.016770037 | 0.042433275 | 0.016850276 |
| MF | GO:0035497 | cAMP response element binding | 0.018048836 | 0.044325819 | 0.017601806 |
| MF | GO:0036041 | long-chain fatty acid binding | 0.018048836 | 0.044325819 | 0.017601806 |
| MF | GO:0008083 | growth factor activity | 0.018791612 | 0.044825687 | 0.017800304 |
| MF | GO:0004953 | icosanoid receptor activity | 0.019326045 | 0.044825687 | 0.017800304 |
| MF | GO:0005161 | platelet-derived growth factor receptor binding | 0.019326045 | 0.044825687 | 0.017800304 |
| MF | GO:0097153 | cysteine-type endopeptidase activity involved in apoptotic process | 0.019326045 | 0.044825687 | 0.017800304 |
| MF | GO:0005149 | interleukin-1 receptor binding | 0.020601664 | 0.045269446 | 0.017976521 |
| MF | GO:0010181 | FMN binding | 0.020601664 | 0.045269446 | 0.017976521 |
| MF | GO:0032794 | GTPase activating protein binding | 0.020601664 | 0.045269446 | 0.017976521 |
| MF | GO:0071837 | HMG box domain binding | 0.020601664 | 0.045269446 | 0.017976521 |
| MF | GO:0005123 | death receptor binding | 0.021875696 | 0.046836427 | 0.01859877 |
| MF | GO:0046965 | retinoid X receptor binding | 0.021875696 | 0.046836427 | 0.01859877 |
| MF | GO:0019887 | protein kinase regulator activity | 0.022625694 | 0.047143169 | 0.018720578 |
| MF | GO:0008353 | RNA polymerase II CTD heptapeptide repeat kinase activity | 0.023148143 | 0.047143169 | 0.018720578 |
| MF | GO:0031435 | mitogen-activated protein kinase kinase kinase binding | 0.023148143 | 0.047143169 | 0.018720578 |
| MF | GO:0070064 | proline-rich region binding | 0.023148143 | 0.047143169 | 0.018720578 |
