# Supplementary material for: Study on the Mechanism of Compound Kidney-Invigorating Granule for Osteoporosis based on Network Pharmacology and Experimental Verification
Source: Evid Based Complement Alternat Med. 2022 Jan 4;2022:6453501. doi: 10.1155/2022/6453501 (PMC8752261; doi:10.1155/2022/6453501)
Supplement: Supplementary Materials — Supplementary Table 1: the abbreviations and degree values of bioactive ingredients of the “C-T” network. Supplementary Table 2: hub genes of treating OP of CKG. Supplementary Table 3: the results of GO enrichment analysis. Supplementary Table 4: the KEGG enrichment analysis results of the top 20 pathways with high correlation with OP. Supplementary File 5: the diagrams of the MAPK signaling pathway, PI3K-Akt signaling pathway, TNF signaling pathway, and the relationship diagram between them. Supplementary Table 6: docking scores of the top 10 bioactive ingredients of CKG with 5 core targets. Supplementary Table 7: the result of CCK-8. Supplementary Table 8: the results of KEGG enrichment analysis. [file 6453501.f1.zip › 6453501.f1/Supplementary Table 4 .docx]

| Pathway | GeneRatio | pvalue | Count |
| --- | --- | --- | --- |
| TNF signaling pathway | 0.4783 | 2.57E-15 | 11 |
| Osteoclast differentiation | 0.4783 | 1.16E-14 | 11 |
| Cellular senescence | 0.4783 | 1.07E-13 | 11 |
| MAPK signaling pathway | 0.5170 | 4.04E-12 | 12 |
| Apoptosis | 0.3913 | 5.52E-11 | 9 |
| PI3K-Akt signaling pathway | 0.4783 | 8.17E-10 | 11 |
| Rheumatoid arthritis | 0.3043 | 4.47E-09 | 7 |
| Thyroid hormone signaling pathway | 0.3043 | 2.83E-08 | 7 |
| FoxO signaling pathway | 0.3043 | 4.92E-08 | 7 |
| Estrogen signaling pathway | 0.3043 | 7.06E-08 | 7 |
| JAK-STAT signaling pathway | 0.3043 | 2.13E-07 | 7 |
| NF-kappa B signaling pathway | 0.2174 | 8.94E-06 | 5 |
| Growth hormone synthesis, secretion and action | 0.2174 | 1.73E-05 | 5 |
| Cell cycle | 0.2174 | 2.11E-05 | 5 |
| Adipocytokine signaling pathway | 0.1739 | 3.80E-05 | 4 |
| p53 signaling pathway | 0.1739 | 4.75E-05 | 4 |
| Longevity regulating pathway | 0.1739 | 0.000103 | 4 |
| Signaling pathways regulating pluripotency of stem cells | 0.1739 | 0.000639 | 4 |
| Necroptosis | 0.1739 | 0.000952 | 4 |
| Wnt signaling pathway | 0.1739 | 0.000974 | 4 |
