# Supplementary material for: Study on the Mechanism of Compound Kidney-Invigorating Granule for Osteoporosis based on Network Pharmacology and Experimental Verification
Source: Evid Based Complement Alternat Med. 2022 Jan 4;2022:6453501. doi: 10.1155/2022/6453501 (PMC8752261; doi:10.1155/2022/6453501)
Supplement: Supplementary Materials — Supplementary Table 1: the abbreviations and degree values of bioactive ingredients of the “C-T” network. Supplementary Table 2: hub genes of treating OP of CKG. Supplementary Table 3: the results of GO enrichment analysis. Supplementary Table 4: the KEGG enrichment analysis results of the top 20 pathways with high correlation with OP. Supplementary File 5: the diagrams of the MAPK signaling pathway, PI3K-Akt signaling pathway, TNF signaling pathway, and the relationship diagram between them. Supplementary Table 6: docking scores of the top 10 bioactive ingredients of CKG with 5 core targets. Supplementary Table 7: the result of CCK-8. Supplementary Table 8: the results of KEGG enrichment analysis. [file 6453501.f1.zip › 6453501.f1/Supplementary Table 6 .docx]

| **Target** | **Compound** | **Affinity (kcal/mol)** |
| --- | --- | --- |
| jun | kaempferol | -8.3 |
| jun | Quercetin | -8.6 |
| jun | 22,23-Dihydrostigmasterol | -8.9 |
| jun | luteolin | -8.8 |
| jun | Anhydroicaritin | -9.1 |
| jun | 8-(3-methylbut-2-enyl)-2-phenyl-chromone | -9.3 |
| jun | isorhamnetin | -8.5 |
| jun | formononetin | -8.6 |
| jun | Calycosin | -8.5 |
| jun | 7-O-methylisomucronulatol | -7.7 |
| TP53 | kaempferol | -7.1 |
| TP53 | Quercetin | -7.6 |
| TP53 | 22,23-Dihydrostigmasterol | -7 |
| TP53 | luteolin | -7.8 |
| TP53 | Anhydroicaritin | -7.7 |
| TP53 | 8-(3-methylbut-2-enyl)-2-phenyl-chromone | -7.5 |
| TP53 | isorhamnetin | -7.5 |
| TP53 | formononetin | -7.1 |
| TP53 | Calycosin | -7.1 |
| TP53 | 7-O-methylisomucronulatol | -6.9 |
| TNF | kaempferol | -7.8 |
| TNF | Quercetin | -8.4 |
| TNF | 22,23-Dihydrostigmasterol | -8.5 |
| TNF | luteolin | -9 |
| TNF | Anhydroicaritin | -8.1 |
| TNF | 8-(3-methylbut-2-enyl)-2-phenyl-chromone | -9 |
| TNF | isorhamnetin | -8 |
| TNF | formononetin | -7.9 |
| TNF | Calycosin | -8.1 |
| TNF | 7-O-methylisomucronulatol | -6.8 |
| AKT1 | kaempferol | -5.9 |
| AKT1 | Quercetin | -5.9 |
| AKT1 | 22,23-Dihydrostigmasterol | -7.4 |
| AKT1 | luteolin | -6.1 |
| AKT1 | Anhydroicaritin | -6.3 |
| AKT1 | 8-(3-methylbut-2-enyl)-2-phenyl-chromone | -6.7 |
| AKT1 | isorhamnetin | -6 |
| AKT1 | formononetin | -6.5 |
| AKT1 | Calycosin | -6.5 |
| AKT1 | 7-O-methylisomucronulatol | -5.9 |
| MAPK1 | kaempferol | -9.4 |
| MAPK1 | Quercetin | -9.4 |
| MAPK1 | 22,23-Dihydrostigmasterol | -9 |
| MAPK1 | luteolin | -9.3 |
| MAPK1 | Anhydroicaritin | -9.1 |
| MAPK1 | 8-(3-methylbut-2-enyl)-2-phenyl-chromone | -8.9 |
| MAPK1 | isorhamnetin | -9.2 |
| MAPK1 | formononetin | -8.9 |
| MAPK1 | Calycosin | -8.9 |
| MAPK1 | 7-O-methylisomucronulatol | -7.1 |
|  |  |  |
