# Supplementary material for: Study on the Mechanism of Compound Kidney-Invigorating Granule for Osteoporosis based on Network Pharmacology and Experimental Verification
Source: Evid Based Complement Alternat Med. 2022 Jan 4;2022:6453501. doi: 10.1155/2022/6453501 (PMC8752261; doi:10.1155/2022/6453501)
Supplement: Supplementary Materials — Supplementary Table 1: the abbreviations and degree values of bioactive ingredients of the “C-T” network. Supplementary Table 2: hub genes of treating OP of CKG. Supplementary Table 3: the results of GO enrichment analysis. Supplementary Table 4: the KEGG enrichment analysis results of the top 20 pathways with high correlation with OP. Supplementary File 5: the diagrams of the MAPK signaling pathway, PI3K-Akt signaling pathway, TNF signaling pathway, and the relationship diagram between them. Supplementary Table 6: docking scores of the top 10 bioactive ingredients of CKG with 5 core targets. Supplementary Table 7: the result of CCK-8. Supplementary Table 8: the results of KEGG enrichment analysis. [file 6453501.f1.zip › 6453501.f1/Supplementary Table 7 .docx]

| **Time** | **Group** | **OD Value** | | | | | |
| --- | --- | --- | --- | --- | --- | --- | --- |
| 24h | 2%NS | 1.237 | 1.175 | 1.178 | 1.248 | 1.199 | 1.218 |
| 24h | 2%MS | 1.22 | 1.210 | 1.216 | 1.265 | 1.225 | 1.19 |
| 24h | 5%NS | 1.107 | 1.007 | 1.097 | 0.979 | 1.07 | 1.085 |
| 24h | 5%MS | 1.126 | 1.102 | 1.113 | 1.116 | 1.252 | 1.232 |
| 24h | 8%NS | 0.906 | 0.934 | 0.968 | 0.991 | 1.011 | 0.869 |
| 24h | 8%MS | 1.036 | 0.954 | 1.088 | 0.946 | 1.028 | 1.024 |
| 36h | 2%NS | 1.552 | 1.416 | 1.712 | 1.52 | 1.44 | 1.4 |
| 36h | 2%MS | 1.596 | 1.543 | 1.626 | 1.617 | 1.569 | 1.474 |
| 36h | 5%NS | 1.404 | 1.186 | 1.23 | 1.081 | 1.395 | 1.303 |
| 36h | 5%MS | 1.485 | 1.43 | 1.477 | 1.34 | 1.415 | 1.412 |
| 36h | 8%NS | 1.198 | 1.316 | 1.2 | 1.258 | 1.286 | 1.252 |
| 36h | 8%MS | 1.356 | 1.273 | 1.361 | 1.403 | 1.322 | 1.42 |
| 48h | 2%NS | 1.602 | 1.607 | 1.603 | 1.654 | 1.650 | 1.554 |
| 48h | 2%MS | 1.625 | 1.705 | 1.623 | 1.707 | 1.62 | 1.71 |
| 48h | 5%NS | 1.34 | 1.403 | 1.501 | 1.399 | 1.506 | 1.407 |
| 48h | 5%MS | 1.545 | 1.582 | 1.507 | 1.504 | 1.555 | 1.607 |
| 48h | 8%NS | 1.414 | 1.433 | 1.39 | 1.41 | 1.395 | 1.44 |
| 48h | 8%MS | 1.5 | 1.513 | 1.5 | 1.467 | 1.52 | 1.54 |
