# Supplementary material for: Study on the Mechanism of Compound Kidney-Invigorating Granule for Osteoporosis based on Network Pharmacology and Experimental Verification
Source: Evid Based Complement Alternat Med. 2022 Jan 4;2022:6453501. doi: 10.1155/2022/6453501 (PMC8752261; doi:10.1155/2022/6453501)
Supplement: Supplementary Materials — Supplementary Table 1: the abbreviations and degree values of bioactive ingredients of the “C-T” network. Supplementary Table 2: hub genes of treating OP of CKG. Supplementary Table 3: the results of GO enrichment analysis. Supplementary Table 4: the KEGG enrichment analysis results of the top 20 pathways with high correlation with OP. Supplementary File 5: the diagrams of the MAPK signaling pathway, PI3K-Akt signaling pathway, TNF signaling pathway, and the relationship diagram between them. Supplementary Table 6: docking scores of the top 10 bioactive ingredients of CKG with 5 core targets. Supplementary Table 7: the result of CCK-8. Supplementary Table 8: the results of KEGG enrichment analysis. [file 6453501.f1.zip › 6453501.f1/Supplementary Table 8 .docx]

| ID | Description | GeneRatio | BgRatio | pvalue | p.adjust | qvalue | Count |
| --- | --- | --- | --- | --- | --- | --- | --- |
| hsa05167 | Kaposi sarcoma-associated herpesvirus infection | 17/23 | 193/8087 | 1.16E-23 | 2.09E-21 | 5.00E-22 | 17 |
| hsa05161 | Hepatitis B | 16/23 | 162/8087 | 6.90E-23 | 6.21E-21 | 1.49E-21 | 16 |
| hsa05163 | Human cytomegalovirus infection | 17/23 | 225/8087 | 1.71E-22 | 1.03E-20 | 2.46E-21 | 17 |
| hsa04933 | AGE-RAGE signaling pathway in diabetic complications | 13/23 | 100/8087 | 7.31E-20 | 3.29E-18 | 7.89E-19 | 13 |
| hsa04620 | Toll-like receptor signaling pathway | 13/23 | 104/8087 | 1.25E-19 | 4.51E-18 | 1.08E-18 | 13 |
| hsa05142 | Chagas disease | 12/23 | 102/8087 | 1.01E-17 | 3.02E-16 | 7.24E-17 | 12 |
| hsa05169 | Epstein-Barr virus infection | 14/23 | 202/8087 | 1.57E-17 | 4.05E-16 | 9.71E-17 | 14 |
| hsa05160 | Hepatitis C | 13/23 | 157/8087 | 3.26E-17 | 7.35E-16 | 1.76E-16 | 13 |
| hsa04657 | IL-17 signaling pathway | 11/23 | 94/8087 | 3.46E-16 | 6.92E-15 | 1.66E-15 | 11 |
| hsa01522 | Endocrine resistance | 11/23 | 98/8087 | 5.59E-16 | 1.01E-14 | 2.41E-15 | 11 |
| hsa05219 | Bladder cancer | 9/23 | 41/8087 | 6.71E-16 | 1.10E-14 | 2.63E-15 | 9 |
| hsa04625 | C-type lectin receptor signaling pathway | 11/23 | 104/8087 | 1.10E-15 | 1.65E-14 | 3.97E-15 | 11 |
| hsa04668 | TNF signaling pathway | 11/23 | 112/8087 | 2.57E-15 | 3.42E-14 | 8.21E-15 | 11 |
| hsa05166 | Human T-cell leukemia virus 1 infection | 13/23 | 219/8087 | 2.66E-15 | 3.42E-14 | 8.21E-15 | 13 |
| hsa05212 | Pancreatic cancer | 10/23 | 76/8087 | 3.02E-15 | 3.63E-14 | 8.70E-15 | 10 |
| hsa04380 | Osteoclast differentiation | 11/23 | 128/8087 | 1.16E-14 | 1.31E-13 | 3.14E-14 | 11 |
| hsa05132 | Salmonella infection | 13/23 | 249/8087 | 1.43E-14 | 1.51E-13 | 3.62E-14 | 13 |
| hsa05135 | Yersinia infection | 11/23 | 137/8087 | 2.50E-14 | 2.50E-13 | 6.00E-14 | 11 |
| hsa05162 | Measles | 11/23 | 139/8087 | 2.94E-14 | 2.79E-13 | 6.69E-14 | 11 |
| hsa05224 | Breast cancer | 11/23 | 147/8087 | 5.51E-14 | 4.96E-13 | 1.19E-13 | 11 |
| hsa04218 | Cellular senescence | 11/23 | 156/8087 | 1.07E-13 | 9.17E-13 | 2.20E-13 | 11 |
| hsa05171 | Coronavirus disease - COVID-19 | 12/23 | 232/8087 | 2.40E-13 | 1.94E-12 | 4.64E-13 | 12 |
| hsa05133 | Pertussis | 9/23 | 76/8087 | 2.58E-13 | 1.94E-12 | 4.64E-13 | 9 |
| hsa05220 | Chronic myeloid leukemia | 9/23 | 76/8087 | 2.58E-13 | 1.94E-12 | 4.64E-13 | 9 |
| hsa05140 | Leishmaniasis | 9/23 | 77/8087 | 2.92E-13 | 2.10E-12 | 5.04E-13 | 9 |
| hsa04621 | NOD-like receptor signaling pathway | 11/23 | 181/8087 | 5.58E-13 | 3.86E-12 | 9.26E-13 | 11 |
| hsa04926 | Relaxin signaling pathway | 10/23 | 129/8087 | 7.20E-13 | 4.80E-12 | 1.15E-12 | 10 |
| hsa05210 | Colorectal cancer | 9/23 | 86/8087 | 8.21E-13 | 5.28E-12 | 1.26E-12 | 9 |
| hsa05235 | PD-L1 expression and PD-1 checkpoint pathway in cancer | 9/23 | 89/8087 | 1.13E-12 | 7.01E-12 | 1.68E-12 | 9 |
| hsa05130 | Pathogenic Escherichia coli infection | 11/23 | 197/8087 | 1.42E-12 | 8.53E-12 | 2.05E-12 | 11 |
| hsa05418 | Fluid shear stress and atherosclerosis | 10/23 | 139/8087 | 1.54E-12 | 8.92E-12 | 2.14E-12 | 10 |
| hsa05205 | Proteoglycans in cancer | 11/23 | 205/8087 | 2.20E-12 | 1.24E-11 | 2.97E-12 | 11 |
| hsa05215 | Prostate cancer | 9/23 | 97/8087 | 2.51E-12 | 1.37E-11 | 3.28E-12 | 9 |
| hsa04010 | MAPK signaling pathway | 12/23 | 294/8087 | 4.04E-12 | 2.14E-11 | 5.12E-12 | 12 |
| hsa04659 | Th17 cell differentiation | 9/23 | 107/8087 | 6.19E-12 | 3.18E-11 | 7.63E-12 | 9 |
| hsa04917 | Prolactin signaling pathway | 8/23 | 70/8087 | 9.24E-12 | 4.62E-11 | 1.11E-11 | 8 |
| hsa05164 | Influenza A | 10/23 | 172/8087 | 1.31E-11 | 6.39E-11 | 1.53E-11 | 10 |
| hsa05131 | Shigellosis | 11/23 | 246/8087 | 1.62E-11 | 7.56E-11 | 1.81E-11 | 11 |
| hsa05165 | Human papillomavirus infection | 12/23 | 331/8087 | 1.64E-11 | 7.56E-11 | 1.81E-11 | 12 |
| hsa04210 | Apoptosis | 9/23 | 136/8087 | 5.52E-11 | 2.48E-10 | 5.96E-11 | 9 |
| hsa05222 | Small cell lung cancer | 8/23 | 92/8087 | 8.78E-11 | 3.86E-10 | 9.24E-11 | 8 |
| hsa05134 | Legionellosis | 7/23 | 57/8087 | 1.33E-10 | 5.60E-10 | 1.34E-10 | 7 |
| hsa04932 | Non-alcoholic fatty liver disease | 9/23 | 150/8087 | 1.34E-10 | 5.60E-10 | 1.34E-10 | 9 |
| hsa05213 | Endometrial cancer | 7/23 | 58/8087 | 1.51E-10 | 6.16E-10 | 1.48E-10 | 7 |
| hsa04660 | T cell receptor signaling pathway | 8/23 | 104/8087 | 2.38E-10 | 9.53E-10 | 2.28E-10 | 8 |
| hsa04066 | HIF-1 signaling pathway | 8/23 | 109/8087 | 3.48E-10 | 1.36E-09 | 3.27E-10 | 8 |
| hsa05145 | Toxoplasmosis | 8/23 | 112/8087 | 4.34E-10 | 1.66E-09 | 3.98E-10 | 8 |
| hsa05216 | Thyroid cancer | 6/23 | 37/8087 | 5.72E-10 | 2.15E-09 | 5.14E-10 | 6 |
| hsa05152 | Tuberculosis | 9/23 | 180/8087 | 6.86E-10 | 2.52E-09 | 6.04E-10 | 9 |
| hsa05218 | Melanoma | 7/23 | 72/8087 | 7.21E-10 | 2.54E-09 | 6.10E-10 | 7 |
| hsa05223 | Non-small cell lung cancer | 7/23 | 72/8087 | 7.21E-10 | 2.54E-09 | 6.10E-10 | 7 |
| hsa04151 | PI3K-Akt signaling pathway | 11/23 | 354/8087 | 8.17E-10 | 2.83E-09 | 6.78E-10 | 11 |
| hsa05214 | Glioma | 7/23 | 75/8087 | 9.66E-10 | 3.28E-09 | 7.86E-10 | 7 |
| hsa05203 | Viral carcinogenesis | 9/23 | 204/8087 | 2.09E-09 | 6.96E-09 | 1.67E-09 | 9 |
| hsa05170 | Human immunodeficiency virus 1 infection | 9/23 | 212/8087 | 2.93E-09 | 9.60E-09 | 2.30E-09 | 9 |
| hsa04658 | Th1 and Th2 cell differentiation | 7/23 | 92/8087 | 4.14E-09 | 1.33E-08 | 3.19E-09 | 7 |
| hsa05226 | Gastric cancer | 8/23 | 149/8087 | 4.27E-09 | 1.35E-08 | 3.23E-09 | 8 |
| hsa05323 | Rheumatoid arthritis | 7/23 | 93/8087 | 4.47E-09 | 1.39E-08 | 3.32E-09 | 7 |
| hsa05225 | Hepatocellular carcinoma | 8/23 | 168/8087 | 1.10E-08 | 3.37E-08 | 8.08E-09 | 8 |
| hsa05321 | Inflammatory bowel disease | 6/23 | 65/8087 | 1.93E-08 | 5.80E-08 | 1.39E-08 | 6 |
| hsa04071 | Sphingolipid signaling pathway | 7/23 | 119/8087 | 2.52E-08 | 7.33E-08 | 1.76E-08 | 7 |
| hsa04722 | Neurotrophin signaling pathway | 7/23 | 119/8087 | 2.52E-08 | 7.33E-08 | 1.76E-08 | 7 |
| hsa04919 | Thyroid hormone signaling pathway | 7/23 | 121/8087 | 2.83E-08 | 8.10E-08 | 1.94E-08 | 7 |
| hsa04622 | RIG-I-like receptor signaling pathway | 6/23 | 70/8087 | 3.04E-08 | 8.42E-08 | 2.02E-08 | 6 |
| hsa05120 | Epithelial cell signaling in Helicobacter pylori infection | 6/23 | 70/8087 | 3.04E-08 | 8.42E-08 | 2.02E-08 | 6 |
| hsa04068 | FoxO signaling pathway | 7/23 | 131/8087 | 4.92E-08 | 1.34E-07 | 3.22E-08 | 7 |
| hsa04915 | Estrogen signaling pathway | 7/23 | 138/8087 | 7.06E-08 | 1.90E-07 | 4.55E-08 | 7 |
| hsa04662 | B cell receptor signaling pathway | 6/23 | 82/8087 | 7.94E-08 | 2.10E-07 | 5.04E-08 | 6 |
| hsa04012 | ErbB signaling pathway | 6/23 | 85/8087 | 9.87E-08 | 2.57E-07 | 6.17E-08 | 6 |
| hsa04921 | Oxytocin signaling pathway | 7/23 | 154/8087 | 1.50E-07 | 3.87E-07 | 9.27E-08 | 7 |
| hsa04630 | JAK-STAT signaling pathway | 7/23 | 162/8087 | 2.13E-07 | 5.40E-07 | 1.29E-07 | 7 |
| hsa04931 | Insulin resistance | 6/23 | 108/8087 | 4.14E-07 | 1.04E-06 | 2.48E-07 | 6 |
| hsa04370 | VEGF signaling pathway | 5/23 | 59/8087 | 5.29E-07 | 1.30E-06 | 3.13E-07 | 5 |
| hsa05202 | Transcriptional misregulation in cancer | 7/23 | 192/8087 | 6.77E-07 | 1.65E-06 | 3.95E-07 | 7 |
| hsa05221 | Acute myeloid leukemia | 5/23 | 67/8087 | 1.01E-06 | 2.41E-06 | 5.79E-07 | 5 |
| hsa05211 | Renal cell carcinoma | 5/23 | 69/8087 | 1.17E-06 | 2.76E-06 | 6.62E-07 | 5 |
| hsa05230 | Central carbon metabolism in cancer | 5/23 | 70/8087 | 1.25E-06 | 2.93E-06 | 7.03E-07 | 5 |
| hsa01523 | Antifolate resistance | 4/23 | 31/8087 | 1.49E-06 | 3.43E-06 | 8.23E-07 | 4 |
| hsa01524 | Platinum drug resistance | 5/23 | 73/8087 | 1.55E-06 | 3.52E-06 | 8.45E-07 | 5 |
| hsa01521 | EGFR tyrosine kinase inhibitor resistance | 5/23 | 79/8087 | 2.30E-06 | 5.16E-06 | 1.24E-06 | 5 |
| hsa05168 | Herpes simplex virus 1 infection | 9/23 | 498/8087 | 4.45E-06 | 9.88E-06 | 2.37E-06 | 9 |
| hsa05231 | Choline metabolism in cancer | 5/23 | 98/8087 | 6.68E-06 | 1.47E-05 | 3.51E-06 | 5 |
| hsa05146 | Amoebiasis | 5/23 | 102/8087 | 8.13E-06 | 1.76E-05 | 4.23E-06 | 5 |
| hsa04064 | NF-kappa B signaling pathway | 5/23 | 104/8087 | 8.94E-06 | 1.92E-05 | 4.59E-06 | 5 |
| hsa05144 | Malaria | 4/23 | 50/8087 | 1.05E-05 | 2.22E-05 | 5.33E-06 | 4 |
| hsa04062 | Chemokine signaling pathway | 6/23 | 192/8087 | 1.19E-05 | 2.50E-05 | 5.99E-06 | 6 |
| hsa04510 | Focal adhesion | 6/23 | 201/8087 | 1.55E-05 | 3.21E-05 | 7.70E-06 | 6 |
| hsa05206 | MicroRNAs in cancer | 7/23 | 310/8087 | 1.64E-05 | 3.36E-05 | 8.04E-06 | 7 |
| hsa04935 | Growth hormone synthesis, secretion and action | 5/23 | 119/8087 | 1.73E-05 | 3.49E-05 | 8.37E-06 | 5 |
| hsa04110 | Cell cycle | 5/23 | 124/8087 | 2.11E-05 | 4.22E-05 | 1.01E-05 | 5 |
| hsa04024 | cAMP signaling pathway | 6/23 | 216/8087 | 2.34E-05 | 4.62E-05 | 1.11E-05 | 6 |
| hsa04623 | Cytosolic DNA-sensing pathway | 4/23 | 63/8087 | 2.65E-05 | 5.19E-05 | 1.24E-05 | 4 |
| hsa04664 | Fc epsilon RI signaling pathway | 4/23 | 68/8087 | 3.59E-05 | 6.95E-05 | 1.67E-05 | 4 |
| hsa04920 | Adipocytokine signaling pathway | 4/23 | 69/8087 | 3.80E-05 | 7.28E-05 | 1.75E-05 | 4 |
| hsa04115 | p53 signaling pathway | 4/23 | 73/8087 | 4.75E-05 | 9.00E-05 | 2.16E-05 | 4 |
| hsa05010 | Alzheimer disease | 7/23 | 369/8087 | 5.05E-05 | 9.48E-05 | 2.27E-05 | 7 |
| hsa04934 | Cushing syndrome | 5/23 | 155/8087 | 6.17E-05 | 0.000114539 | 2.75E-05 | 5 |
| hsa04211 | Longevity regulating pathway | 4/23 | 89/8087 | 0.000103443 | 0.000189997 | 4.56E-05 | 4 |
| hsa04912 | GnRH signaling pathway | 4/23 | 93/8087 | 0.000122769 | 0.000223216 | 5.35E-05 | 4 |
| hsa05143 | African trypanosomiasis | 3/23 | 37/8087 | 0.000146615 | 0.000263906 | 6.33E-05 | 3 |
| hsa04928 | Parathyroid hormone synthesis, secretion and action | 4/23 | 106/8087 | 0.000203822 | 0.000363246 | 8.71E-05 | 4 |
| hsa05332 | Graft-versus-host disease | 3/23 | 42/8087 | 0.000214619 | 0.00037874 | 9.08E-05 | 3 |
| hsa05022 | Pathways of neurodegeneration - multiple diseases | 7/23 | 475/8087 | 0.000247963 | 0.000433334 | 0.000103899 | 7 |
| hsa04015 | Rap1 signaling pathway | 5/23 | 210/8087 | 0.000258575 | 0.000447533 | 0.000107303 | 5 |
| hsa04611 | Platelet activation | 4/23 | 124/8087 | 0.000372055 | 0.000637809 | 0.000152925 | 4 |
| hsa04014 | Ras signaling pathway | 5/23 | 232/8087 | 0.000410183 | 0.000696537 | 0.000167006 | 5 |
| hsa04371 | Apelin signaling pathway | 4/23 | 137/8087 | 0.000543473 | 0.000914253 | 0.000219207 | 4 |
| hsa04550 | Signaling pathways regulating pluripotency of stem cells | 4/23 | 143/8087 | 0.000639047 | 0.001065078 | 0.00025537 | 4 |
| hsa04072 | Phospholipase D signaling pathway | 4/23 | 148/8087 | 0.000727381 | 0.001201179 | 0.000288002 | 4 |
| hsa05020 | Prion disease | 5/23 | 273/8087 | 0.000862412 | 0.00141122 | 0.000338363 | 5 |
| hsa04137 | Mitophagy - animal | 3/23 | 68/8087 | 0.000892816 | 0.00144781 | 0.000347136 | 3 |
| hsa04217 | Necroptosis | 4/23 | 159/8087 | 0.000951745 | 0.001529591 | 0.000366744 | 4 |
| hsa04310 | Wnt signaling pathway | 4/23 | 160/8087 | 0.000974308 | 0.001551994 | 0.000372116 | 4 |
| hsa04613 | Neutrophil extracellular trap formation | 4/23 | 190/8087 | 0.001841747 | 0.002908022 | 0.000697245 | 4 |
| hsa04350 | TGF-beta signaling pathway | 3/23 | 94/8087 | 0.002275665 | 0.00356191 | 0.000854025 | 3 |
| hsa04640 | Hematopoietic cell lineage | 3/23 | 99/8087 | 0.002638279 | 0.004093881 | 0.000981574 | 3 |
| hsa04061 | Viral protein interaction with cytokine and cytokine receptor | 3/23 | 100/8087 | 0.002714843 | 0.004141285 | 0.00099294 | 3 |
| hsa04914 | Progesterone-mediated oocyte maturation | 3/23 | 100/8087 | 0.002714843 | 0.004141285 | 0.00099294 | 3 |
| hsa04725 | Cholinergic synapse | 3/23 | 113/8087 | 0.003837509 | 0.005804636 | 0.001391755 | 3 |
| hsa04152 | AMPK signaling pathway | 3/23 | 120/8087 | 0.00454374 | 0.006815609 | 0.001634152 | 3 |
| hsa04728 | Dopaminergic synapse | 3/23 | 132/8087 | 0.005928561 | 0.008819346 | 0.00211458 | 3 |
| hsa04940 | Type I diabetes mellitus | 2/23 | 43/8087 | 0.006509314 | 0.009603906 | 0.002302691 | 2 |
| hsa04930 | Type II diabetes mellitus | 2/23 | 46/8087 | 0.007422358 | 0.010861987 | 0.002604336 | 2 |
| hsa05030 | Cocaine addiction | 2/23 | 49/8087 | 0.008390036 | 0.012150854 | 0.002913363 | 2 |
| hsa04261 | Adrenergic signaling in cardiomyocytes | 3/23 | 150/8087 | 0.008438093 | 0.012150854 | 0.002913363 | 3 |
| hsa04060 | Cytokine-cytokine receptor interaction | 4/23 | 295/8087 | 0.008876413 | 0.012680591 | 0.003040376 | 4 |
| hsa04150 | mTOR signaling pathway | 3/23 | 155/8087 | 0.009230713 | 0.013082901 | 0.003136836 | 3 |
| hsa04022 | cGMP-PKG signaling pathway | 3/23 | 167/8087 | 0.0113076 | 0.015901313 | 0.003812596 | 3 |
| hsa05416 | Viral myocarditis | 2/23 | 60/8087 | 0.012390975 | 0.017289733 | 0.004145492 | 2 |
| hsa05217 | Basal cell carcinoma | 2/23 | 63/8087 | 0.013601712 | 0.01883314 | 0.004515548 | 2 |
| hsa04929 | GnRH secretion | 2/23 | 64/8087 | 0.014016356 | 0.019259114 | 0.004617682 | 2 |
| hsa05031 | Amphetamine addiction | 2/23 | 69/8087 | 0.016171127 | 0.022051537 | 0.005287211 | 2 |
| hsa04520 | Adherens junction | 2/23 | 71/8087 | 0.017070509 | 0.023102944 | 0.005539302 | 2 |
| hsa04540 | Gap junction | 2/23 | 88/8087 | 0.025540224 | 0.034307764 | 0.008225838 | 2 |
| hsa05410 | Hypertrophic cardiomyopathy | 2/23 | 90/8087 | 0.026629743 | 0.035506324 | 0.008513212 | 2 |
| hsa04020 | Calcium signaling pathway | 3/23 | 240/8087 | 0.029496912 | 0.03904003 | 0.009360475 | 3 |
| hsa04666 | Fc gamma R-mediated phagocytosis | 2/23 | 97/8087 | 0.030589444 | 0.039899275 | 0.009566493 | 2 |
| hsa04713 | Circadian entrainment | 2/23 | 97/8087 | 0.030589444 | 0.039899275 | 0.009566493 | 2 |
| hsa04750 | Inflammatory mediator regulation of TRP channels | 2/23 | 98/8087 | 0.031173327 | 0.040368337 | 0.009678958 | 2 |
| hsa04114 | Oocyte meiosis | 2/23 | 129/8087 | 0.051356564 | 0.066029869 | 0.015831723 | 2 |
| hsa04650 | Natural killer cell mediated cytotoxicity | 2/23 | 131/8087 | 0.052787525 | 0.06738833 | 0.016157436 | 2 |
| hsa05016 | Huntington disease | 3/23 | 306/8087 | 0.054299997 | 0.068830982 | 0.016503335 | 3 |
| hsa04140 | Autophagy - animal | 2/23 | 137/8087 | 0.057166268 | 0.071457835 | 0.017133165 | 2 |
| hsa04910 | Insulin signaling pathway | 2/23 | 137/8087 | 0.057166268 | 0.071457835 | 0.017133165 | 2 |
| hsa00220 | Arginine biosynthesis | 1/23 | 22/8087 | 0.060812667 | 0.075491587 | 0.018100322 | 1 |
| hsa04723 | Retrograde endocannabinoid signaling | 2/23 | 148/8087 | 0.065514351 | 0.080771118 | 0.019366174 | 2 |
| hsa04390 | Hippo signaling pathway | 2/23 | 157/8087 | 0.072634928 | 0.088940728 | 0.02132497 | 2 |
| hsa05014 | Amyotrophic lateral sclerosis | 3/23 | 364/8087 | 0.082315845 | 0.099671127 | 0.023897756 | 3 |
| hsa04530 | Tight junction | 2/23 | 169/8087 | 0.082505544 | 0.099671127 | 0.023897756 | 2 |
| hsa05310 | Asthma | 1/23 | 31/8087 | 0.084656816 | 0.10158818 | 0.0243574 | 1 |
| hsa04215 | Apoptosis - multiple species | 1/23 | 32/8087 | 0.087270135 | 0.104030624 | 0.024943015 | 1 |
| hsa04960 | Aldosterone-regulated sodium reabsorption | 1/23 | 37/8087 | 0.100230035 | 0.118693463 | 0.028458667 | 1 |
| hsa05330 | Allograft rejection | 1/23 | 38/8087 | 0.102800807 | 0.120942125 | 0.02899782 | 1 |
| hsa04216 | Ferroptosis | 1/23 | 41/8087 | 0.110471034 | 0.129121988 | 0.030959073 | 1 |
| hsa04973 | Carbohydrate digestion and absorption | 1/23 | 47/8087 | 0.125623743 | 0.145885637 | 0.034978428 | 1 |
| hsa04810 | Regulation of actin cytoskeleton | 2/23 | 218/8087 | 0.126561517 | 0.146032519 | 0.035013645 | 2 |
| hsa04672 | Intestinal immune network for IgA production | 1/23 | 49/8087 | 0.130619548 | 0.149754896 | 0.035906145 | 1 |
| hsa00330 | Arginine and proline metabolism | 1/23 | 51/8087 | 0.135588043 | 0.154467391 | 0.037036041 | 1 |
| hsa04714 | Thermogenesis | 2/23 | 231/8087 | 0.139074522 | 0.157442855 | 0.037749457 | 2 |
| hsa04961 | Endocrine and other factor-regulated calcium reabsorption | 1/23 | 53/8087 | 0.140529371 | 0.158095543 | 0.037905949 | 1 |
| hsa04340 | Hedgehog signaling pathway | 1/23 | 56/8087 | 0.147890736 | 0.16534368 | 0.039643806 | 1 |
| hsa04923 | Regulation of lipolysis in adipocytes | 1/23 | 57/8087 | 0.150331093 | 0.167034548 | 0.040049219 | 1 |
| hsa04730 | Long-term depression | 1/23 | 60/8087 | 0.157612121 | 0.174050195 | 0.041731333 | 1 |
| hsa04213 | Longevity regulating pathway - multiple species | 1/23 | 62/8087 | 0.162432943 | 0.178280059 | 0.042745511 | 1 |
| hsa04720 | Long-term potentiation | 1/23 | 67/8087 | 0.174369817 | 0.190221618 | 0.045608692 | 1 |
| hsa03320 | PPAR signaling pathway | 1/23 | 76/8087 | 0.195447293 | 0.2119308 | 0.050813818 | 1 |
| hsa04612 | Antigen processing and presentation | 1/23 | 78/8087 | 0.200060775 | 0.215634368 | 0.051701808 | 1 |
| hsa05414 | Dilated cardiomyopathy | 1/23 | 96/8087 | 0.240458893 | 0.257634528 | 0.061772021 | 1 |
| hsa04916 | Melanogenesis | 1/23 | 101/8087 | 0.251329557 | 0.267688286 | 0.064182571 | 1 |
| hsa04922 | Glucagon signaling pathway | 1/23 | 107/8087 | 0.264177964 | 0.279717844 | 0.067066852 | 1 |
| hsa04670 | Leukocyte transendothelial migration | 1/23 | 114/8087 | 0.278901236 | 0.291873386 | 0.069981338 | 1 |
| hsa04724 | Glutamatergic synapse | 1/23 | 114/8087 | 0.278901236 | 0.291873386 | 0.069981338 | 1 |
| hsa04726 | Serotonergic synapse | 1/23 | 115/8087 | 0.280981415 | 0.292350606 | 0.070095759 | 1 |
| hsa04270 | Vascular smooth muscle contraction | 1/23 | 133/8087 | 0.317457516 | 0.328404327 | 0.078740219 | 1 |
| hsa05322 | Systemic lupus erythematosus | 1/23 | 136/8087 | 0.323362126 | 0.332601044 | 0.079746449 | 1 |
| hsa05017 | Spinocerebellar ataxia | 1/23 | 143/8087 | 0.336950137 | 0.344608094 | 0.082625333 | 1 |
| hsa04360 | Axon guidance | 1/23 | 182/8087 | 0.408004352 | 0.41491968 | 0.099483666 | 1 |
| hsa05034 | Alcoholism | 1/23 | 187/8087 | 0.416568744 | 0.421249292 | 0.101001292 | 1 |
| hsa05012 | Parkinson disease | 1/23 | 249/8087 | 0.513394152 | 0.516245958 | 0.123778271 | 1 |
| hsa04144 | Endocytosis | 1/23 | 251/8087 | 0.516245958 | 0.516245958 | 0.123778271 | 1 |
